# Loss of H3K27 methylation identifies poor outcomes in adult-onset acute leukemia

**DOI:** 10.1186/s13148-021-01011-x

**Published:** 2021-01-28

**Authors:** A. D. van Dijk, F. W. Hoff, Y. H. Qiu, J. Chandra, E. Jabbour, E. S. J. M. de Bont, T. M. Horton, S. M. Kornblau

**Affiliations:** 1 Department of Pediatric Oncology/Hematology, University Medical Center Groningen, Groningen, The Netherlands; 2 Department of Leukemia, The University of Texas MD Anderson Cancer Center, Houston, TX USA; 3 Department of Pediatrics, The University of Texas MD Anderson Cancer Center, Houston, TX USA; 4 Department of Pediatrics, Division of Hematology/Oncology, Baylor College of Medicine, Texas Children’s Cancer Center, Houston, TX USA

**Keywords:** Histone methylation, H3K27me3, H3K4me2, H3K4me3, RPPA, Proteomics, Acute leukemia, AML, ALL, Predictive markers

## Abstract

**Background:**

Acute leukemia is an epigenetically heterogeneous disease. The intensity of treatment is currently guided by cytogenetic and molecular genetic risk classifications; however these incompletely predict outcomes, requiring additional information for more accurate outcome predictions. We aimed to identify potential prognostic implications of epigenetic modification of histone proteins, with a focus on H3K4 and H3K27 methylation marks in relation to mutations in chromatin, splicing and transcriptional regulators in adult-onset acute lymphoblastic and myeloid leukemia.

**Results:**

Histone 3 lysine 4 di- and trimethylation (H3K4me2, H3K4me3) and lysine 27 trimethylation (H3K27me3) mark expression was evaluated in 241 acute myeloid leukemia (AML), 114 B-cell acute lymphoblastic leukemia (B-ALL) and 14T-cell ALL (T-ALL) patient samples at time of diagnosis using reverse phase protein array. Expression levels of the marks were significantly lower in AML than in B and T-ALL in both bone marrow and peripheral blood, as well as compared to normal CD34+ cells. In AML, greater loss of H3K27me3 was associated with increased proliferative potential and shorter overall survival in the whole patient population, as well as in subsets with DNA methylation mutations. To study the prognostic impact of H3K27me3 in the context of cytogenetic aberrations and mutations, multivariate analysis was performed and identified lower H3K27me3 level as an independent unfavorable prognostic factor in all, as well as in TP53 mutated patients. AML with decreased H3K27me3 demonstrated an upregulated anti-apoptotic phenotype. In ALL, the relative quantity of histone methylation expression correlated with response to tyrosine kinase inhibitor in patients who carried the Philadelphia cytogenetic aberration and prior smoking behavior.

**Conclusion:**

This study shows that proteomic profiling of epigenetic modifications has clinical implications in acute leukemia and supports the idea that epigenetic patterns contribute to a more accurate picture of the leukemic state that complements cytogenetic and molecular genetic subgrouping. A combination of these variables may offer more accurate outcome prediction and we suggest that histone methylation mark measurement at time of diagnosis might be a suitable method to improve patient outcome prediction and subsequent treatment intensity stratification in selected subgroups.

## Background

Acute leukemia is characterized by the malignant accumulation of poorly differentiated blood forming cells in the bone marrow (BM) and peripheral blood (PB). During diagnostic practice, immunophenotyping of leukemic blasts results in a diagnosis of acute lymphoblastic leukemia (ALL) that originates from lymphoid precursors or acute myeloid leukemia (AML) that arises from myeloid precursors [1]. The identification of cell lineage is important as it directs the choice of therapy. The intensity of treatment is further guided by cytogenetic and somatic genetic risk stratifications. Yet, these classifications lack precise outcome prediction capability leaving patients survival variable [2].

Efforts to improve disease classification in acute leukemia include genome-wide molecular profiling studies [3–6]. Among identified mutations in adult-onset AML, almost 20% of mutations are in genes that regulate chromatin modification and RNA splicing [6]. These contribute to the development and maintenance of leukemia as they disturb normal gene transcription via aberrant chromatin remodeling. Mutated epigenetic modifiers often exist in overlapping patterns of co-occurring mutations that lead to prognostically distinct phenotypes, thereby complicating individual treatment stratification [6–9]. Epigenetic dysregulation by histone protein modifications is also well-recognized to play a role in tumor development. Loss of the repressive mark H3K27me3 has been reported in breast, colon, ovarian, pancreatic, prostate, nervous system tumors and melanoma [10–16]. H3K4 di- and trimethylation are associated with open and active chromatin and are predictive factors for outcome in different cancers with low expression of H3K4me2 being associated with poor outcome in prostate, kidney and lung cancer and high expression of H3K4me3 in liver and cervical cancer [17, 18].

Here, we hypothesized that quantitative analysis of epigenetic modifications of histone modifiers could have clinical implications in genetic subgroups within acute leukemia. To study this, we generated reverse phase protein arrays (RPPA) of BM and PB derived adult AML, B-cell ALL (B-ALL) and T-cell ALL (T-ALL) samples and performed whole genome sequencing on a subset of the AML BM aspirates. RPPA provides a high-throughput, quantitative functional proteomic platform that enables identification of aberrant expressed proteins and the pathways they act in. Using RPPA, the AML Proteome Atlas has been built and led to the discovery that current cytogenetic and molecular classifications and the proteome barely overlap [10]. Since the proteome reflects the functional state of cells, analyzing histone modifications in genetic subgroups along with recognition of dysregulated pathways, achieved by proteomics, may reveal new epigenetic insights that can help us further improve outcome prognostication in acute leukemia.

## Results

### Proteomic profiling of histone methylation marks distinguishes acute lymphoblastic from myeloid leukemia patients

**Fig. 1:**
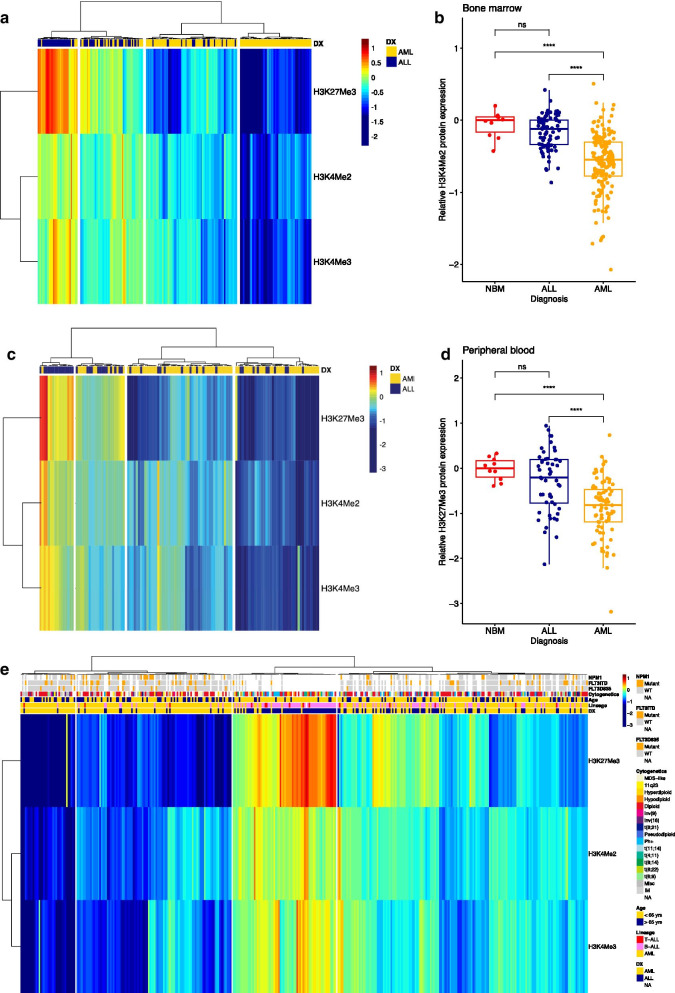
**a** Heatmap showing histone methylation levels in bone marrow (BM) derived samples from AML (*n* = 162) and ALL (*n* = 79) patients. **b** Lower H3K27me3 protein expression in BM derived AML samples compared to normal BM (NBM, *n* = 10) and BM derived ALL samples. **c** Heatmap showing histone methylation levels in peripheral blood (PB) samples from AML (*n* = 79) and ALL (*n* = 48) patients. **d** Lower H3K27me3 protein expression in PB derived AML samples compared to NBM and PB derived ALL samples. ****p < 0.0001, ns = not significant. **e** Heatmap combining AML and ALL samples from both sources along with cytogenetic information, presence of FLT3 or NPM1 mutation and age group

Global histone modification expressions were analyzed using RPPA in freshly prepared protein samples and normalized relative to the mean expression of healthy CD34+ controls (*n* = 10). H3K4me2, H3K4me2 and H3K27me3 levels were then studied in BM and PB separately. First, we observed that all three methylation marks were less highly expressed in leukemic BM and PB than in non-malignant CD34+. To compare expression of the marks between AML and ALL we performed hierarchical clustering and this led us to the identification of 4 clusters on the basis of lineage (myeloid vs. lymphoid) in the 241 BM-derived cases (ALL = 79 and AML = 162, Fig. 1a). From left to right, the first cluster with highest expression of the histone methylation marks, was comprised predominantly of ALL patients as well as the majority of the second cluster. In contrast, both clusters on the right with lower expression of the marks were enriched by AML BM-derived cases, with no ALL cases found in the fourth cluster. Histone methylation mark H3K27me3 showed the most variable expression across the sample set and had significantly lower expression in AML patients compared to those with ALL and healthy controls (Fig. 1b, *p* < 0.0001). In the 127 PB samples (ALL = 48, AML = 79, Fig. 1c) four clusters were found according to cell lineage with repeatedly higher expression of H3K27me3, H3K4me2 and H3K4me3 in the ALL-enriched cluster. The residual clusters with lower expressions were composed of PB samples from AML patients. H3K27me3 again distinguished AML PB patients from normal BM and ALL PB cases (Fig. 1d, *p* < 0.0001). Histone methylation levels did not significantly differ between BM and PB in AML (H3K27me3; *p* = 0.36, H3K4me2; *p* = 0.37, H3K4me3; *p* = 0.11, data not shown), thus samples from both sources were combined for subsequent data analysis A combined heatmap with BM and PB of both AML and ALL was created along with patient features including disease lineage, age group (< 65 or > 65 years), cytogenetics, FLT3 and NPM1 mutation (Fig. 1e). Unsupervised hierarchical clustering of the histone methylation marks separated ALL from AML cases regardless of source, but no associations with mutations and cytogenetics were observed. A total of 16 ALL patients fell into cluster 1 and 2 that was highly enriched for AML patients and 12 AML fell in the third ALL-enriched cluster. However, we did not observe a common (cyto)genetic feature that could characterize these patients. H3K27me3, H3K4me2 and H3K4me3 levels according to patient and disease characteristics is provided in Additional file 5: Table S1 (AML) and Additional file 6: Table S2 (ALL).

### Histone methylation marks correlate with clinical features and outcome in AML

Here, we report the clinical implications of expression levels of these histone methylation marks in acute leukemia. In AML, no significant differences in methylation marks were seen according to sex, performance status, or the presence of an infection at the time of sampling. H3K27me3 was lower in AML patients with antecedent hematological disorder (AHD, *p* = 0.035, Additional file 4: Figure S4) than without. Significant H3K27me3 and H3K4me2 variation was observed among categories of the French-American-British (FAB) classification based on morphology with lower abundance of H3K27me3 in M5 and M6, H3K4me2 in M4 and M5 classified AML, and higher levels of H3K27me3 in M1, M2 and M0 and H3K4me2 in RAEBT, CMML-T and M0 subtypes (H3K27me3; *p* = 0.0013, H3K4me2; *p* = 0.014, Additional file 1: Figure S1). The protein levels of all histone methylation marks correlated negatively with the percentage of BM and PB monocytes, platelet count and CD33. A positive correlation between CD13 and H3K4 methylation marks was observed, as well as between CD34 and H3K4 methylation marks. H3K27me3 further correlated negatively with serum creatinine and age and positively with CD7 and CD19 (Additional file 2: Figure S2−Additional file 4: Figure S4). Prior malignancy per se was not an indicator of differences in histone methylation marks, but patients with prior exposure to chemotherapy (*n* = 23) or radiotherapy (*n* = 21) had higher H3K4me3 (*p* = 0.019 and *p* = 0.043, Additional file 3: Figure S3) and H3K4me2 *(p* = 0.053, Additional file 2: Figure S2). Additionally, neither individual cytogenetic events, nor broad prognostic cytogenetic prognostic groups (‘favorable’,’intermediate’, ‘unfavorable’, Additional file 1: Figure S1) correlated with methylation marks. Among individual recurrent mutations; we observed similar levels of the marks in fms-like tyrosine kinase-3 internal tandem duplication (FLT3-ITD) mutations and FLT3 wildtype, but lower H3K27me3, H3K4me2 and H3K4me3 was observed in patients with the FLT3 tyrosine kinase domain (FLT3-D835) mutation (*n* = 19) compared to those without (*p* = 0.068; *p* = 0.014; *p* = 0.029, respectively, Additional file 2: Figure S2–Additional file 4: Figure S4). We found no histone methylation differences among the other most common mutational findings typical of AML, regardless of whether they are associated with a functional change in DNA methylation regulating genes (TET2, IDH1/2, DNMT3A) or independent of methylation function (NPM1, TP53, PTPN11, Additional file 5: Table S1).

**Fig. 2:**
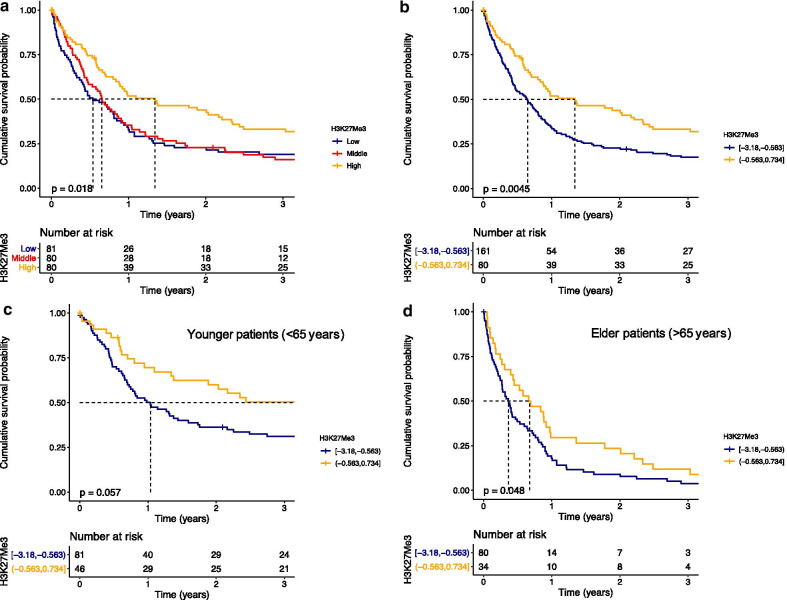
Overall survival (OS) in **a** AML patients (*n* = 241) stratified in three equally divided patient groups based on H3K27me3, **b** AML patients (*n* = 241) stratified in two groups, **c** younger AML patients (< 65 years, *n* = 127) and **d** AML patients older than 65 years (*n* = 114) according to H3K27me3 low (blue) and high (orange) status

To investigate the effects of H3K27me3 on outcome in our AML population, we equally divided patients into three groups based on H3K27me3 levels. We observed that patients with highest H3K27me3 (H3K27me3^high^) had better outcomes than the overlapping low and middle groups (*p* = 0.018, Fig. 2a). When patients in H3K27me3^high^ (*n* = 80) were compared to the combined lower and middle third groups (H3K27me3^low^, *n* = 161), they had better survival (median OS 16.1 vs. 7.8 months, HR = 0.64, 95% CI = 0.47–0.87, *p* = 0.0045) (Fig. 2b). Outcome in elderly patients with AML is consistently inferior to that in younger patients (< 65 years) [19]. We observed that levels of H3K27me3 tend to decrease with age (Additional file 4: Figure S4), suggesting that this could be a biological difference between the age groups. We therefore tested whether H3K27me3 levels were prognostic across age groups. We observed similar trends as higher levels of H3K27me3 was favorable in both the younger (< 65 years*,* HR = 0.64, 95% CI = 0.40–1.02, *p* = 0.057, Fig. 2c) and the elderly (> 65 years, HR = 0.66, 95% CI = 0.43–0.99, *p* = 0.048, Fig. 2d). This indicates that H3K27me3 level is prognostic across age groups.

### Loss of H3K27me3 is an independent poor prognostic factor in AML

**Fig. 3:**
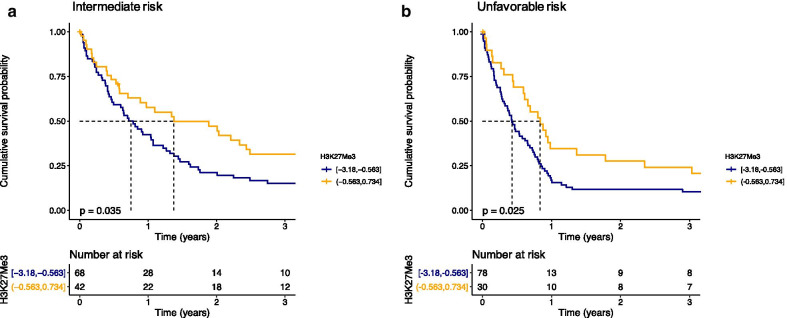
Overall survival (OS) in **a** intermediate risk AML patients (*n* = 110) and **b** unfavorable risk AML patients (*n* = 108) according to H3K27me3 low (blue) and high (orange) status

Inferior survival experiences were also observed among patients with H3K27me3^low^ in both the intermediate (HR = 0.62, 95% CI = 0.40–0.97, *p* = 0.035, median OS of 9 months vs. 16.5 months, Fig. 3a) and unfavorable (HR = 0.60, 95% CI = 0.38–0.94, *p* = 0.025, median OS of 5.1 months vs. 10 months, Fig. 3b) cytogenetics. H3K27me3 levels failed to differentiate patients with distinct OS in the favorable (median survival not reached, *p* = 0.99) cytogenetic risk subgroup (data not shown).

Risk stratification within the WHO subgroups is heavily influenced by the mutations present in those cases. CEBPA and NPM1 mutations are often referred to as favorable in the absence of poor prognostic mutations. DNMT3A, RAS, IDH1 and IDH2 are often present in the intermediate risk group and the presence of FLT3 is a poor prognostic factor characterizing unfavorable grouped patients. H3K27me3 thus predicts outcome in the intermediate risk group, including CN-AML, and the unfavorable prognosis cytogenetic group. We then hypothesized that the prognostic influence of H3K27me3 was independent of mutations and cytogenetic subgroup. To study the prognostic impact of H3K27me3 in the context of cytogenetic aberrations and mutations, Cox regression analysis was performed. We included cytogenetic aberrations, FLT3-ITD and D835, NPM1, DNMT3A, IDH1, IDH2, TET2, TP53 and H3K27me3 level on a continuous scale. In univariate analysis, several cytogenetic categories (i.e. trisomy 21, diploid, inv(16), t(8;21) and miscellaneous AML) were individual prognostic factors (Table 1), but not the recurrent driver mutations. Of our cohort, 54 patients were tested for a TP53 mutation during diagnostic practice with 8 being positive (15%) and this mutation was a significant contributor to poor prognosis in the univariate analysis. The H3K27me3 expression level was a significant predictor for better outcome (HR = 0.74, 95% CI = 0.58–0.94, *p* = 0.01, Table 1).

**Table 1:** Univariate and multivariate cox regression analysis of acute myeloid leukemia patients (*n* = 241)

Variable	Univariate analysis			Multivariate analysis		
	HR	95% CI	*P* value	HR	95% CI	*P* value
Cytogenetic category						
chr –5, –7	0.69	0.21–2.30	0.54			
chr –5, –7, + 8	0.54	0.12–2.44	0.43			
chr –7	0.63	0.21–1.93	0.42			
11q23	0.32	0.10–1.08	0.07			
chr + 21	0.09	0.02–0.51	0.01	0.09	0.02–0.51	0.01
chr 5q–	0.94	0.10–8.43	0.95			
chr 7q–	2.92	0.32–26.83	0.34			
chr + 8	0.36	0.12–1.12	0.08			
Diploid	0.24	0.09–0.66	0.01	0.23	0.08–0.64	< 0.01
Inv(9)	0.64	0.12–3.53	0.61			
ssInv(16)	0.05	0.01–0.20	0.00	0.05	0.01–0.18	< 0.01
Miscellaneous	0.30	0.11–0.85	0.02	0.30	0.11–0.84	0.02
t(6;9)	0.32	0.08–1.29	0.11			
t(8;21)	0.06	0.01–0.27	0.00	0.06	0.01–0.27	< 0.01
FLT3						
ITD	1.22	0.86–1.73	0.26			
D835	0.75	0.43–1.29	0.30			
Any	1.21	0.86–1.69	0.28			
NPM1	1.07	0.71–1.61	0.74			
DNMT3A	0.94	0.46–1.93	0.87			
IDH						
IDH1	0.76	0.43–1.35	0.35			
IDH2	1.50	0.88–2.56	0.13			
Any	1.03	0.67–1.58	0.91			
P53	2.50	1.14–5.55	0.02			
RAS	0.68	0.45–1.04	0.07			
PTPN11	0.53	0.21–1.32	0.17			
TET2	1.62	0.89–2.87	0.11			
H3K27Me3						
Continuous	0.74	0.58–0.94	0.01	0.74	0.57–0.95	0.02

Multivariate analysis was performed with and without TP53 as covariate since analysis was restricted to only 54 patients when TP53 status was included. H3K27me3 level was an independent favorable prognostic factor in the whole population (*n* = 241, HR = 0.74, 95% CI = 0.57–0.95, *p* = 0.02, Table 2) and in the known TP53 status subgroup (*n* = 54, HR = 0.48, 95% CI = 0.26–0.87, *p* = 0.02). In both sets, diploid, inv(16) and miscellaneous AML were independent prognostic factors. When TP53 was excluded as covariate, trisomy 21 and t(8;21) were also significant independent prognostic contributors (Table 1).

**Table 2:** Multivariate cox regression analysis of acute myeloid leukemia patients (*n* = 65)

Variable	Multivariate analysis		
	HR	95% CI	*P* value
H3K27Me3 (continuous)	0.49	0.25–0.95	0.03
BCOR	1.67	0.70–4.03	0.25
ASXL1	0.59	0.24–1.45	0.25
U2AF1	0.70	0.16–2.97	0.63
SRSF2	0.57	0.23–1.41	0.22
Presence of any of these mutations	2.36	0.85–6.57	0.10

**Fig. 4:**
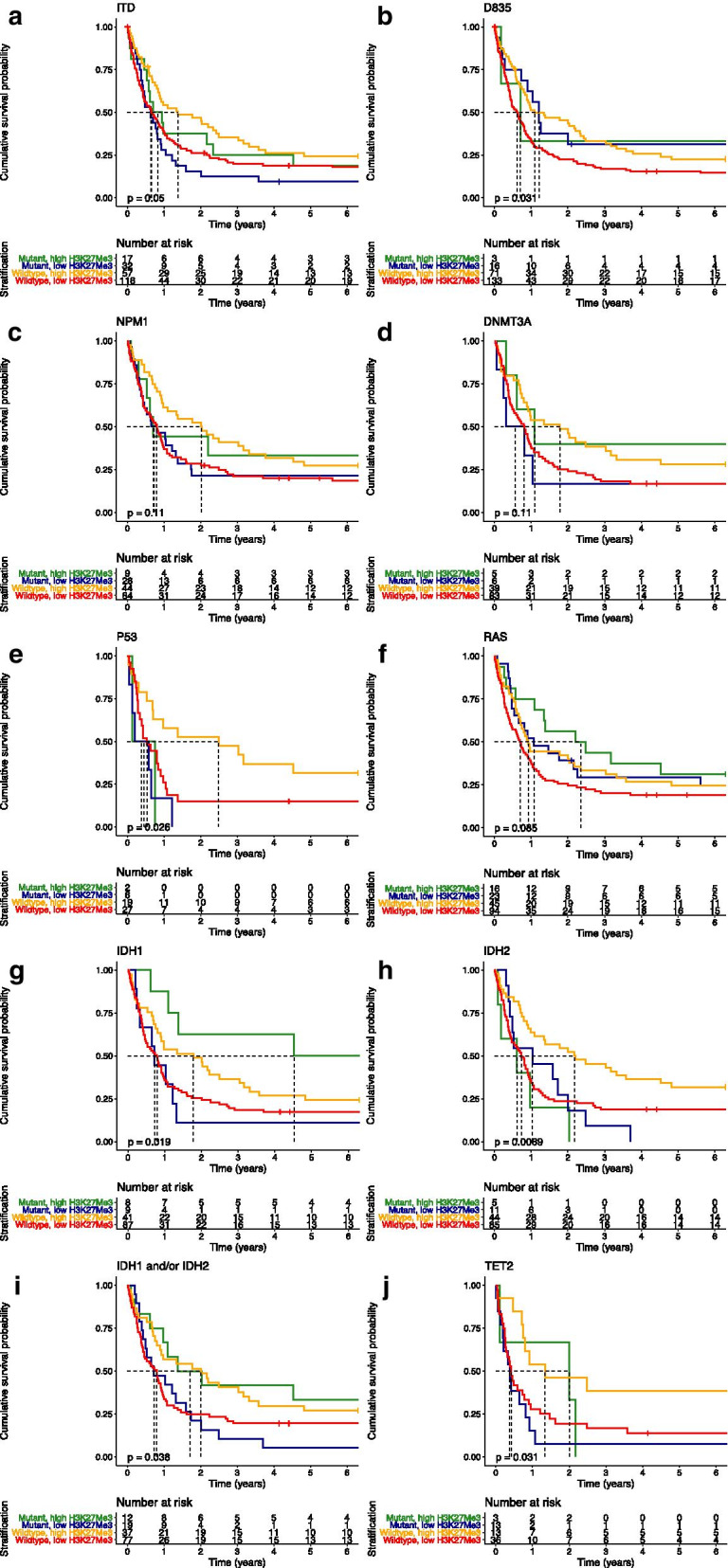
Overall survival (OS) in AML patients according to H3K27me3 low and high and individual mutational status; **a** FLT3-ITD; **b** FLT3-D835; **c** NPM1; **d** DNMT3A; **e T**P53; **f** RAS; **g** IDH1; **h** IDH2; **i** IDH1 and/or IDH2; **j** TET2

To test whether H3K27me3 could contribute to risk stratification in mutated patients despite cytogenetic risk group, we compared OS between patients with H3K27me3^low^ and H3K27me3^high^ in FLT3-ITD, FLT3-D835, NPM1, DNMT3A, TP53, RAS, IDH1, IDH2, TET2 wildtype and mutated AML (Fig. 4). Loss of H3K27me3 predicted poor prognosis among all wildtype populations, but was most obvious in the TP53 wildtype population. TP53 mutation led to an unfavorable OS rate in spite of H3K27me3 levels, but TP53 wildtype patients did as bad as the mutated population when patients were in the H3K27me3^low^ group (median OS 6.4 vs. 29.8 months, *p* = 0.049, Fig. 4e). H3K27me3 was not prognostic in individual mutated subgroups except for IDH1. In general, response rate and OS are comparable between AML patients with IDH1 and IDH2 mutations and IDH wildtype [20]. Here, we found that higher H3K27me3 increased OS only in the IDH1 mutated population compared to low H3K27me3 (median OS of 8.7 vs. 54.3 months, *p* = 0.0075, Fig. 4g). When IDH1 and IDH2 data were combined, we observed the same tendency in the mutated cases (*n* = 31, median OS 8.7 vs. 20.4 months, *p* = 0.026) as in the wildtype group (*n* = 114, median OS 9.6 vs. 24.1 months, *p* = 0.042, Fig. 4i). In the IDH2 mutated population we found no significant differences in terms of OS on the basis of H3K27me3. No significant differences in remission duration and event-free survival were observed (data not shown).

**Fig. 5:**
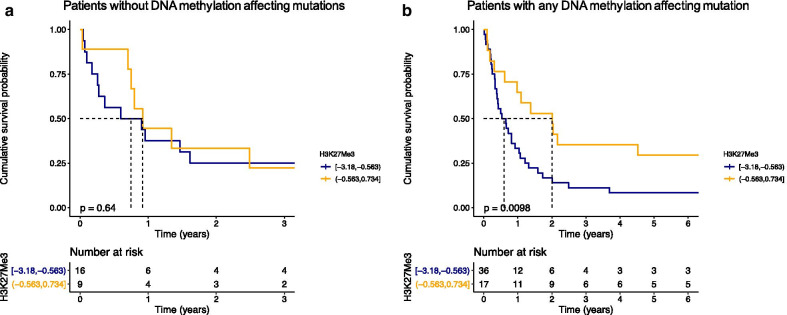
Overall survival (OS) in AML patients according to H3K27me3 low (blue) and high (orange) in **a** patients without any DNA methylation affecting mutation (i.e. IDH1, IDH2, DNMT3A and TET2, *n* = 25) and **b** in patients with a mutation in at least one DNA methylation affecting gene (*n* = 53)

Besides IDH mutations, DNMT3A and TET2 are also major methylation affecting mutations. A total of 78 AML patients had molecular data available for these four genes (wildtype or mutation). We then separated patients in two groups; no DNA methylation mutation (*n* = 25) or any DNA methylation mutation present (*n* = 53) and compared OS between H3K27me3^low^ and H3K27me3^high^ in both groups. H3K27me3 was not prognostic in the patients who had none of these mutations (Fig. 5a). In contrast, patients with at least one mutation in any DNA methylation gene had significant improved OS when H3K27me3 levels were high compared to those in the H3K27me3^low^ group (median OS 7.1 vs. 24.1 months, HR = 0.42, 95% CI = 0.21–0.83, *p* = 0.01, Fig. 5b).

### Influence of the mutational profile in relation to H3K27me3 in AML

**Fig. 6:**
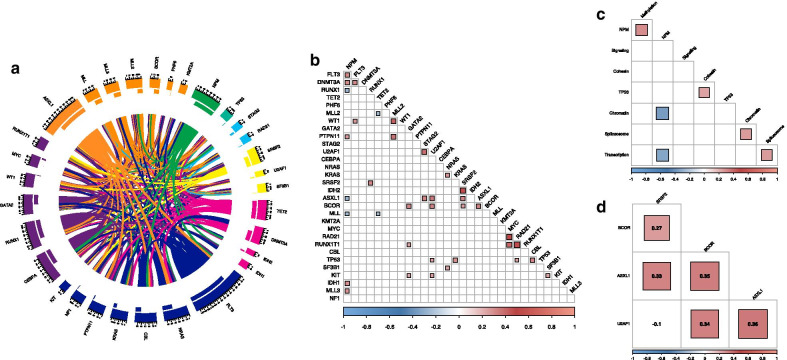
**a** Circos plot including driver mutation associations among 65 AML patients. Colors are determined by functional pathway of each given gene. **b** Patterns of association of mutations among studied patients. MLL mutations also include MLL-MLLT3 and MLL-MLLT4 fusion gene lesions while KMT2A represents KMT2A specific gene lesions only. **c** Patterns of association of mutational pathway abnormalities among studied patients. **d** Patterns of association of mutations among ASXL1, BCOR, U2AF1, SRSF2 patients with significantly lower H3K27me3 levels than wildtype. Filled areas represent significant positive (red) or negative (blue) co-occurrence *p* < 0.05. Color and size indicate of filled area indicates Pearson’s correlation coefficient

Although H3K27me3 level was prognostic in IDH1 mutated AML and overall in patients with at least one DNA methylation affecting mutation, mutations in genes encoding for epigenetic modifiers often co-occur with each other and other major AML mutations. We therefore aimed to determine histone methylation mark expression in the light of a broader mutational landscape of our AML patient cohort and their response to therapy. To explore this, we analyzed sequencing data available for 65 BM aspirates of our AML cohort. A total of 788 mutations were detected in 271 genes. The median number of mutations in each patient was 12 (range [6–22]). Among the 271 genes, a total of 31 AML driver mutations acting in 8 different molecular pathways were found in 100% of the patients. Details of the driver mutations and molecular pathways are described in the Additional file 15. The majority of patients had more than one mutation in the same molecular pathway including 8 (12%) cases within 2 DNA methylation genes, 16 (25%) in 2–4 chromatin regulating genes, 13 (20%) with 2–3 mutations in transcriptional regulators and 15 patients (23%) with 2–3 mutations in the RTK-RAS signaling pathway (Fig. 6a).

To study whether chromatin modifying mutational events tend to co-occur with other mutations, we evaluated patterns of occurrence of genomic alternations in the 65 cases. A total of 37 significant pairwise associations between driver mutations were observed (Fig. 6b, Additional file 7: Table S3). In most of these genomic pairing associations, NPM1, chromatin regulating and spliceosome mutations were involved (Fig. 6c, Additional file 8: Table S4). Mutational events in the chromatin regulating pathway were rare in the presence of NPM1 mutations (*r* =  − 0.35, *p* = 0.004), but positively associated with mutations in the spliceosome (*r* = 0.26, *p* = 0.036) with ASXL1 co-occurring with both SRSF2 (*r* = 0.33, *p* = 0.008) and U2AF1 (*r* = 0.36, *p* = 0.003). Mutations that affect DNA methylation significantly co-occurred with NPM1 (*r* = 0.30, *p* = 0.014). Spliceosome affecting mutations associated with those that regulate transcription (*r* = 0.26, *p* = 0.033) with significant co-occurrence between SRSF2 and RUNX1 mutations (*r* = 0.33, *p* = 0.008).

Next, to examine whether loss of H3K27me3 associated with epigenetic modifying mutations, we determined the levels of H3K27me3 per mutational subgroup and found that levels were significantly lower in ASXL1, BCOR, U2AF1 and SRSF2 mutated subgroups (Additional file 9: Figure S5). A total of 26 patients had at least one of these mutations which highly significantly associated with each other (Fig. 6d). Twenty-four of these 26 (92%) were in the H3K27me3^low^ group, compared to 26 of the 39 (67%) patients with no ASXL1, BCOR, U2AF1 or SRSF2 mutation (Chi-Square test, *p* = 0.016). We next sought to determine whether these mutations and/or H3K27me3 had prognostic value. We therefore conducted multivariate cox regression analysis of the 65 patients with sequence data available using the following covariates: ASXL1, BCOR, U2AF1, SRSF2, the presence of any of these mutations and continuous H3K27me3 level. This confirmed significantly better OS in patients with higher H3K27me3 levels (HR = 0.49, 95% CI = 0.25–0.95, *p* = 0.04), regardless of mutational status (Table 2).

### Protein expression profiling associated with H3K27me3 loss identifies an anti-apoptosis related phenotype in AML

H3K27me3 is of prognostic significance in our AML cohort with worse outcomes overall and in particular subgroups, i.e. intermediate and unfavorable AML, IDH1, ASXL1, BCOR, U2AF1 and SRSF2 mutated patients. To recognize dysregulated pathways in patients with increased loss of H3K27me3 in AML, we examined correlations of H3K27me3 with the other 229 proteins on the AML719 array. H3K27me3 is catalyzed by the polycomb group protein EZH2 and is linked to transcriptional repression via the formation of heterochromatin regions [21]. To identify upregulated proteins and pathways upon the loss of H3K27me3, we specifically focused on significant (*p* < 0.0001) negatively correlated proteins with H3K27me3 with Pearson’s correlation coefficient *R* > 0.25. This led us to the identification of 20 total and 6 phospho-proteins that showed increased expression upon decreased H3K27me3 (Additional file 10: Figure S6). Protein network analysis was performed on the negatively associated set of proteins with H3K27me3 using the String software (String 10.1, http://string-db.org). The upregulated protein network upon H3K27me3 loss as shown in Additional file 10: Figure S6 was highly associated with a protein/protein interaction enrichment *P* value of < 1.0e−16. Functional enrichment analysis identified 763 significantly enriched biological processes. The top enriched processes include *negative regulation of programmed cell death* (GO: 0043069) and *negative regulation of apoptotic processes* (GO: 0043066). The proteins that contributed to the negative regulation of programmed cell death or apoptosis include PRKCA, PRKCD, RPS6KB1, PIK3CA, PRKAA1, MET, CDKN1A, SFN, AKT1, SRC, BID, MCL1, ERBB3, NRP1, SMAD6, PTGS2, NOL3 and YAP1 (Additional file 10: Figure S6). All other corresponding processes are listed in Additional file 11: Table S5.

### Expression levels of H3K27me3, H3K4me2 and H3K4me3 associate with clinical features in ALL

In ALL, H3K27me3, H3K4me2 and H3K4me3 levels were higher in the BM-derived leukemic blasts samples compared to those in PB samples (Additional file 12: Figure S7–Additional file 14: Figure S9). We, therefore, evaluated the clinical impact of the histone methylation marks per source. The methylation marks were not different on the basis of gender, AHD, response after induction therapy, relapse, vital status, FAB classification, cytogenetic subtype or lineage. All marks measured in PB correlated positively with the percentage of peripheral blasts and bone marrow blasts. H3K4me3 and H3K27me3 in PB further correlated positively with white blood count (WBC) and absolute blast count (Additional file 12: Figure S7–Additional file 14: Figure S9). WBC also correlated positively with H3K4me3 in BM (*r* = 0.25, *p* = 0.025). Lower H3K27me3 was seen in BM-derived samples from patients that had an infectious complication (*p* = 0.005) and therefore experienced an event (*p* = 0.019, Additional file 14: Figure S9). BM levels of H3K4me2 were lower in patients who were diagnosed with a prior malignancy (*p* = 0.045, Additional file 12: Figure S7) than those who were not. A positive correlation between H3K4me2 levels in BM and CD7 expression (*r* = 0.25, *p* = 0.031) was found and a negative correlation with human leukocyte antigen (HLA) expression (*r* =  − 0.28, *p* = 0.013). CD13 expression negatively correlated with H3K27me3 levels in BM samples (*r* =  − 0.28, *p* = 0.014). H3K4me3 levels were higher in samples with higher albumin (*r* = 0.31, *p* = 0.036) (Additional file 13: Figure S8). Individual histone methylation mark expression in ALL derived samples did not correlate with OS or remission duration and therefore are not shown.

### Histone methylation marks correlate with response to tyrosine kinase inhibition in ALL

**Fig. 7:**
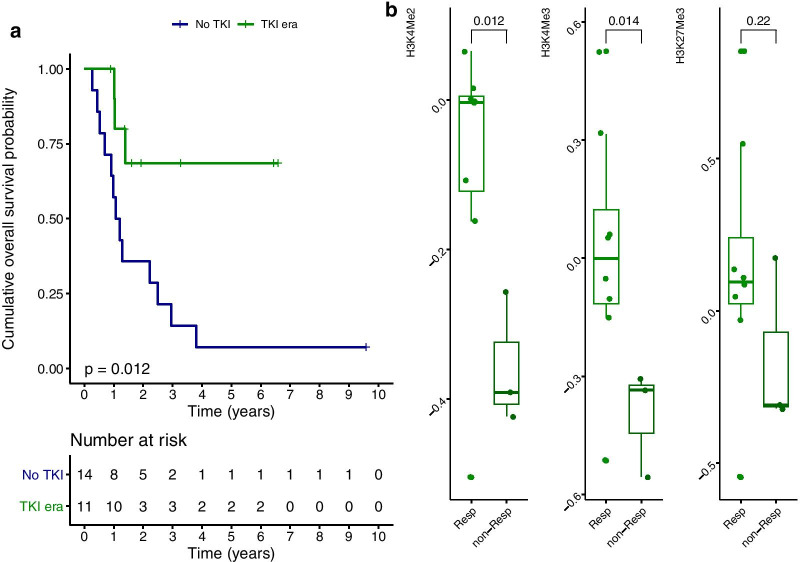
**a** Overall survival (OS) between Ph+ ALL patients treated with TKIs (green) and those who were diagnosed with Ph+ ALL before TKI implementation (blue). **b** Relative RPPA levels of H3K4me2 (left), H3K4me3 (middle) and H3K27me3 (right) of TKI treated Ph+ ALL patients (*n* = 11) according to 5-years survivors (Resp = responder alive after 5-years follow-up, non-Resp = non-responder who passed away within 5 years of follow-up). Significant lower H3K4me2 and H3K4me3 was observed in non-responders compared to responders

In 20–30% of adults with ALL, the Philadelphia cytogenetic aberration is present (Ph+) leading to the production of the *BCR-ABL* fusion gene. Ph+ ALL historically associated with poor prognosis but outcomes have improved substantially with the use of TKI targeting *BCR-ABL* [22]. However, prognosis remain poor as relapse frequently occur with resistance against TKI. Mechanisms underlying TKI resistance include mutations targeting transcriptional and epigenetic regulation [23, 24]. In our cohort, 11 of 25 Ph+ ALL patients were treated with TKIs and we asked ourselves whether an expression profile of histone methylation marks relates to TKI response. As expected, only three patients (3/11, 27%) died after TKI treatment compared to 93% (13/14) of Ph+ ALL patients who were not treated with TKIs (*p* = 0.012, Fig. 7a). We observed a positive relation between higher H3K4me2 (*p* = 0.012) and H3K4me3 (*p* = 0.014) at time of diagnosis and long-term response (5-years OS) after TKI treatment (Fig. 7b). The same association between H3K27me3 and response was observed, but significance was not reached (*p* = 0.22). Noteworthy is that TKI resistance has been proposed to associate with smoking in Ph+ ALL due to altered DNA methylation. Retrospectively, we identified that 2 of 11 TKI-treated patients were smokers. Both patients presented with highest loss of the histone methylation marks at diagnosis, were resistant against TKIs and died after 1 year. Thus 2 out of 3 patients (66.7%) Ph+ ALL patients with resistance against TKI (non-responders) were smokers compared to none of the 8 responders that were alive after > 5 years of follow-up.

## Discussion

Prognostication in acute leukemia is currently guided by cytogenetic and molecular abnormalities, however, these often lack the ability to accurately predict treatment responses since they occur in overlapping patterns of co-occurring events. In this study, we found that proteomic profiling of epigenetic modifications of histones improves outcome prediction and has clinical implications. This supports the idea that epigenetic patterns contribute to a more accurate picture of the leukemic state than morphology, flow cytometry and mutation analysis alone. This is the first study that focuses on global histone methylation marks in a large cohort of adult-onset AML and ALL patients, correlating histone methylation with the expression of other proteins and mutational analysis. Among the analyzed histone modifications is H3K27me3 that is associated with transcriptional downregulation via the formation of heterochromatin. Loss of H3K27me3 has been reported in a variety of cancers as a poor prognostic factor. In our study we observed higher loss of H3K27me3 in AML than in ALL, and that was prognostic in the presence of a methylation regulating mutation (i.e. DNMT3A, IDH1-2 and/or TET2), but not without. Di- and trimethylation of H3K4 on histone tails is related to open chromatin and active transcription and had no prognostic significance in AML—but relatively low levels of these marks were found in the Ph + -ALL patients of our cohort who were resistant to TKI-inhibitors.

**Fig. 8:**
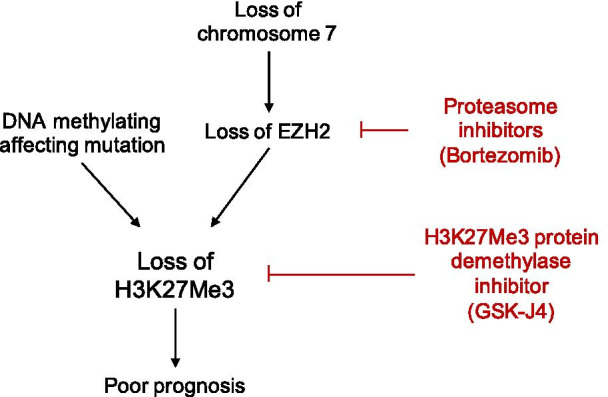
Schematic representation of hypothesis based on the loss of H3K27me3 in AML

In AML, del(7) or del(7q) with subsequent loss of EZH2 (located on chr.7q36.1) decreased H3K27me3 expression has been reported [25]. AML with del(7) or del(7q) is generally associated with poor prognosis that might be partially be caused by the loss of EZH2 and H3K27me3 since this was linked to chemoresistance [25, 26]. Interestingly, based on cytogenetics, level of H3K27me3 was lower in AML patients with del(7) or del(7q) compared to patients without chromosome 7 abnormalities in our cohort (Additional file 1: Figure S1). In line with this, we report that greater loss of H3K27me3 in AML was an independent factor for worse patient outcome, regardless of cytogenetic subgroup or mutations. Inhibiting H3K27 by methyltransferases inhibitors contributed to chemoresistance in both in vitro and in vivo xenograft mouse AML models which was rescued by EZH2 restoration using proteasome inhibitors (PI) [25]. This might provide a rationale for the addition of PI in the AML treatment regime for identified patient subgroups with H3K27me3 loss and poor outcomes, i.e. intermediate and unfavorable AML, IDH1, ASXL1, SRSF2, U2AF1 and BCOR mutated patients. Another approach that may lead to an increase in H3K27me3 levels is by the inhibition of H3K27me3 demethylators such as KDM6B. This protein is upregulated in AML and its expression is associated with global reduced H3K27me3 levels. Pharmacological inhibition of KDM6 with GSK-J4 reduces AML cell survival and is under consideration as treatment option in AML [27, 28]. Here, we propose that GSK-J4 may have the largest effect in the aforementioned patients with loss of H3K27me3 and poor outcomes including those with del(7) or del(7q) and any DNA methylation affection mutation (Fig. 8).

Mutations in U2AF1 and SRSF2 affect the spliceosome and are frequently found in antecedent hematological disorders, such as myelodysplastic syndrome (MDS) and myeloproliferative neoplasms (MPN), as well as are mutations in chromatin regulating genes ASXL1 and BCOR. The global loss of H3K27me3 in patients with these mutations corresponded with our finding of lower levels of H3K27me3 in patients with AHD; 54% of the AML patients with ASXL1, BCOR, U2AF1 and/or SRSF2 were diagnosed with AHD compared to only 18% of the patients without these (*p* < 0.01). BCOR, SRSF2, U2AF1 and ASXL1 mutations all confer poor prognosis in myeloid malignancies [29–37]. Here we report that the prognostic influence of these mutations is at least to some extent dependent on the degree of loss of H3K27me3—an independent adverse risk factor for survival in our sequenced cohort. Global level of H3K27me3 depends on numerous processes and although mutations in chromatin regulating genes are one important regulator, other mechanisms and pathways are likely involved. If H3K27me3 is restored, gene transcription profiles are less likely to be affected by these mutated genes. For example, ASXL1 regulates trimethylation of H3K27 via interaction with polycomb repressive complex 2 (PRC2) proteins, but if mutated, loss-of-function leads to loss of H3K27me3 without changing PRC2 expression and activity [38, 39]. Although mutations in chromatin regulating genes are predictive for response to therapy, we suggest that additional measurement of H3K27me3 in AML can lead to a more precise outcome prediction.

The advantage of proteomic analysis by RPPA is the simultaneous analysis of key components of the proteome along with a particular protein of interest. We found here that H3K27me3 negatively correlated with a subset of proteins that associated with an anti-apoptotic phenotype. This phenotype is likely to contribute to the poor prognostic character of AML with H3K27me3 loss. In osteosarcoma cancer, decreased H3K27me3 expression led to chemotherapy resistance via PRKCA and MCL1 participation [40]. These were among the strongest negatively correlated proteins with H3K27me3 in our cohort suggesting a similar mechanism at work in AML. PRKCA is known to exert anti-apoptotic functions and PRKC inhibition in AML suppresses BCL2 phosphorylation and thereby initiates apoptosis [41]. MCL1 has also been described as a therapeutic target in AML. It is often upregulated as AML cells are dependent on the anti-apoptotic actions of MCL1 for maintenance of survival. Worth mentioning is that resistance against the BCL2-inhibitor venetoclax in AML is often caused by MCL1 overexpression [2, 42–44].

Besides a negative correlation between H3K4 methylation marks and percentage of BM and PB monocytes, our study did not support a role for H3K4 methylation in outcome predictions in AML. An interesting finding however is that both H3K4me2 and H3K4me3 were higher in samples from patients who received prior chemotherapy and also higher H3K4me3 in patients who underwent previous radiotherapy. Drug-induced DNA hypermethylation has been identified in a variety of cancers [45], but we are the first to report higher levels of H3K4 in therapy-related AML. Although RPPA measures accurate quantities of histone methylation in subgroup of patients, it is the histone methylation mark distribution that directs chromatin modification and transcriptional programs; thus, other studies are needed to investigate the consequences of drug-induced H3K4 deposition on chromatin in relation to therapy-related AML.

In ALL, we observed higher expression of H3K27me3, H3K4me2 and H3K4me3 in BM derived lymphoid blasts compared to its expression in peripheral blasts. Global mapping of H3K4me3 and H3K27me3 in lymphocytes have revealed that the amount of H3K4me3 and H3K27me3 across the gene body correlate with specific gene expression profiles and that these reflect differentiation states [46]. This phenomenon might provide one explanation why we observed different quantities of histone methylation between lymphoblasts originating from BM or the PB. No differences based on the amount of H3K27me3, H3K4me2 and H3K4me3 among lineage (T or B-cell) entities were seen. An interesting finding is that Ph+ ALL with resistance against TKIs showed significantly reduced levels of methylation marks at diagnosis compared to long-term survivors. Patient number is very limited, but it is noteworthy that two out of three patients who were resistant against TKI were prior smokers compared to none of the 8 responders. In patients with non-small cell lung cancer, smokers have been identified with lower H3K27me3 protein expression compared to non-smokers [47]. Furthermore, cigarette smoke has already been proposed to accelerate progression of FLT3-ITD AML by altering DNA methylation [48]. The concept that smoking alters the epigenetic machinery and associates with response to TKI in Ph+ ALL thus warrants further investigation.

Emerging studies have already shed light on a role for epigenetic dysregulation and classification in both ALL and AML. For example, methylation profiling of CpG sites can predict survival in each subtype [49], as well as can be used to distinguish lymphoid from myeloid cases [49, 50]. We report here that, besides CpG methylation, global histone modification is also differentially expressed between lymphoid and myeloid leukemia and measurement at time of diagnosis correlates with response to therapy.

## Conclusions

Our data supports that proteomic profiling of histone methylation levels improves outcome prediction in AML and Ph+ ALL and suggest potential targets to further investigate diagnostically and therapeutically. H3K27me3 loss associates with tumor aggressiveness and patient outcomes in numerous cancers [10–16], similar as we found in AML.

## Methods

### Sample collection

BM and PB samples were collected during routine diagnostic assessments from 113 newly diagnosed B-ALL, 14T-ALL, and 241 AML adult patients who were admitted at the MD Anderson Cancer Center (MDACC) between September 1991 and March 2007. Informed consent was obtained and conducted in accordance with the Declaration of Helsinki. Collection and analysis of samples was in accordance with protocols (Lab 01-473 and Lab 05-0654 respectively) approved by the MDACC Investigational Review Board (IRB). The analysis of outcomes within this study was restricted to newly diagnosed patient samples. Clinical data was available for all 127 ALL and 241 AML patients.

### Sample preparation

After collection, samples were immediately placed on ice and were processed within 2 h of collection. The AML patient samples underwent CD3 and CD19 depletion to remove contaminating T and B cells if they were deemed to represent > 5% of cells based on the differential. No additional purification after Ficoll separation was performed on ALL samples as samples contained high blast percentage. After cells were normalized to a 1 × 10^4^ /mL concentration, whole-cell lysates were prepared as described earlier [51]. In short, 10 million leukemia blast-enriched cells were suspended in 500 μL PBS, then lysed by adding 500 μL 2X boiling hot protein lysis buffer (Tris buffered saline pH 7.4, with 10% SDS and 2% beta-mercaptoethanol). After boiling for 2–3 min and vortexing, samples were aliquoted and cryopreserved until use [11].

### Reverse phase protein array

Proteomic profiling was performed on AML and ALL sample preparations using RPPA (AML719ALL360 array). Methodology as well as validation of technique and the 230 antibodies used have been fully described in prior publications [52–54]. Briefly, protein cell lysates were diluted in five serial dilutions in 96-well plates and transferred into 384-well plates. The diluted lysates were then printed onto nitrocellulose-coated glass slides. Material was deposited by direct pin contact with a single touch per dot. This printed approximately 85, 42, 21, 11, and 5 cell equivalents of protein in the five dots for each sample. As controls, the same protein lysis buffer used to make the protein preps (Biorad Lamelli buffer) was used as a negative control and a protein prep prepared from a mixture of 11 different AML cell lines was made as a positive control [55]. On each array, we included BM derived CD34+ samples from ten healthy individuals as controls. After slide printing, all patient and control samples were simultaneously probed with one strictly validated primary antibody per slide, a secondary antibody to amplify the signal, and a stable dye as detection agent. The AML719ALL360 array allowed us to simultaneously measure expressions of 230 highly validated protein antibodies including 3 Histone 3 (H3) post-translational methylation modifications; lysine 4 dimethylation (H3K4me2), trimethylation, (H3K4me3) and lysine 27 trimethylation (H3K27me3). A complete list of the antibodies validated for RPPA by our lab was published by Hu et al. [54]. Stained slides were analyzed using Microvigene software (Version 3.4, Vigene Tech, Carlisle, MA).

### Sequencing analysis

Genomic DNA was extracted from 65 BM aspirates and mutations associated with AML were detected by next-generation sequencing using the Foundation of Medicine panel (Foundation Medicine, Cambridge, MA). More information can be found in the Additional file 15.

### Statistical analysis

Correlation between protein expressions, clinical and laboratory features and outcome were determined using Chi‐Square and Fisher’s exact test for categorical variables and the Kruskal–Wallis test for continuous variables. Survival curves were generated using the Kaplan–Meier estimator [56].

## Supplementary Information

**Additional file 1: Figure S1.** Relative quantities of histone methylation marks in a total of 241 peripheral blood and bone marrow samples obtained from acute myeloid leukemia patients. **a** H3K4Me2 expression levels per French-American-British (FAB) subtype; **b** H3K4Me2 expression levels per World Health Organization (WHO) risk group; **c** H3K4Me2 expression level per cytogenetic subgroup; **d** H3K4Me3 expression levels per FAB subtype; **e** H3K4Me3 expression levels per WHO risk group; **f** H3K4Me3 expression level per cytogenetic subgroup; **g** H3K27Me3 expression levels per FAB subtype; **h** H3K27Me3 expression levels per WHO risk group; **i** H3K27Me3 expression level per cytogenetic subgroup.

**Additional file 2: Figure S2.** Relative quantity of H3K4Me2 in peripheral blood and bone marrow samples obtained from acute myeloid leukemia patients **a** who received prior chemotherapy (*n* = 23) and those who did not (*n* = 217); **b** who received prior radiotherapy (*n* = 21) and those who did not (*n* = 219); **c** who had a FLT3-D835 mutation (*n* = 19) compared to those who had FLT3-D835 wildtype (*n* = 204). Patients with the FLT3-D835 mutation had lower H3K4Me2 expression levels (Wilcoxon, *p* = 0.013); **d** H3K4Me2 negatively correlated with the percentage of monocytes in the bone marrow (*r* =  − 0.22, *p* < 0.01); **e** the percentage of monocytes in the peripheral blood (*r* =  − 0.2, *p* < 0.01) and **f** with the platelet count (*r* =  − 0.14, *p* = 0.033); **g** H3K4Me2 positively correlated with the presence of the surface marker CD13 (*r* = 0.16, *p* = 0.016); **h** H3K4Me2 negatively correlated with the presence of the surface marker CD33 (*r* =  − 0.15, *p* = 0.02); **i** H3K4Me2 positively correlated with the presence of the surface marker CD34 (*r* = 0.21, *p* < 0.01).

**Additional file 3: Figure S3.** Relative quantity of H3K4Me3 in peripheral blood and bone marrow samples obtained from acute myeloid leukemia patients was **a** significantly higher in patients who received prior chemotherapy (*n* = 23) compared to those who did not (*n* = 217, Wilcoxon, *p* = 0.019); **b** significantly higher in patients who received prior radiotherapy (*n* = 21) compared to those who did not (*n* = 219, Wilcoxon, *p* = 0.043). H3K4Me3 and **c** significantly lower in patients with the FLT3-D835 mutation (*n* = 19) compared to those who had FLT3-D835 wildtype (*n* = 204, Wilcoxon, *p* = 0.029). **d** H3K4Me3 negatively correlated with the percentage of monocytes in the bone marrow (*r* =  − 0.15, *p* = 0.017); **e** the percentage of monocytes in the peripheral blood (*r* =  − 0.19, *p* < 0.01); **f** and the platelet count (*r* =  − 0.15, *p* = 0.019); **g** H3K4Me3 positively correlated with the presence of the surface marker CD13 (*r* = 0.13, *p* = 0.043); **h** H3K4Me3 negatively correlated with the presence of the surface marker CD33 (*r* =  − 0.16, *p* = 0.014); **i** H3K4Me3 positively correlated with the presence of the surface marker CD34 (*r* = 0.14, *p* = 0.037).

**Additional file 4: Figure S4.** Relative quantity of H3K27Me3 in peripheral blood and bone marrow samples obtained from acute myeloid leukemia patients was **a** significantly lower in patients who experienced antecedent hematologic disorder (*n* = 81) compared to those without (*n* = 140, Wilcoxon, *p* = 0.035); **b** lower in patients with the FLT3-D835 mutation (*n* = 19) compared to those without (*n* = 204, Wilcoxon, *p* = 0.068) and **c** significantly higher in patients who received a transplantation (*n* = 233) compared to the ones that were not transplanted (*n* = 208, Wilcoxon, *p* < 0.01). **d** H3K27Me3 negatively correlated with the percentage of monocytes in the bone marrow (*r* =  − 0.20, *p* < 0.0); **e** the percentage of monocytes in the peripheral blood (*r* =  − 0.23, *p* < 0.01); **f** and the platelet count (*r* =  − 0.16, *p* = 0.012); **g** H3K27Me3 positively correlated with the presence of the surface marker CD7 (*r* = 0.15, *p* = 0.017); **h** CD19 (*r* = 0.16, *p* = 0.013) **i** H3K27Me3 negatively correlated with the presence of the surface marker CD33 (*r* =  − 0.13, *p* = 0.042); **j** age (*r* =  − 0.14, *p* = 0.031); **k** creatinine (*r* =  − 0.15, *p* = 0.022); **l** H3K27Me3 positively correlated with event-free survival duration (*r* = 0.13, *p* = 0.062).

**Additional file 5: Table S1.** H3K27me3, H3K4me2 and H3K4me3 levels (median, IQR) according to patient and disease characteristics in AML.

**Additional file 6: Table S2.** H3K27me3, H4K4me2, H3K4me3 levels (median, IQR) according to patient and disease characteristics in ALL.

**Additional file 7: Table S3.** Pairwise associations between driver mutations presented by Pearson's Correlation Coefficient (cor) and p-value (p).

**Additional file 8: Table S4.** Pairwise associations between affected pathways presented by Pearson's Correlation Coefficient (cor) and p-value (p).

**Additional file 9: Figure S5.** H3K27Me3 is significantly lower in **a** ASXL1 **b** BCOR **c** SRSF2 **d** U2AF1 mutated patients with acute myeloid leukemia (AML) compared to wildtype. *p < 0.05, ** *p* < 0.01. **e** Heatmap showing relative H3K27Me3 level per sample of 65 sequenced bone marrow aspirates from AML patients along with the presence (orange) or absence (gray) of ASXL1, BCOR, SRSF2 and/or U2AF1 mutations and subgroup H3K27Me3; H3K27Me3^low^ in blue and H3K27Me3^high^ in red. Patients with denoted mutations had lower levels H3K27Me3.

**Additional file 10: Figure S6.** **a** Waterfall plot showing significant correlated protein expressions between H3K27Me3 and the other 229 antibodies on the reverse phase protein array (RPPA) identified positively and negatively associated proteins. Negatively correlated proteins (Pearson correlation *R* > 0.25, *p* < 0.0001) that are supposed to be upregulated if H3K27Me3 was lost are red-lined. **b** String analysis reveals H3K27Me3 negatively associated proteins are interconnected in shared biological processes; in red: Negative regulation of programmed cell death (GO:0043069, FDR = 1.27e−14), in green: Regulation of cell population proliferation (GO:0042127, FDR = 1.31e − 14) and in blue: Negative regulation of apoptotic process (GO:0043066, FDR = 8.42e−14).

**Additional file 11: Table S5.** Enriched biological processes by Gene Ontology analysis.

**Additional file 12: Figure S7.** Higher expression levels of H3K4Me2 in **a** bone marrow (BM, *n* = 79) samples compared to peripheral blood (PB, *n* = 48) obtained from acute lymphoblastic leukemia patients (ALL, Wilcoxon, *p* = 0.022). **b** Higher H3K4Me2 in PB samples from ALL patients that underwent transplantation compared to their expression in PB samples from patients without (Wilcoxon, *p* = 0.017). **c** Lower H3K4Me2 was seen in 4 BM samples from patients that had a prior malignancy compared to H3K4Me2 level in BM from patients who had no prior malignancy (*n* = 75, Wilcoxon, *p* = 0.045). H3K4Me2 positively correlated with **d** the percentage of bone marrow blasts in BM samples (blue, *r* = 0.61, *p* < 0.01), but not in the PB samples (red). **e** The percentage peripheral blasts in BM samples (blue, *r* = 0.6, *p* < 0.01), but not in the PB samples (red). **f** H3K4Me2 positively correlated with the presence of the surface marker CD7 (*r* = 0.25, *p* = 0.031) in PB (red), but not in BM (blue); **g** H3K4Me2 negatively correlated with the presence of the surface marker HLA in the PB samples (*r* =  − 0.28, *p* = 0.013), but not in the BM.

**Additional file 13: Figure S8.** Higher expression levels of H3K4Me3 in **a** bone marrow (BM, *n* = 79) samples compared to peripheral blood (PB, *n* = 48) obtained from acute lymphoblastic leukemia patients (ALL, Wilcoxon, *p* = 0.012). H3K4Me3 positively correlated with **b** white blood count (WBC) in protein data from PB samples (red, *r* = 0.25, *p* = 0.025) and BM (blue, *r* = 0.34, *p* = 0.016); **c** absolute blast count in protein data from PB samples (red, *r* = 0.25, *p* = 0.025) and BM (blue, *r* = 0.35, *p* = 0.015); **d** percentage BM blasts in PB samples (red, *r* = 0.24, *p* = 0.034) and BM (blue, *r* = 0.65, *p* < 0.01); **e** percentage peripheral blasts in BM samples (blue, *r* = 0.63, *p* < 0.01), but not in PB samples (red, *r* = 0.19, *p* = 0.091). **f** Albumin levels in BM samples (blue, *r* = 0.31, *p* = 0.036).

**Additional file 14: Figure S9.** Higher expression levels of H327Me3 in **a** bone marrow (BM, *n* = 79) samples compared to peripheral blood (PB, *n* = 48) obtained from acute lymphoblastic leukemia patients (ALL, Wilcoxon, *p* < 0.01). **b** Higher H327Me3 in PB samples from ALL patients that underwent transplantation compared to their expression in PB samples from patients without (Wilcoxon, *p* < 0.01). **c** Lower H327Me3 was seen in 14 BM samples from ALL patients that experienced an infection compared to levels in BM samples from patients with no infection (Wilcoxon, *p* < 0.01) and therefore as well in patients who **e** experienced an event (*n* = 37 including 14 patients that had complicated infection, *p* = 0.019). H327Me3 positively correlated with **e** white blood count (WBC) in BM samples (blue, *r* = 0.29, *p* = 0.043), but not in the PB samples (red). **f** The absolute blast count in BM samples (blue, *r* = 0.3, *p* = 0.039), but not in the PB samples (red). **g** The percentage BM blasts in BM samples (blue, *r* = 0.54, *p* < 0.01) and PB (red, *r* = 0.22, *p* = 0.061); **h** the percentage peripheral blasts in BM samples (*r* = 0.56, *p* < 0.01), but not in PB samples. **i** H327Me3 negatively correlated with the presence of the surface marker CD13 in the PB samples (*r* =  − 0.28, *p* = 0.014), but not in the BM samples.

**Additional file 15.** Supplementary information sequencing analysis.

## Data Availability

The datasets used and analysed during the current study are available from the corresponding author on reasonable request.
