# Supplementary figures and images for: Loss of H3K27 methylation identifies poor outcomes in adult-onset acute leukemia
Source: Clin Epigenetics. 2021 Jan 28;13:21. doi: 10.1186/s13148-021-01011-x (PMC7841917; doi:10.1186/s13148-021-01011-x)

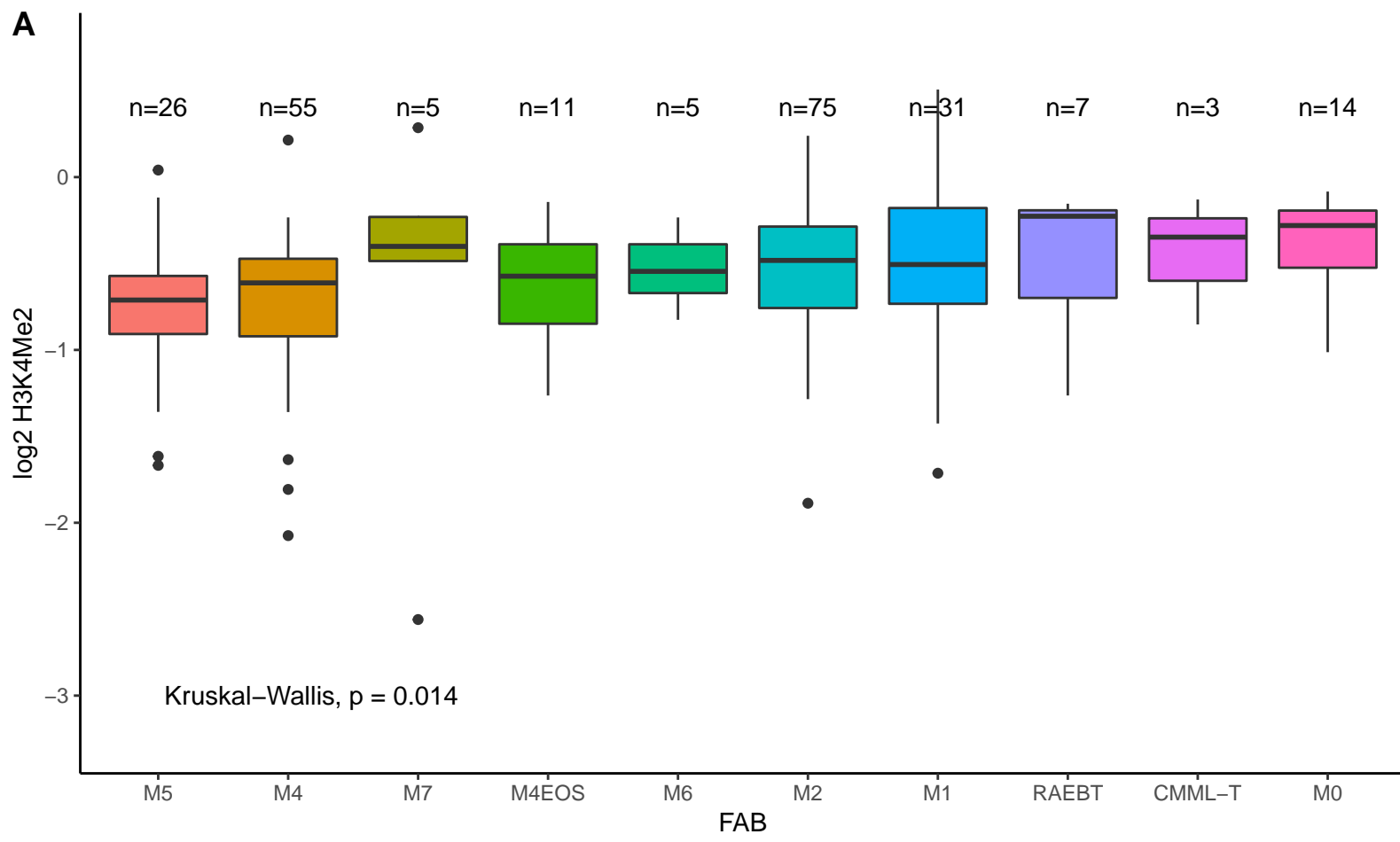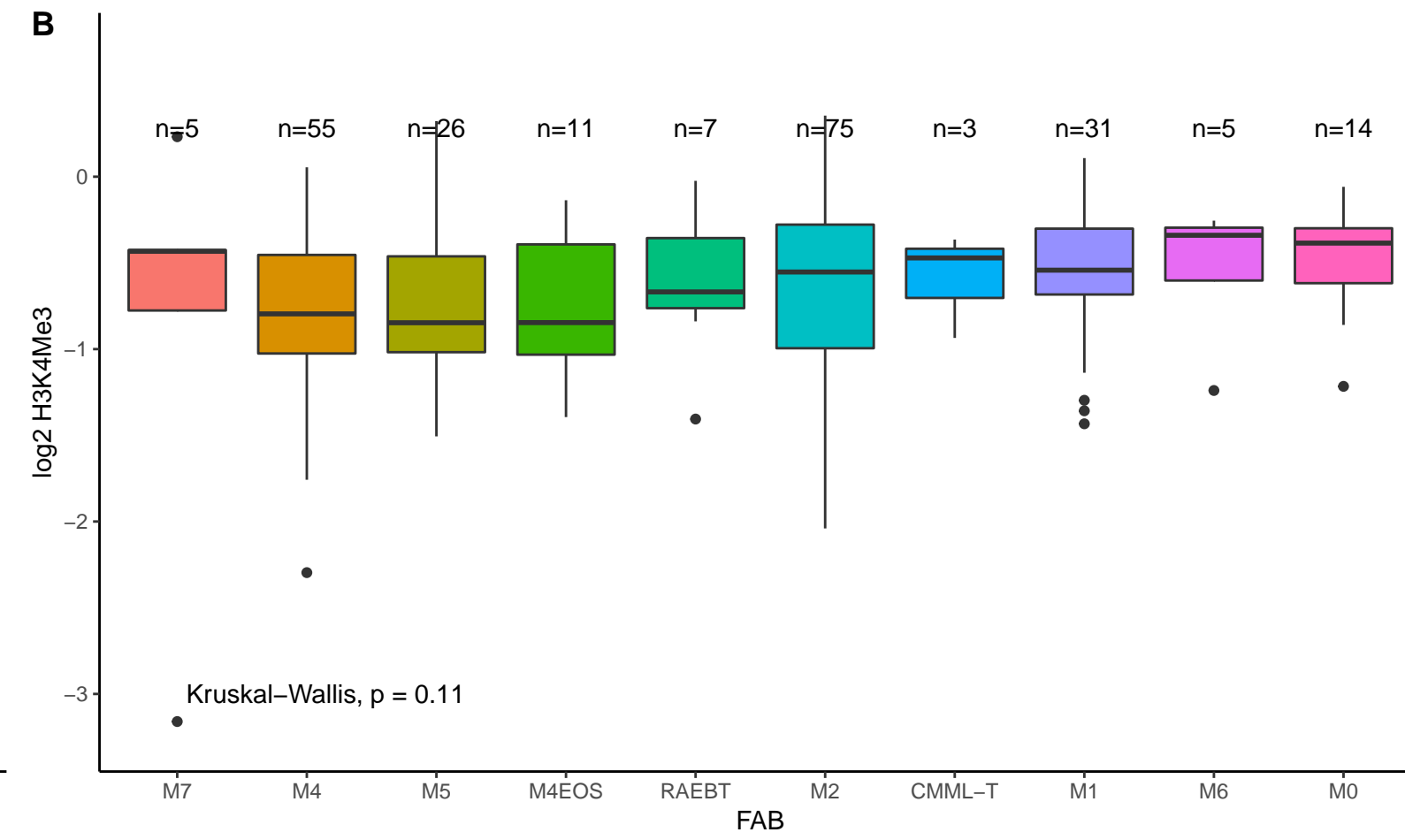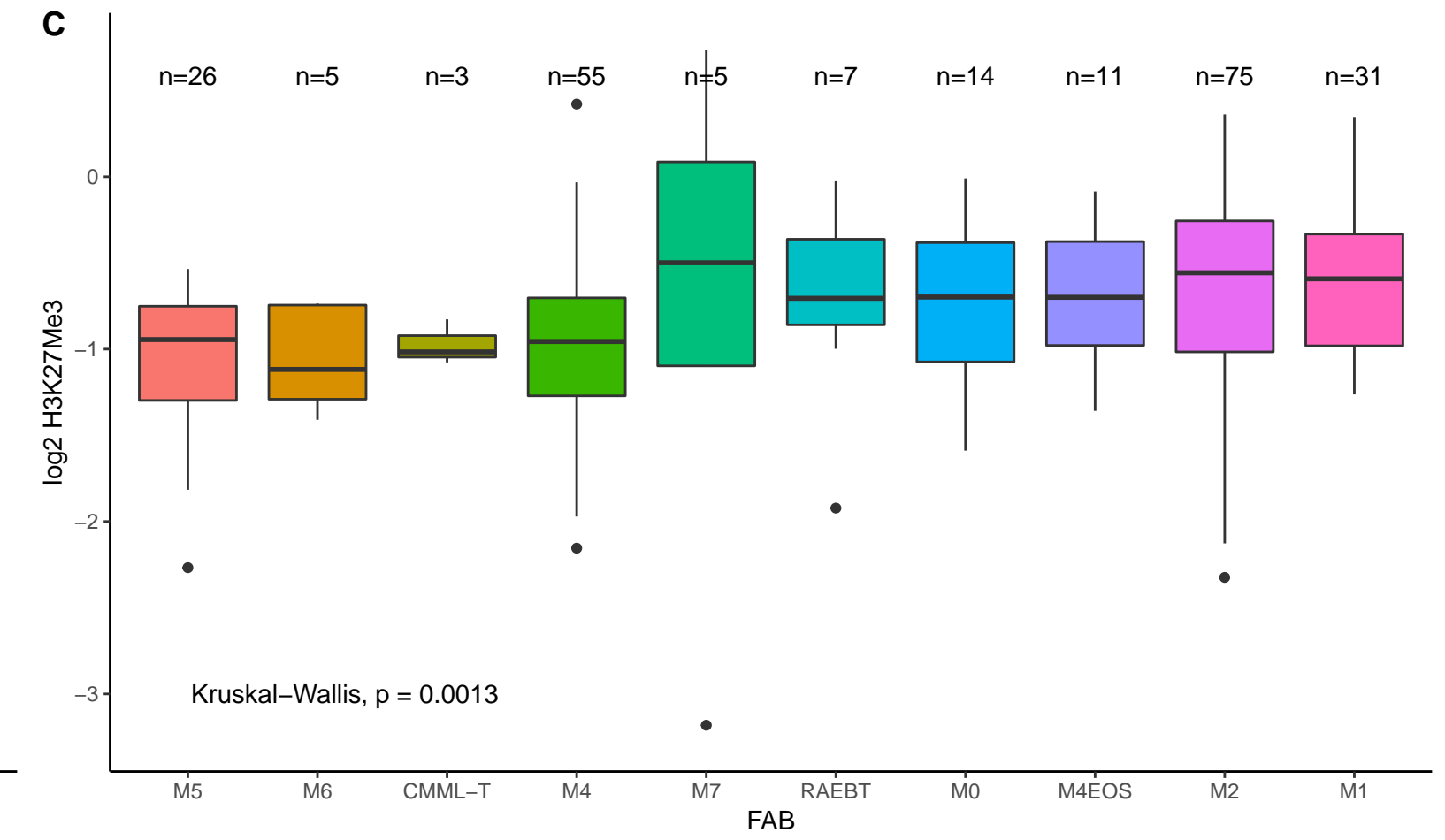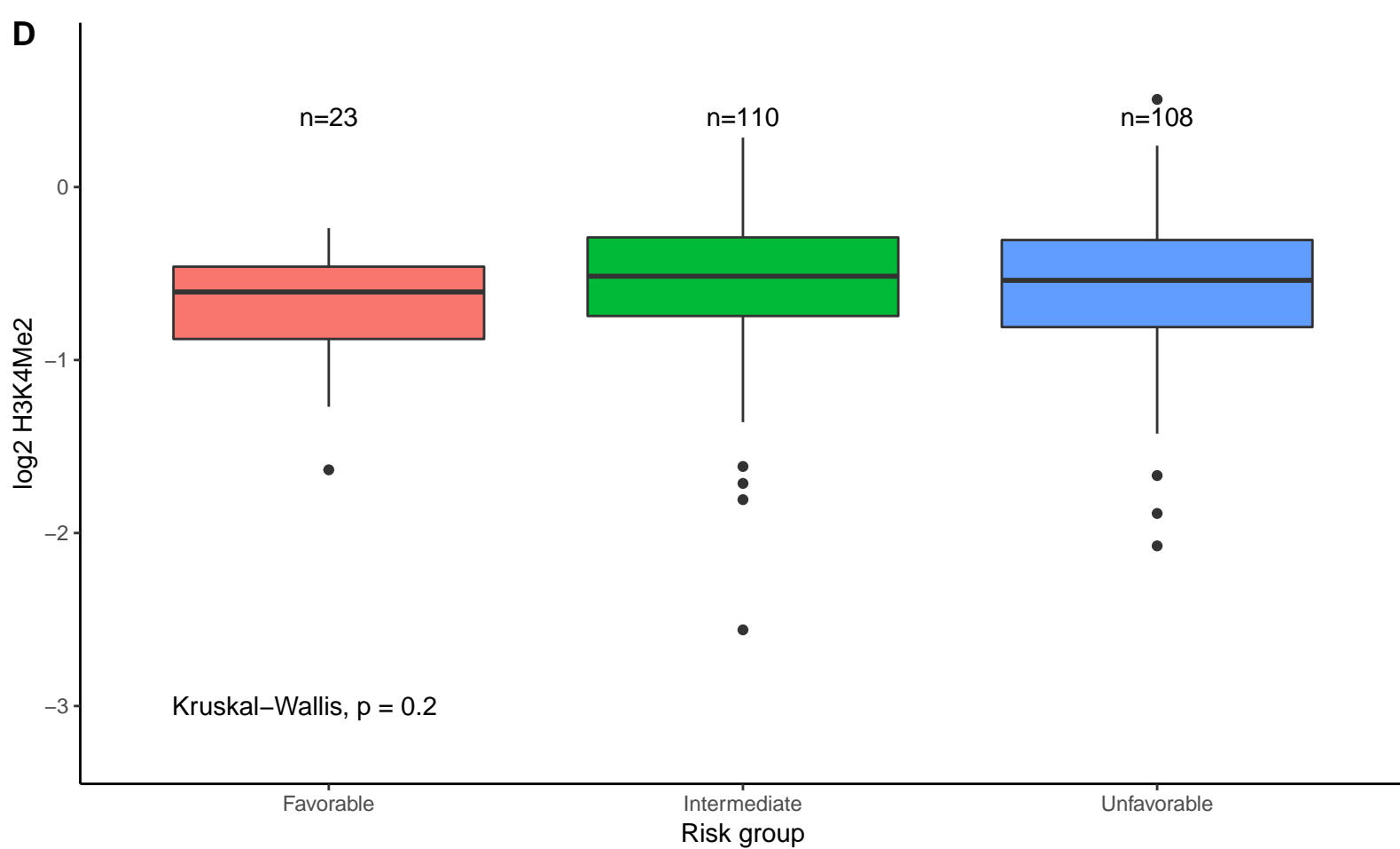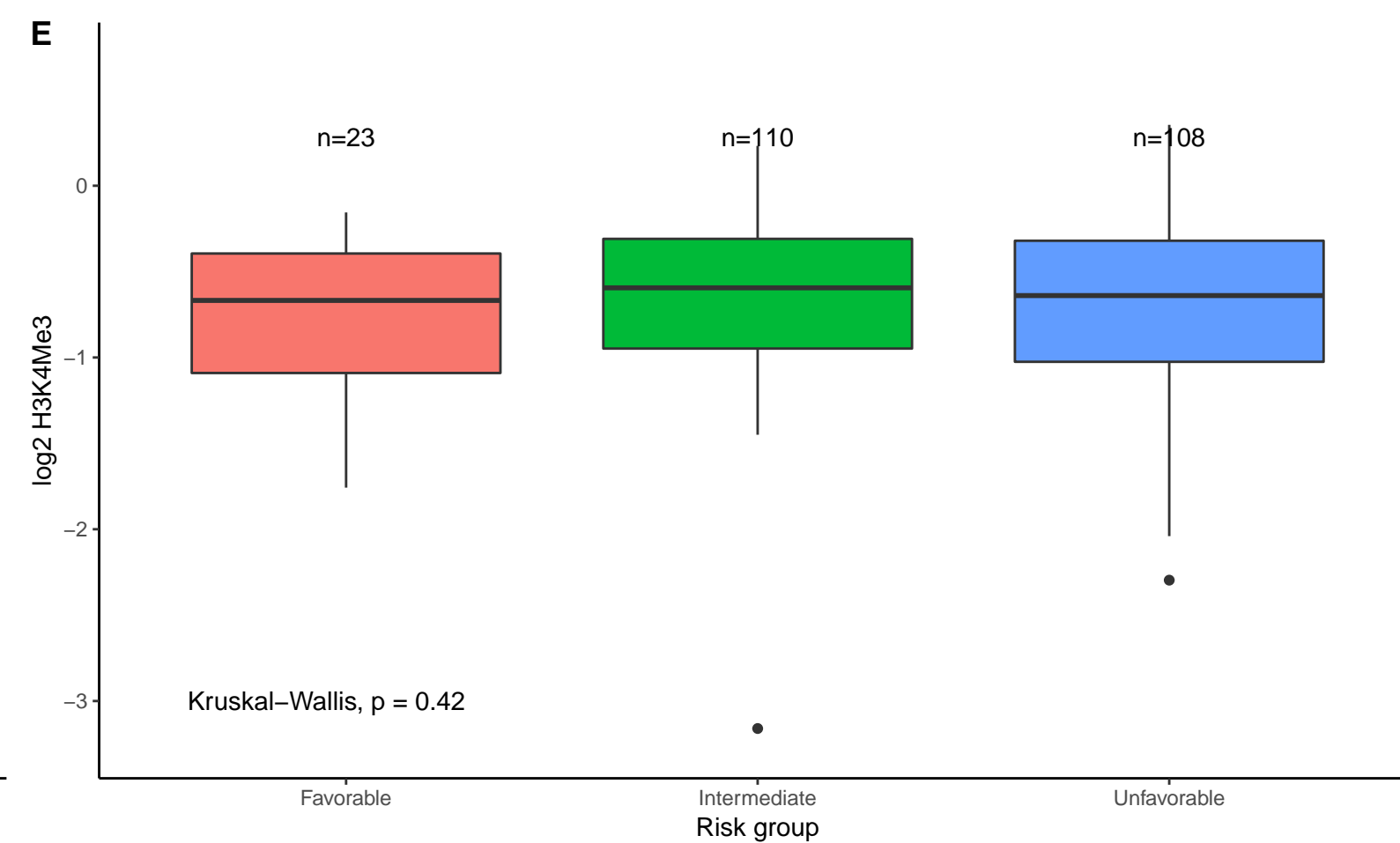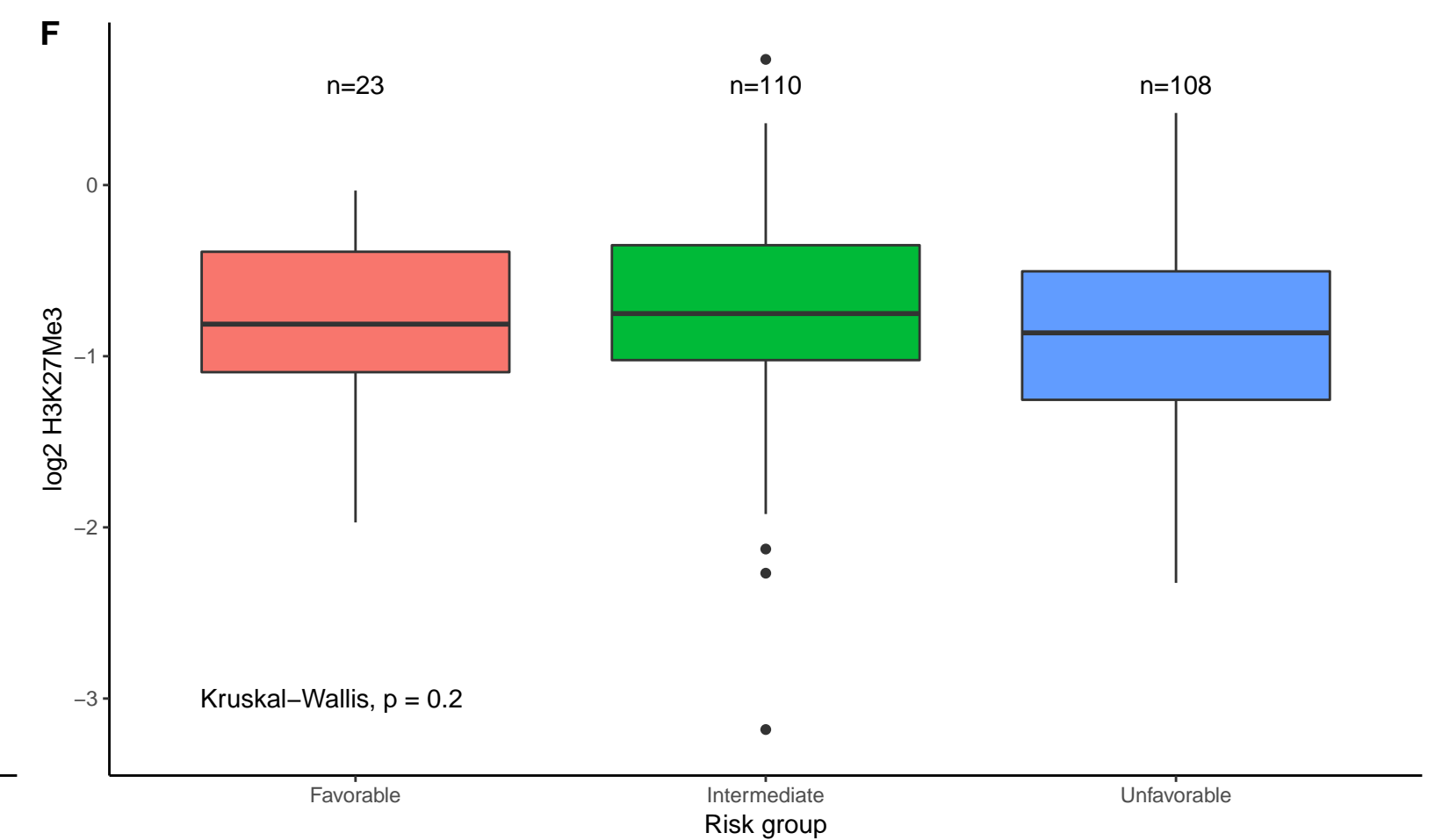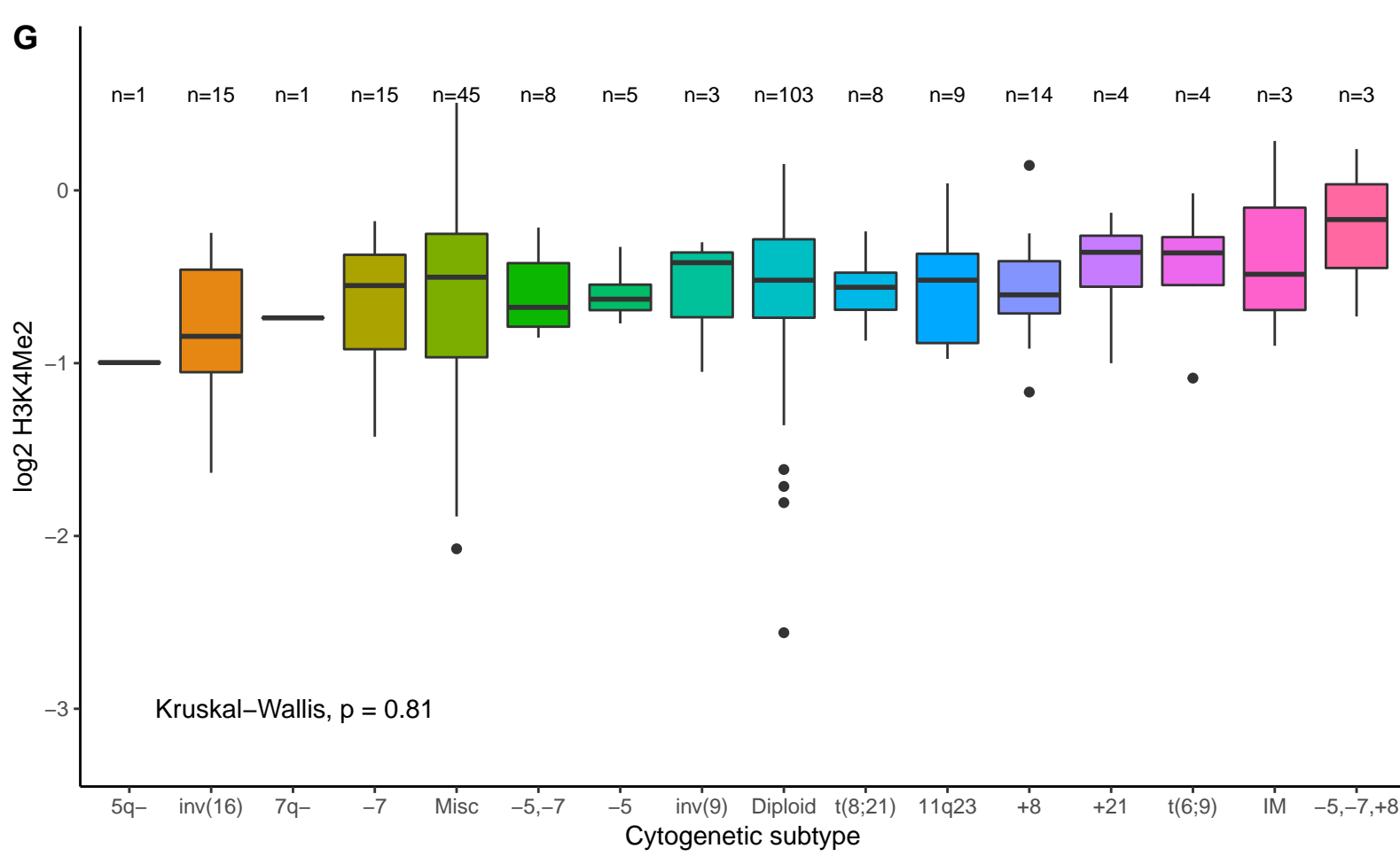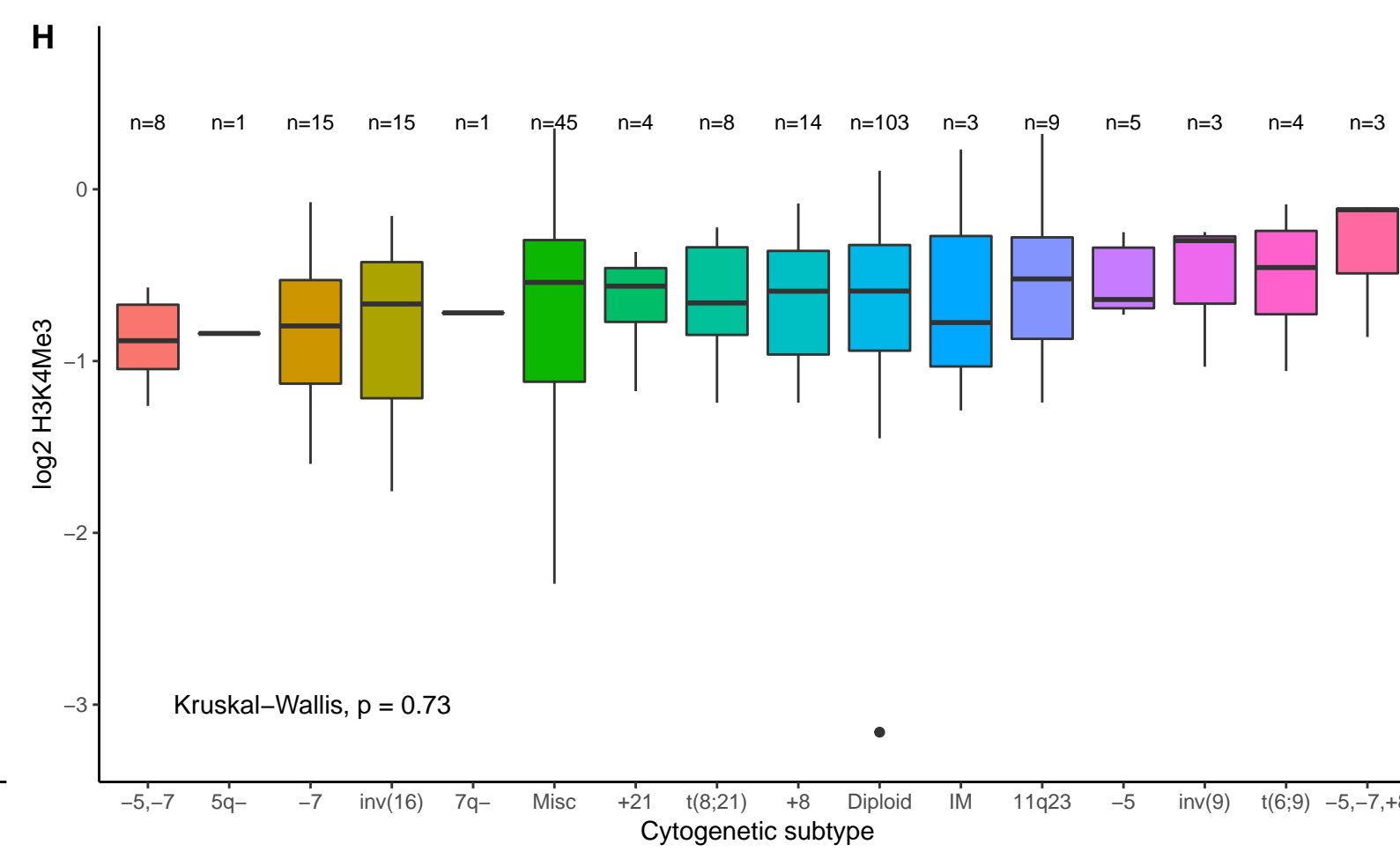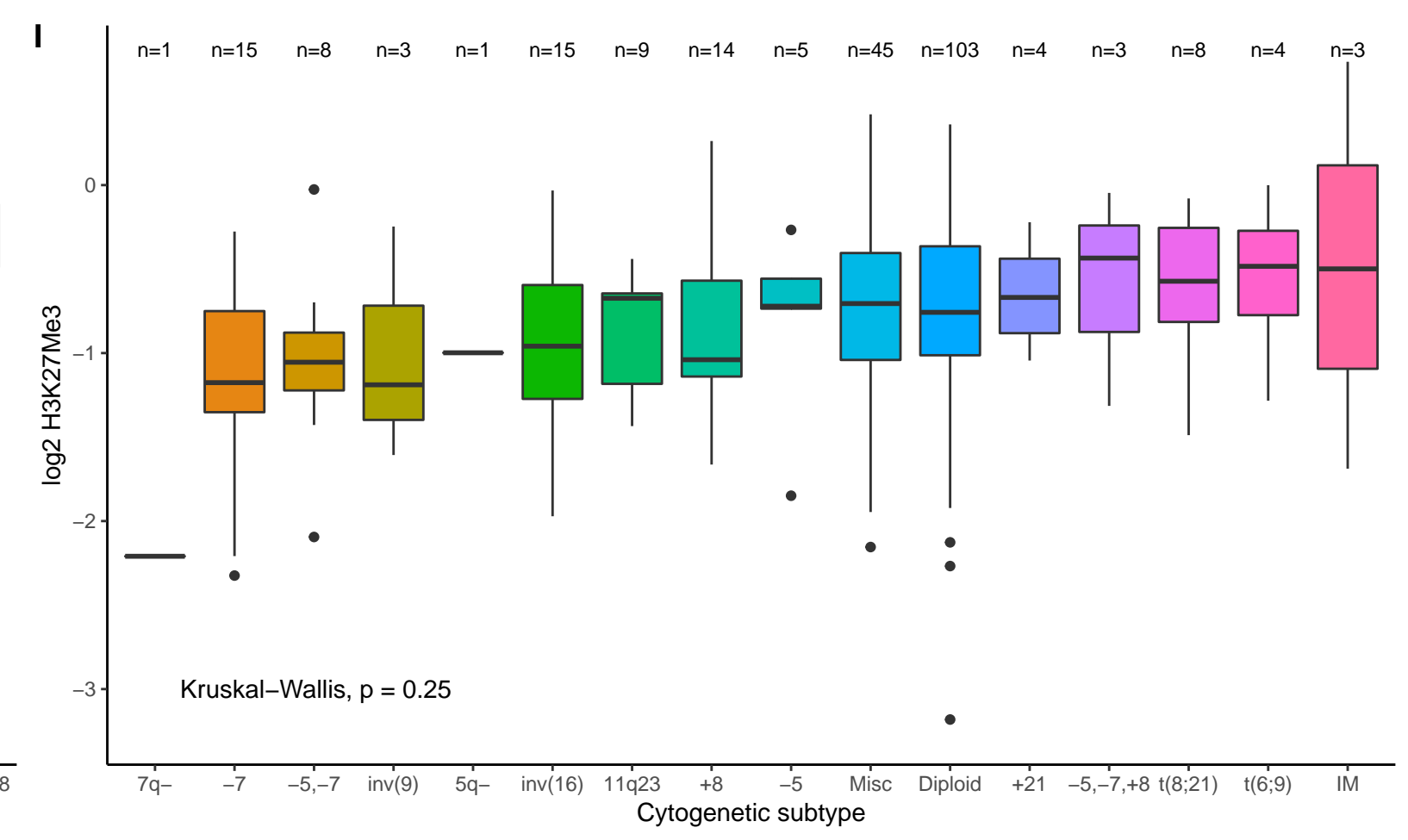

Supplement: Supplementary file 1 — Additional file 1: Figure S1. Relative quantities of histone methylation marks in a total of 241 peripheral blood and bone marrow samples obtained from acute myeloid leukemia patients. a H3K4Me2 expression levels per French-American-British (FAB) subtype; b H3K4Me2 expression levels per World Health Organization (WHO) risk group; c H3K4Me2 expression level per cytogenetic subgroup; d H3K4Me3 expression levels per FAB subtype; e H3K4Me3 expression levels per WHO risk group; f H3K4Me3 expression level per cytogenetic subgroup; g H3K27Me3 expression levels per FAB subtype; h H3K27Me3 expression levels per WHO risk group; i H3K27Me3 expression level per cytogenetic subgroup. [file 13148_2021_1011_MOESM1_ESM.pdf]

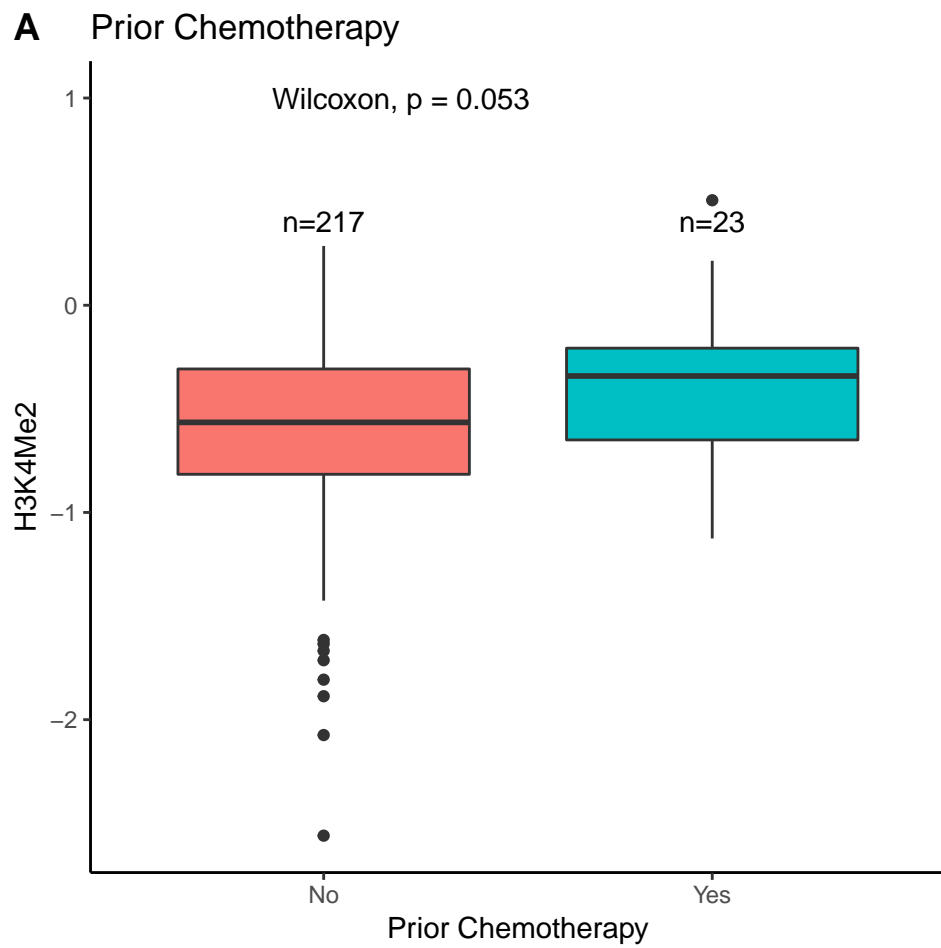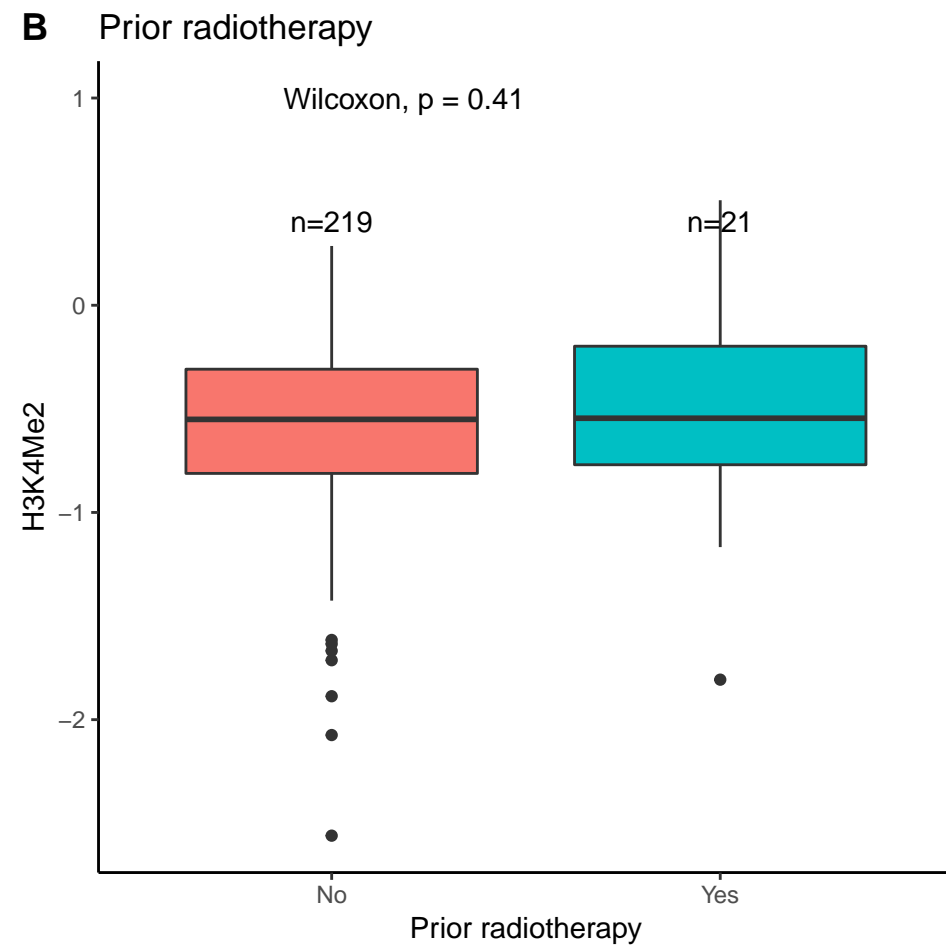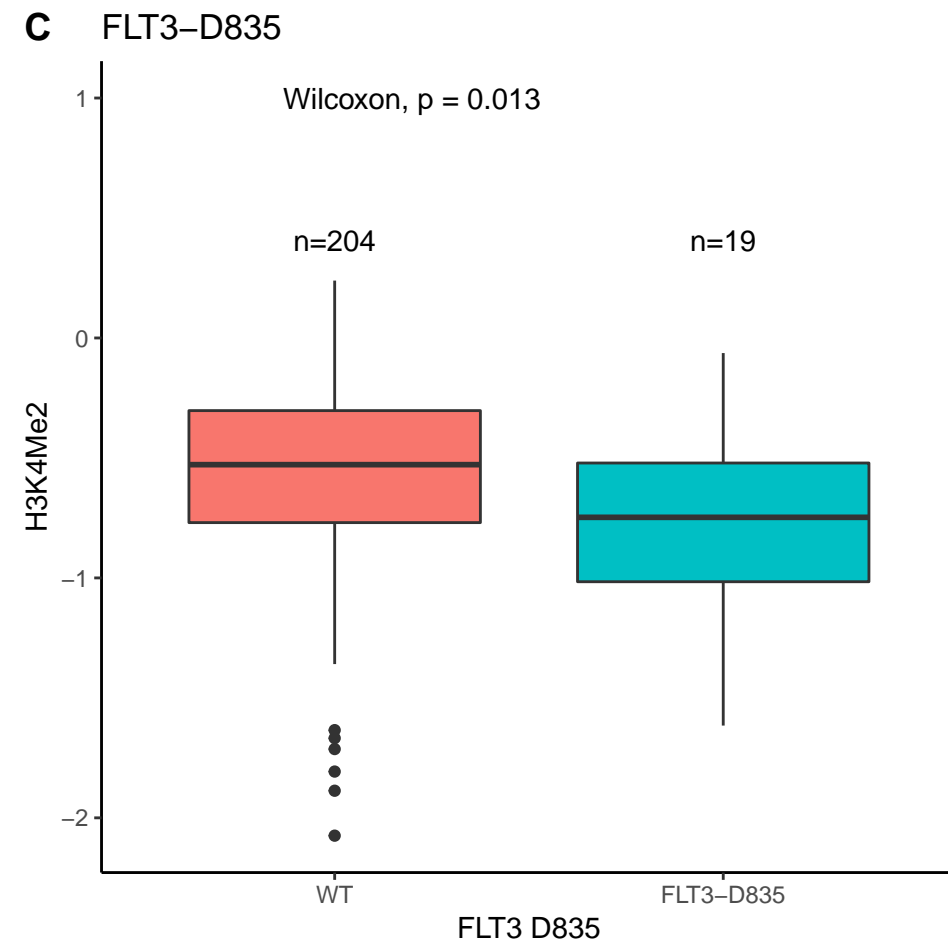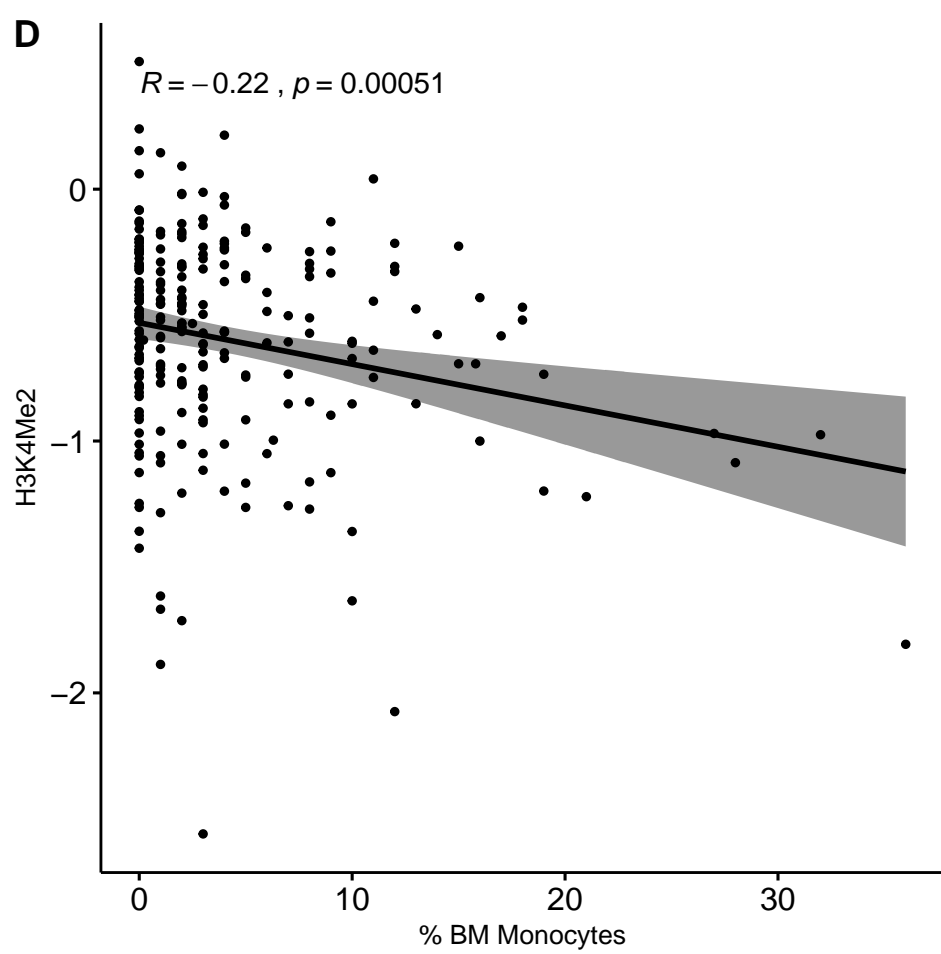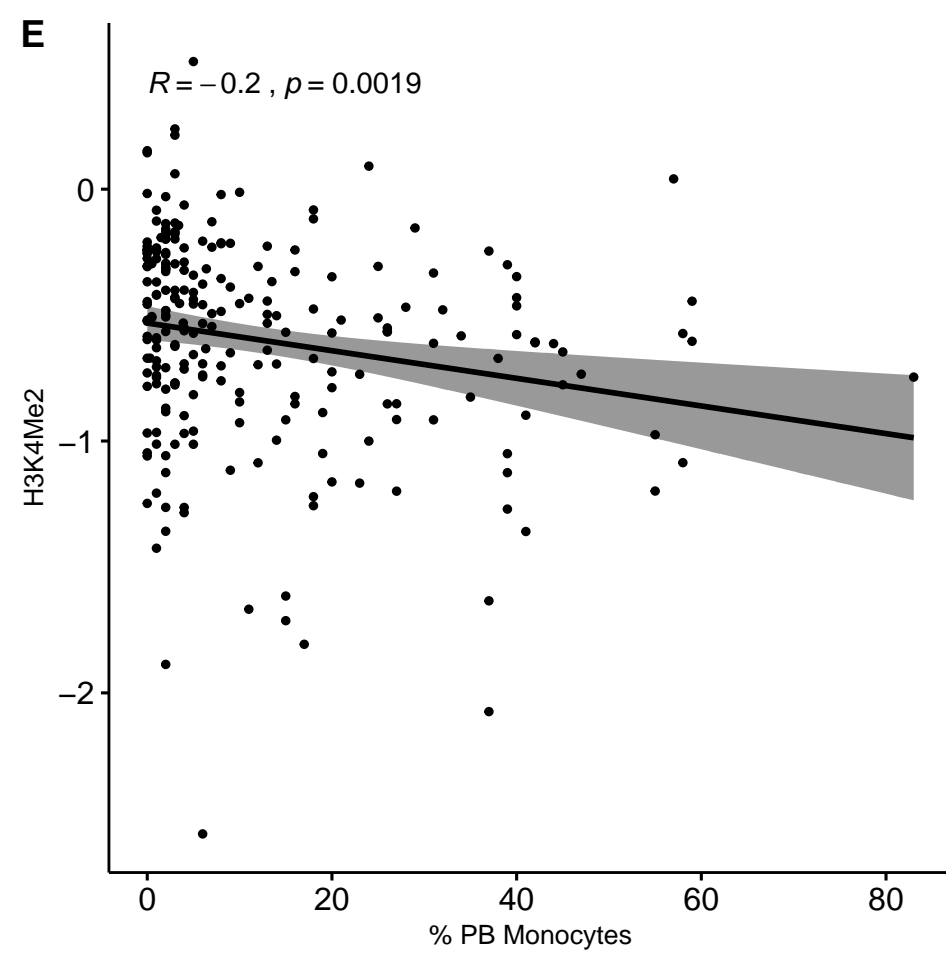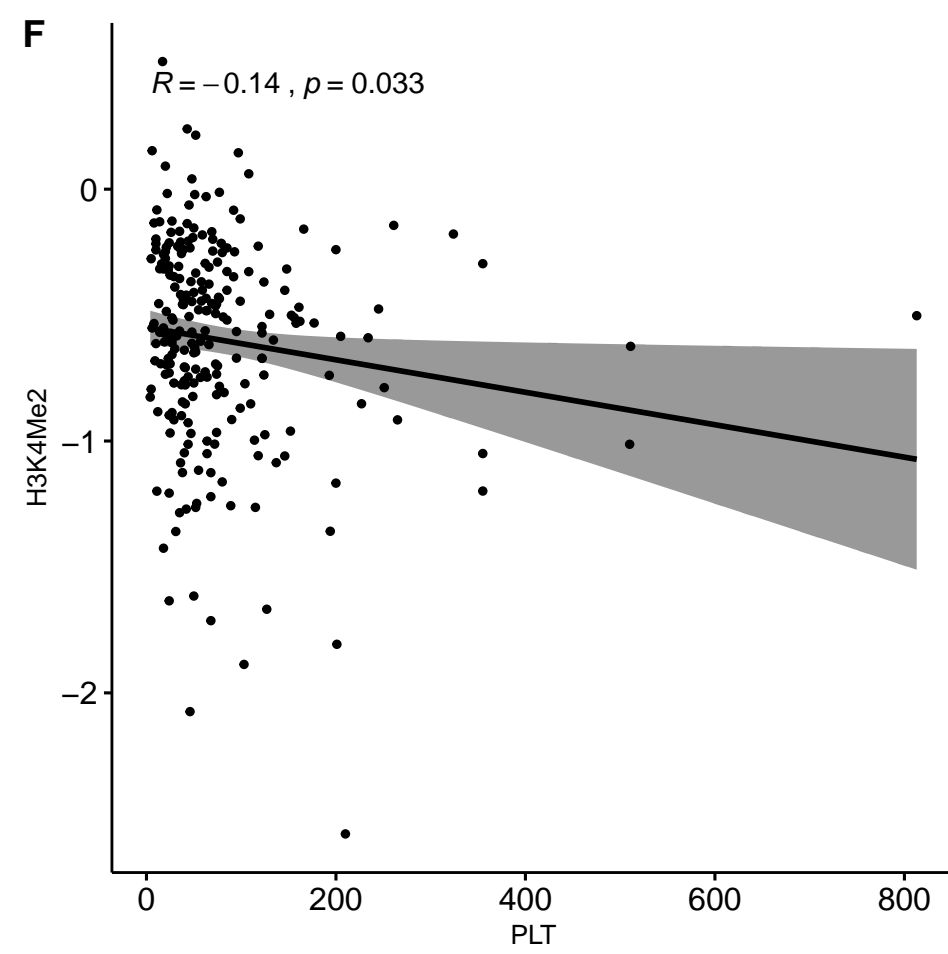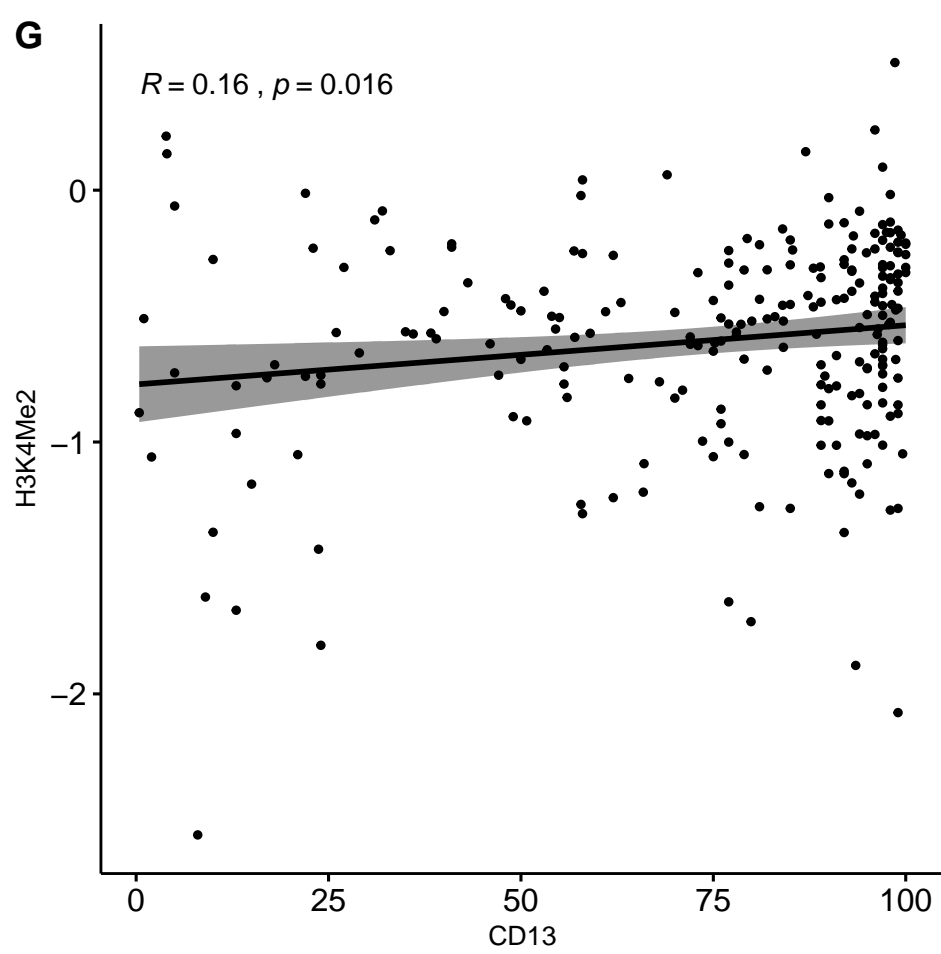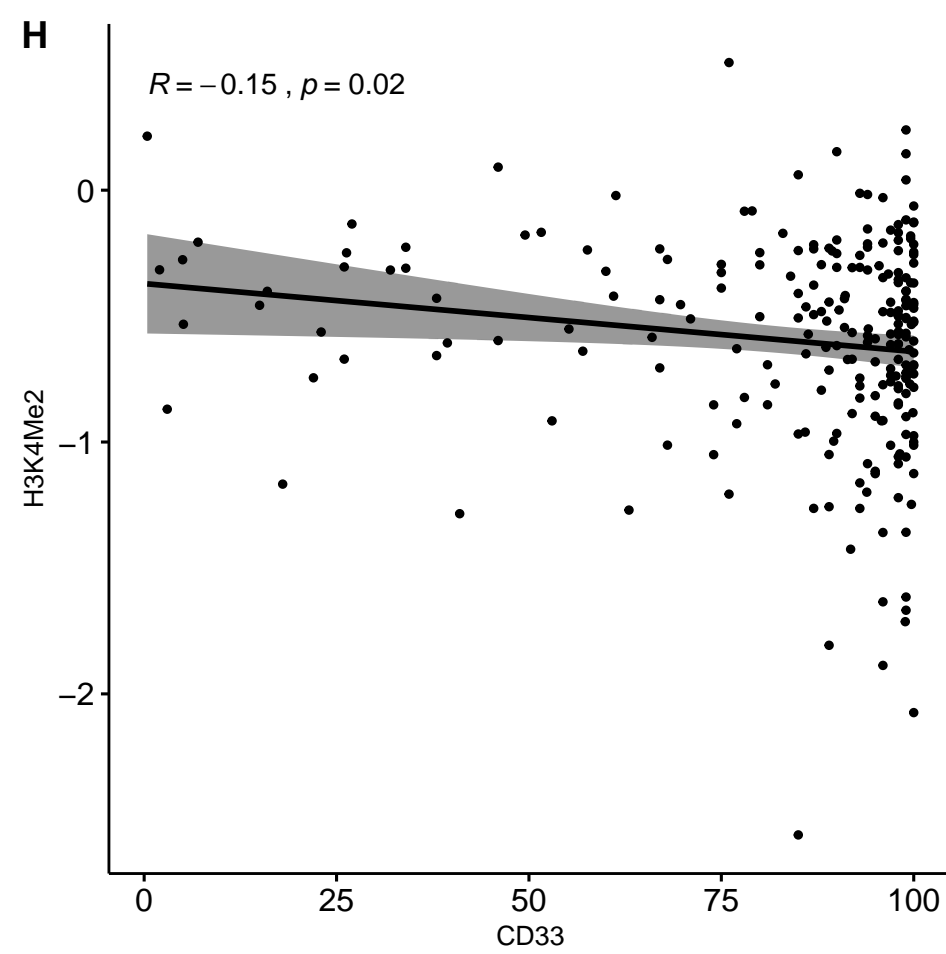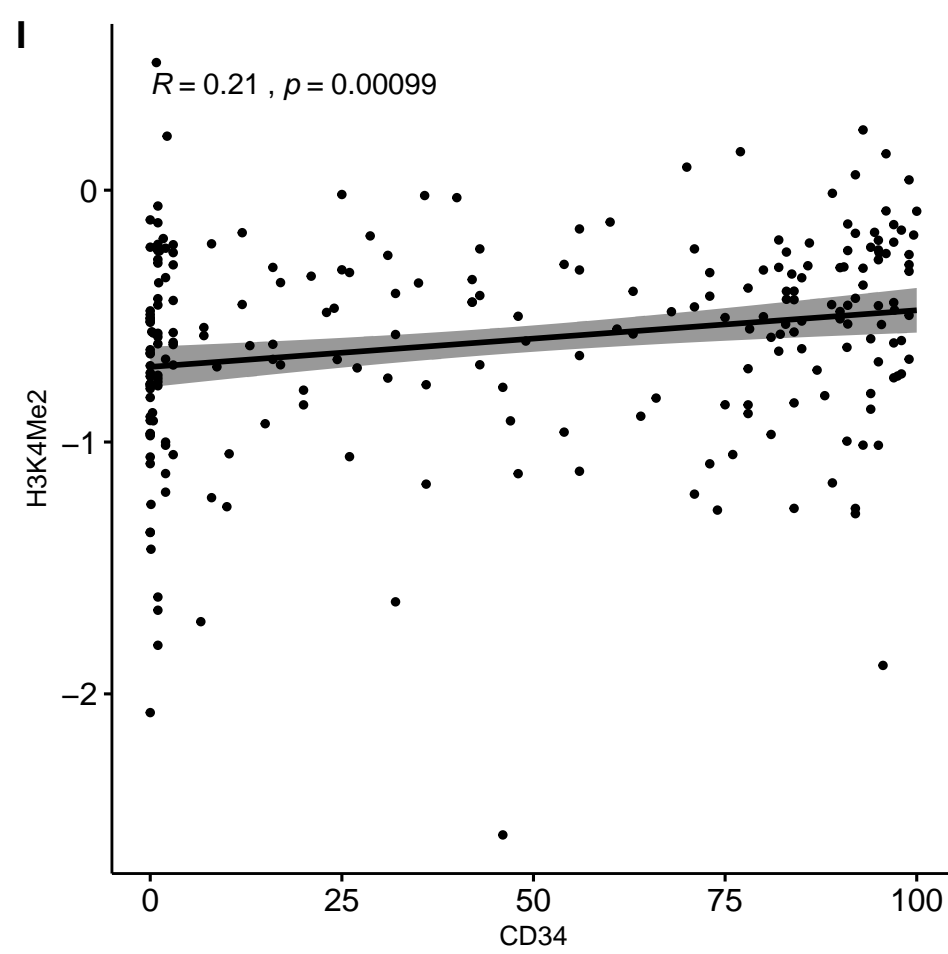

Supplement: Supplementary file 2 — Additional file 2: Figure S2. Relative quantity of H3K4Me2 in peripheral blood and bone marrow samples obtained from acute myeloid leukemia patients a who received prior chemotherapy (n = 23) and those who did not (n = 217); b who received prior radiotherapy (n = 21) and those who did not (n = 219); c who had a FLT3-D835 mutation (n = 19) compared to those who had FLT3-D835 wildtype (n = 204). Patients with the FLT3-D835 mutation had lower H3K4Me2 expression levels (Wilcoxon, p = 0.013); d H3K4Me2 negatively correlated with the percentage of monocytes in the bone marrow (r = − 0.22, p < 0.01); e the percentage of monocytes in the peripheral blood (r = − 0.2, p < 0.01) and f with the platelet count (r = − 0.14, p = 0.033); g H3K4Me2 positively correlated with the presence of the surface marker CD13 (r = 0.16, p = 0.016); h H3K4Me2 negatively correlated with the presence of the surface marker CD33 (r = − 0.15, p = 0.02); i H3K4Me2 positively correlated with the presence of the surface marker CD34 (r = 0.21, p < 0.01). [file 13148_2021_1011_MOESM2_ESM.pdf]

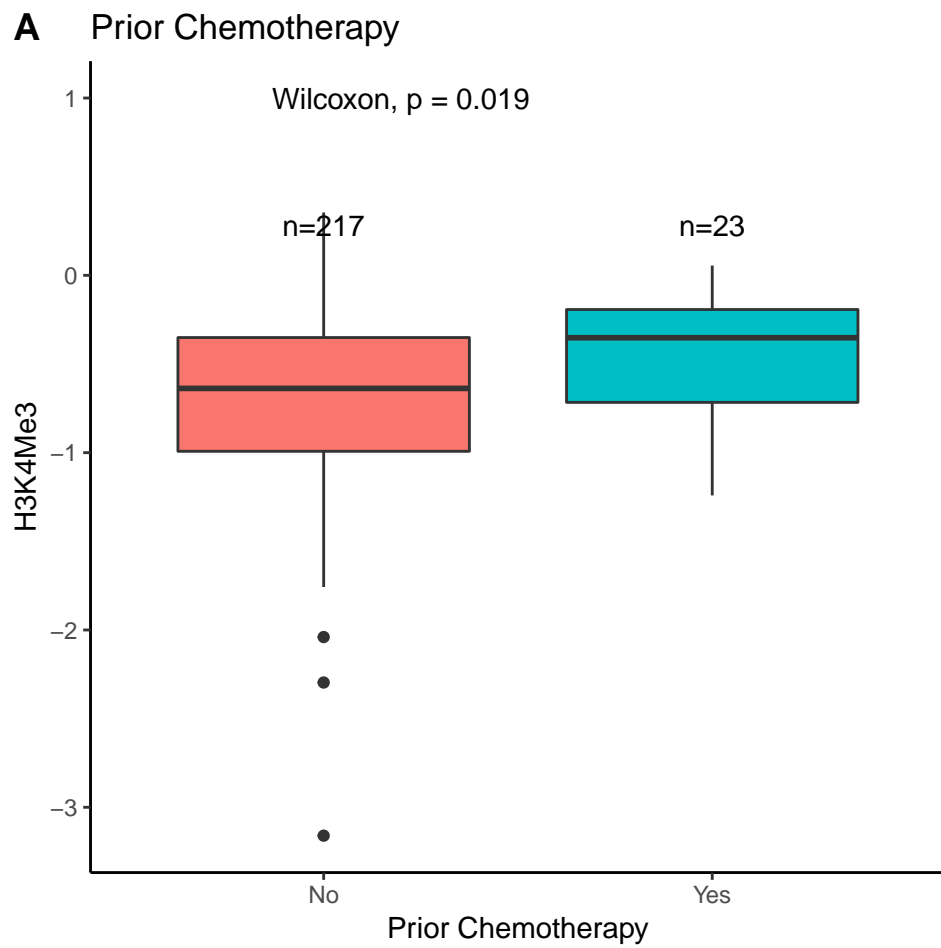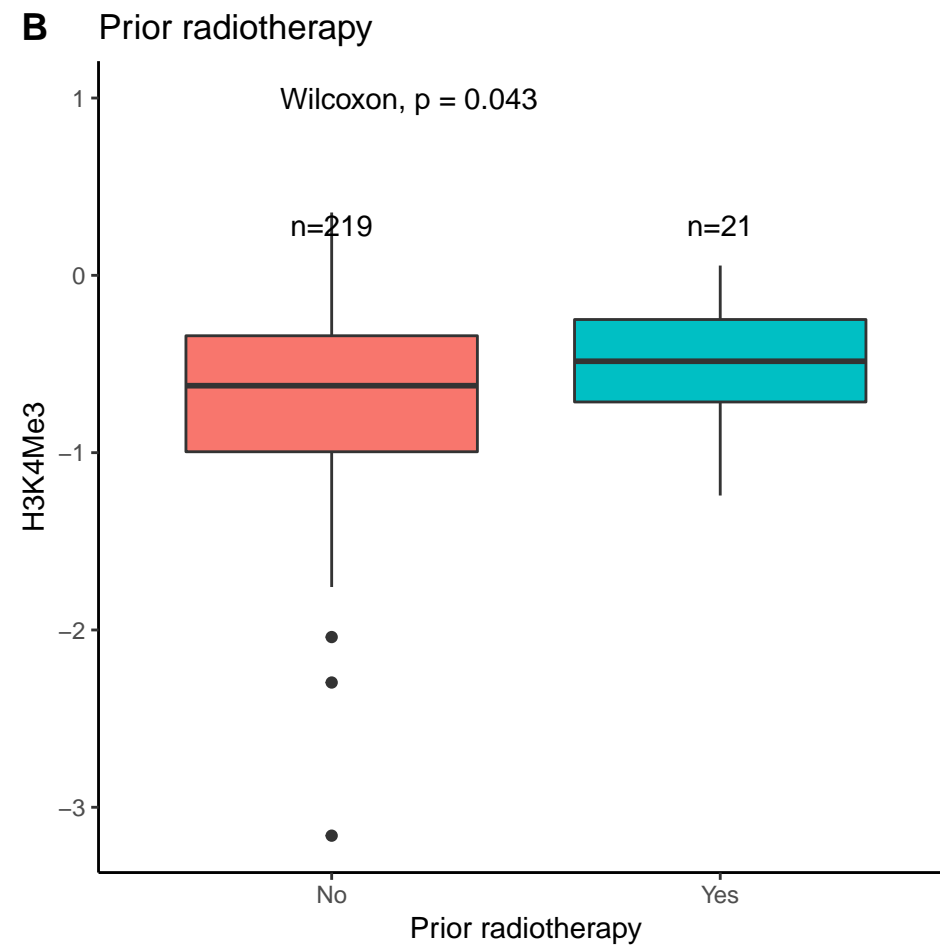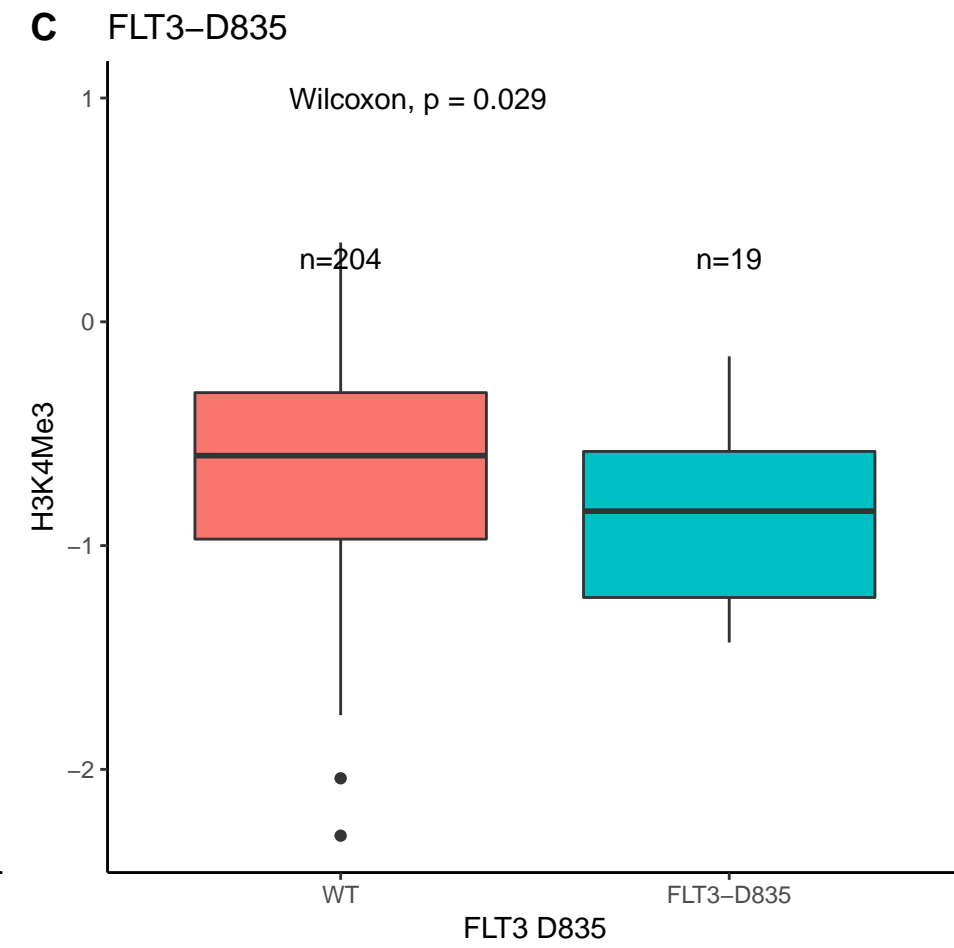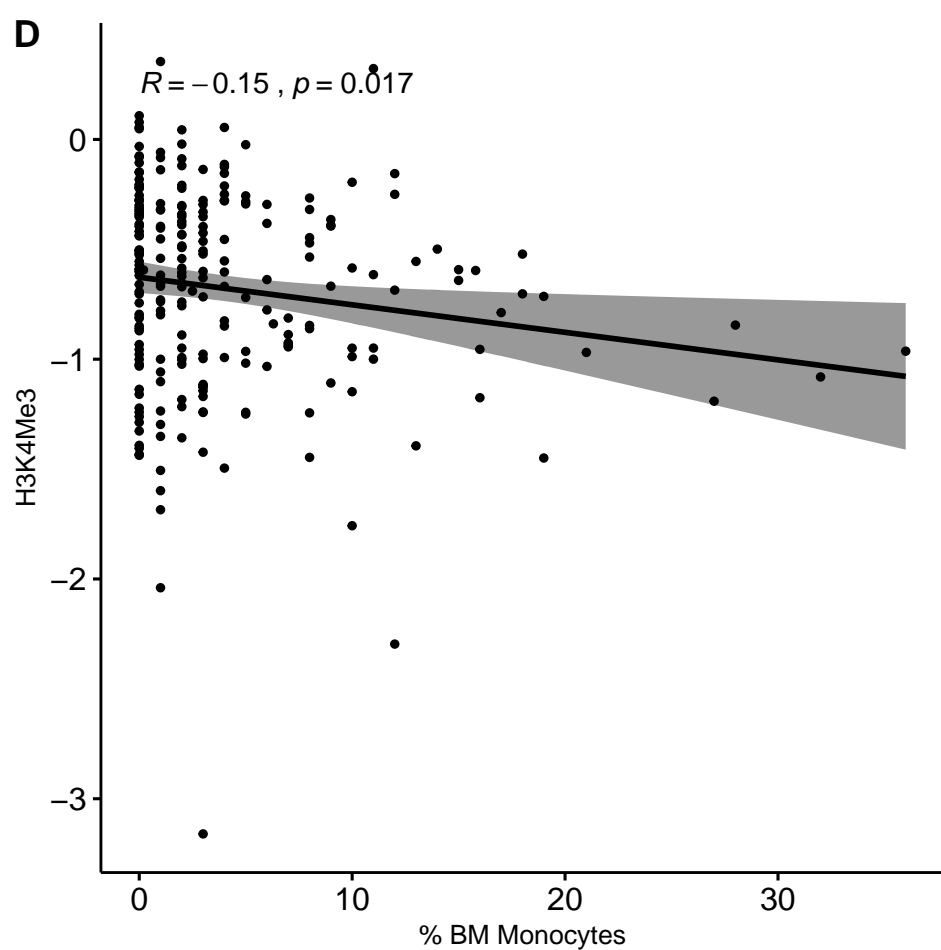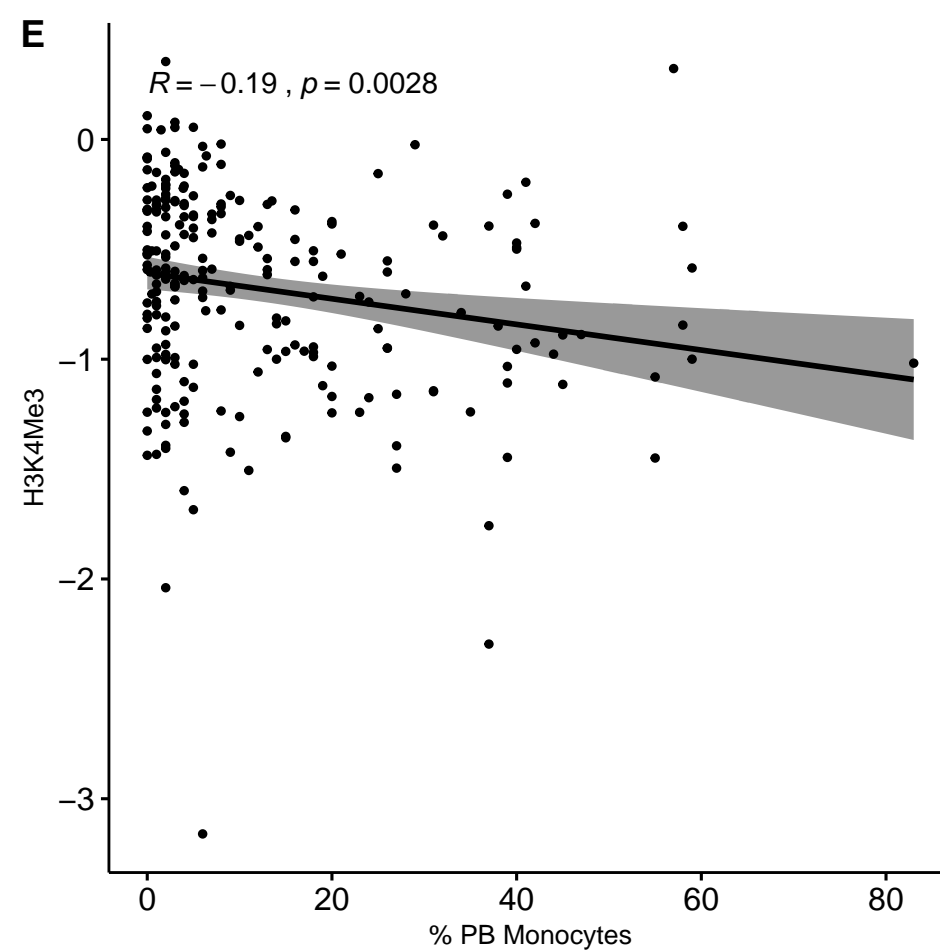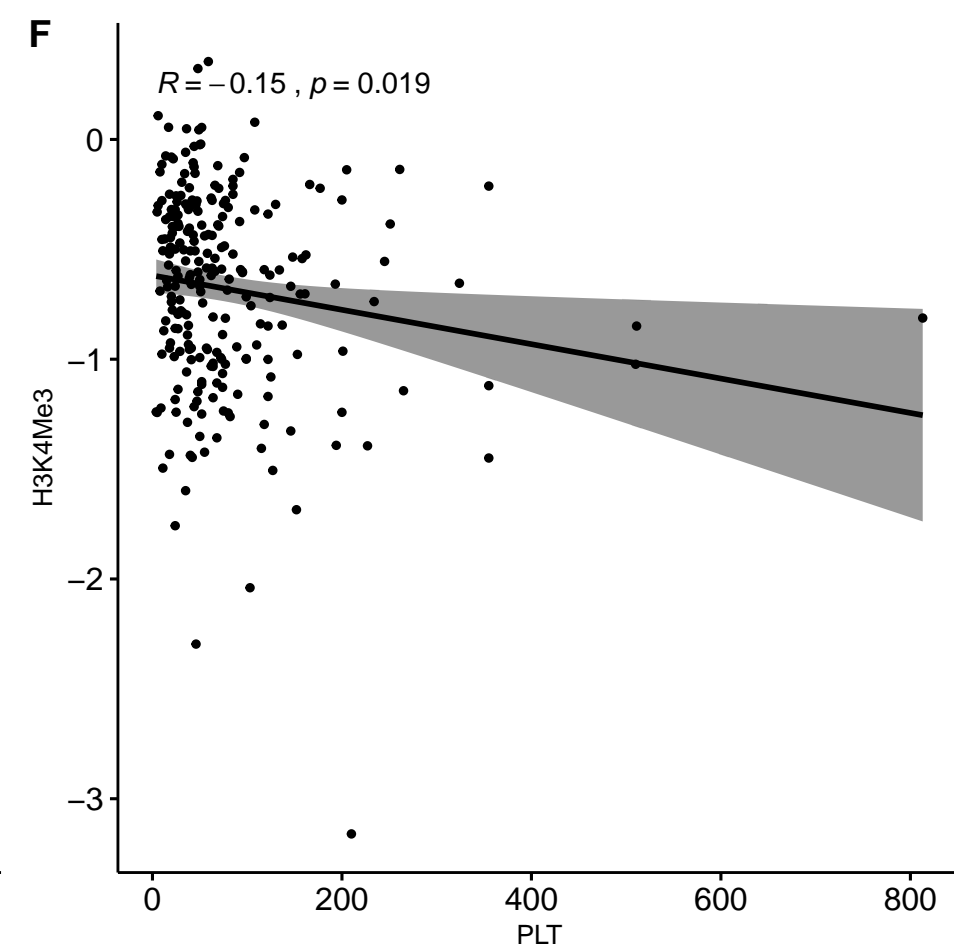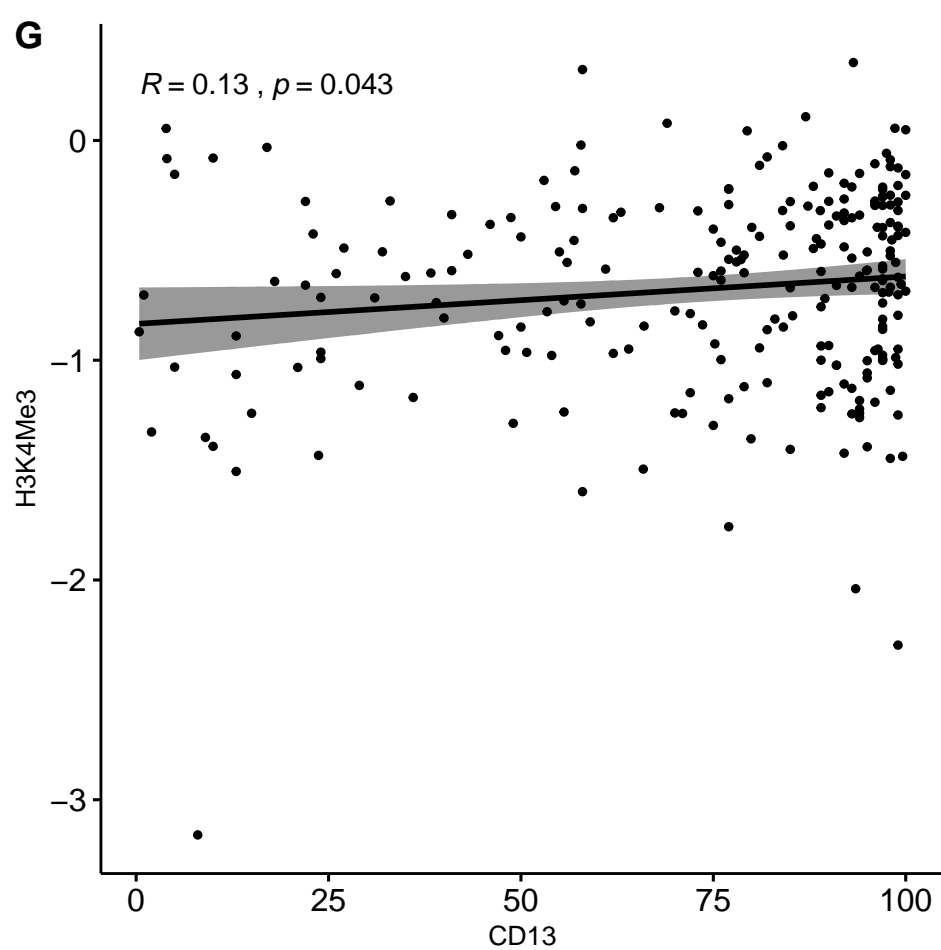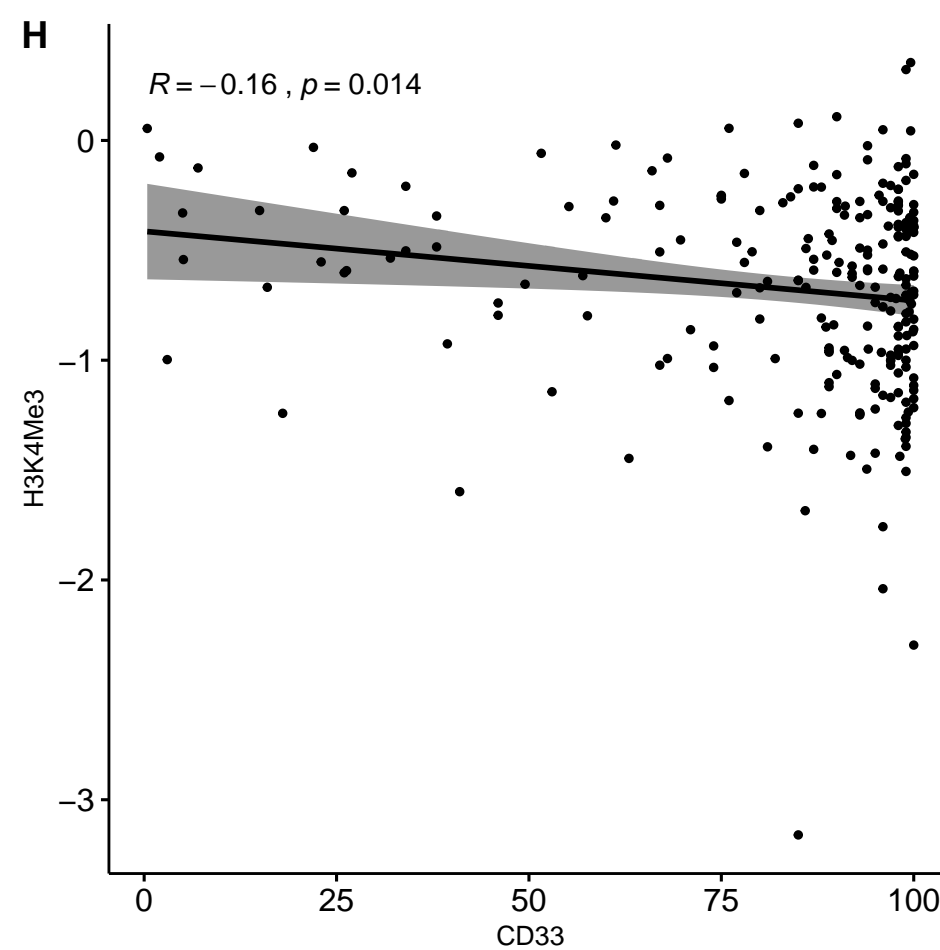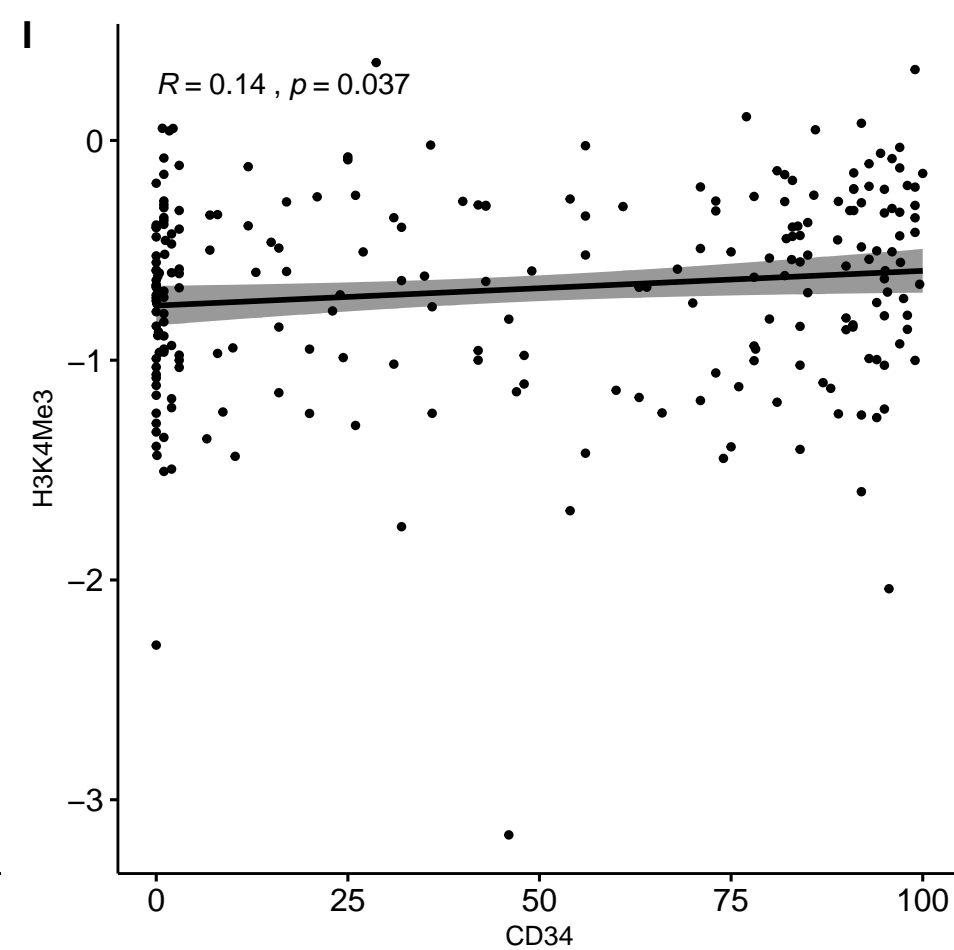

Supplement: Supplementary file 3 — Additional file 3: Figure S3. Relative quantity of H3K4Me3 in peripheral blood and bone marrow samples obtained from acute myeloid leukemia patients was a significantly higher in patients who received prior chemotherapy (n = 23) compared to those who did not (n = 217, Wilcoxon, p = 0.019); b significantly higher in patients who received prior radiotherapy (n = 21) compared to those who did not (n = 219, Wilcoxon, p = 0.043). H3K4Me3 and c significantly lower in patients with the FLT3-D835 mutation (n = 19) compared to those who had FLT3-D835 wildtype (n = 204, Wilcoxon, p = 0.029). d H3K4Me3 negatively correlated with the percentage of monocytes in the bone marrow (r = − 0.15, p = 0.017); e the percentage of monocytes in the peripheral blood (r = − 0.19, p < 0.01); f and the platelet count (r = − 0.15, p = 0.019); g H3K4Me3 positively correlated with the presence of the surface marker CD13 (r = 0.13, p = 0.043); h H3K4Me3 negatively correlated with the presence of the surface marker CD33 (r = − 0.16, p = 0.014); i H3K4Me3 positively correlated with the presence of the surface marker CD34 (r = 0.14, p = 0.037). [file 13148_2021_1011_MOESM3_ESM.pdf]

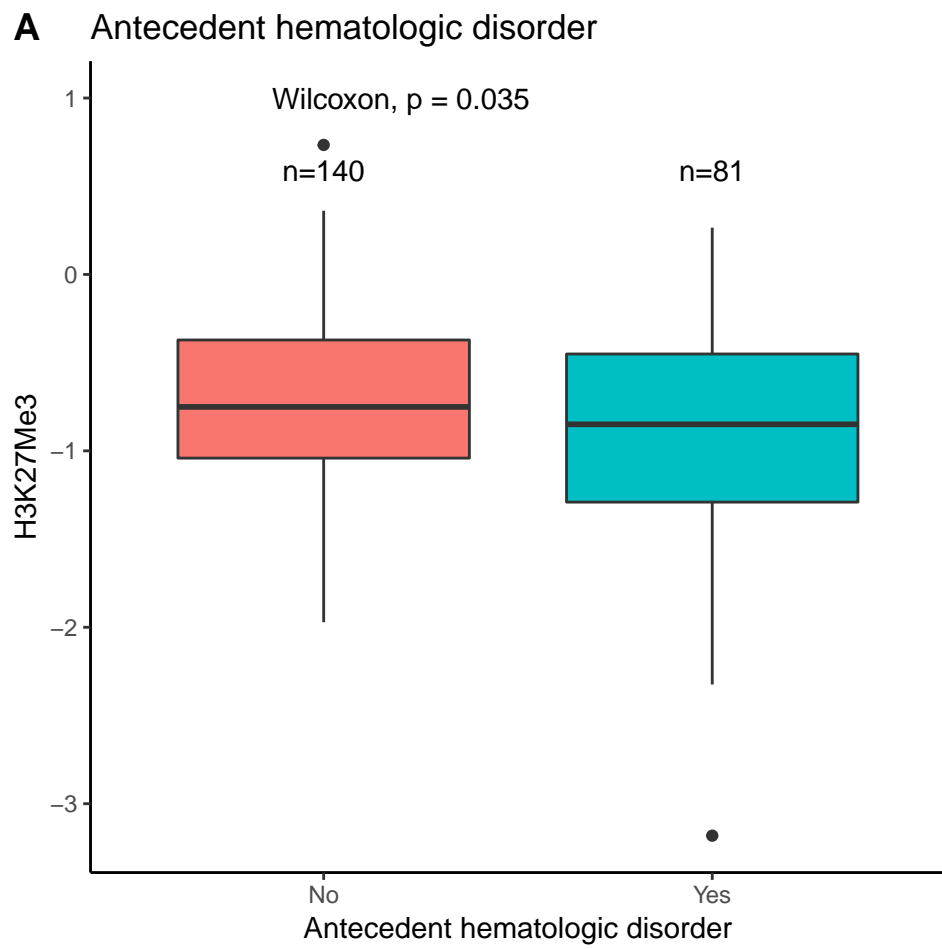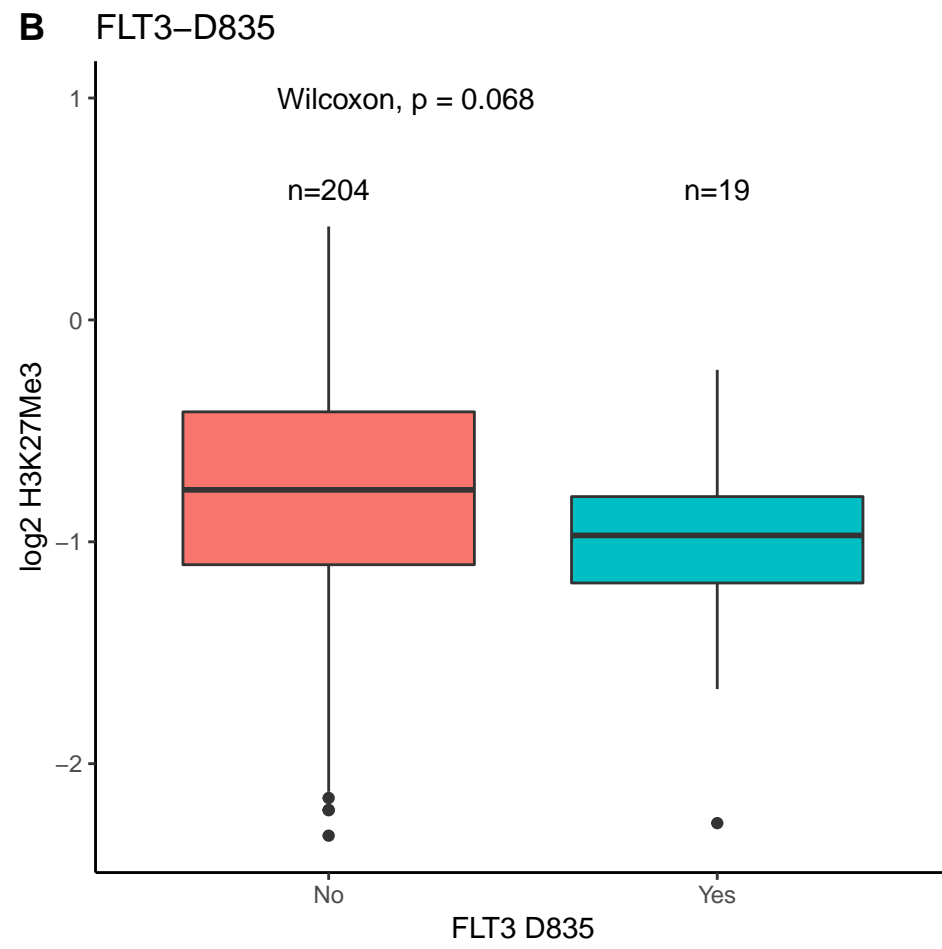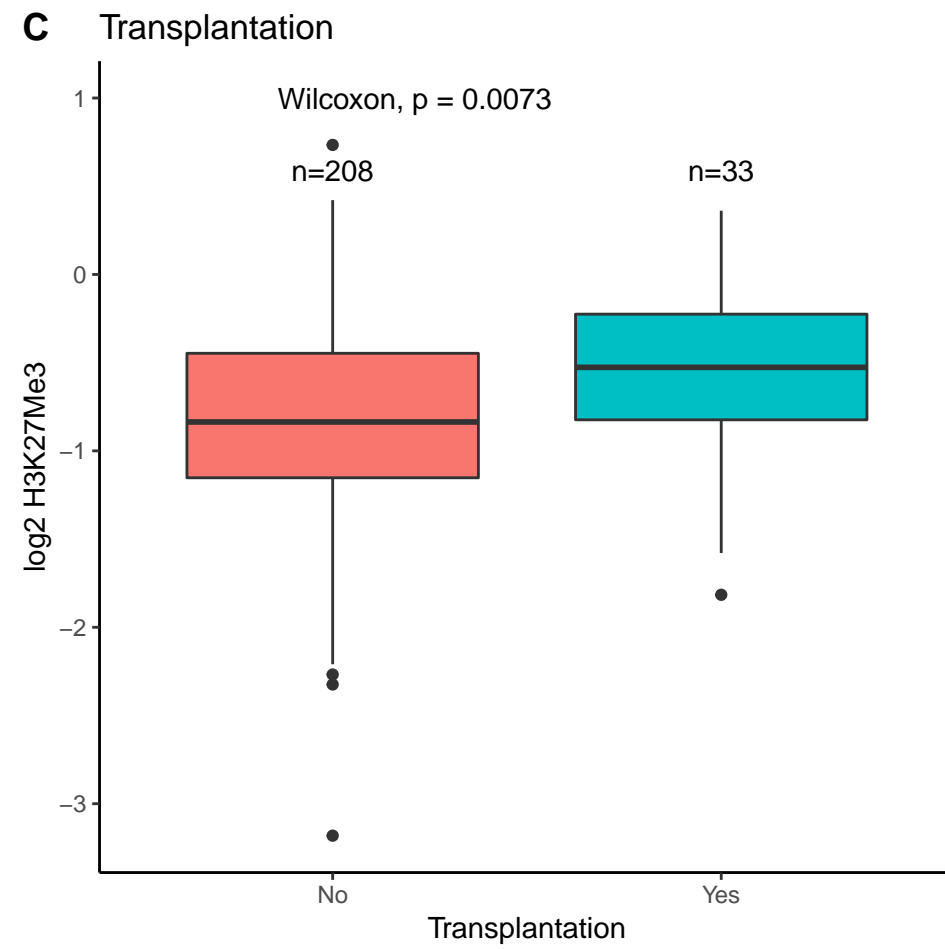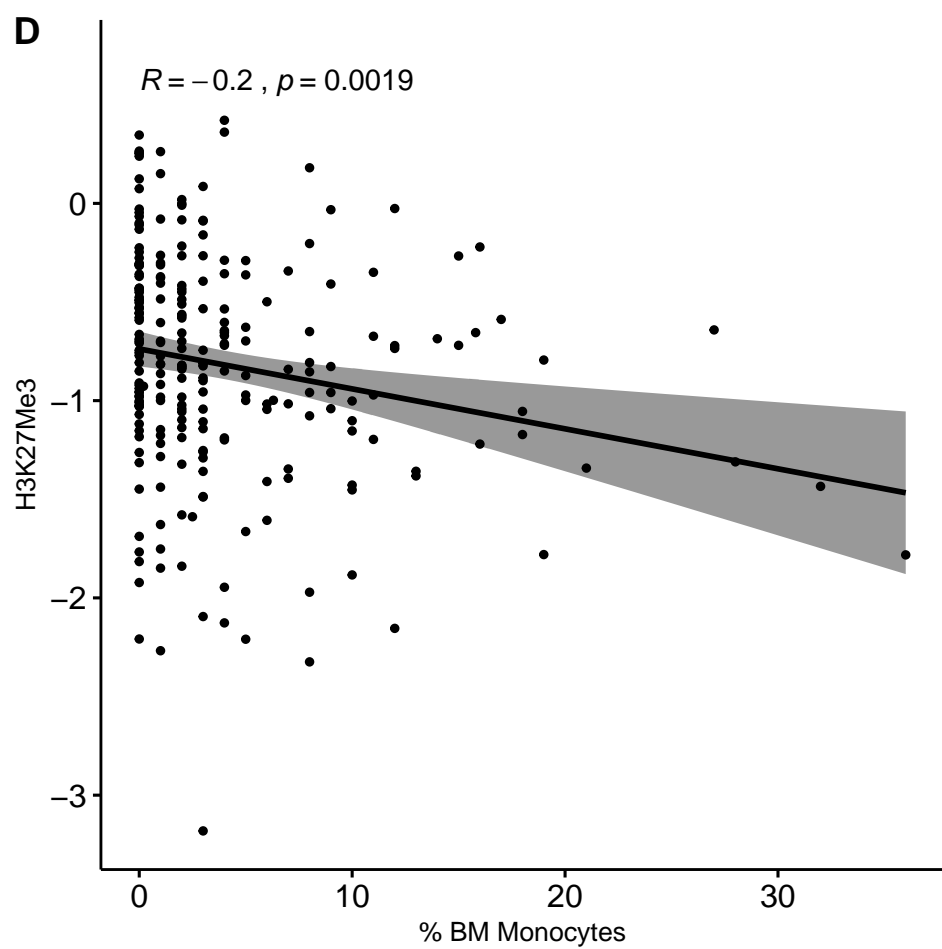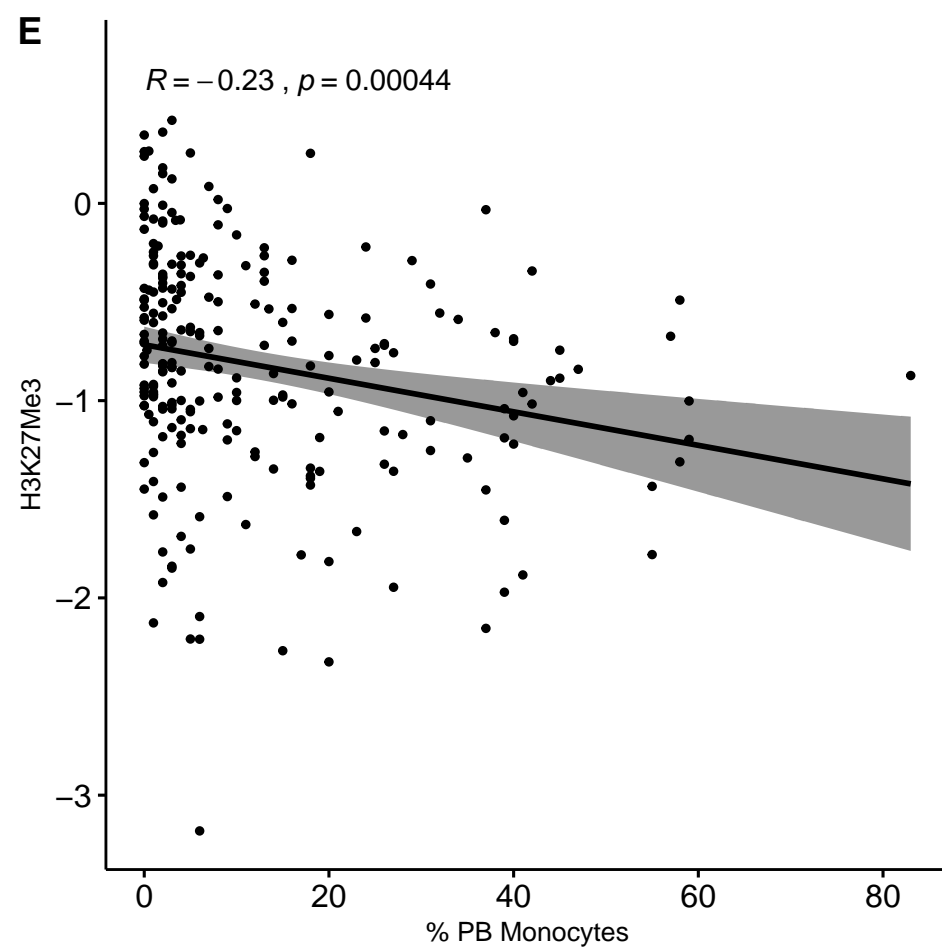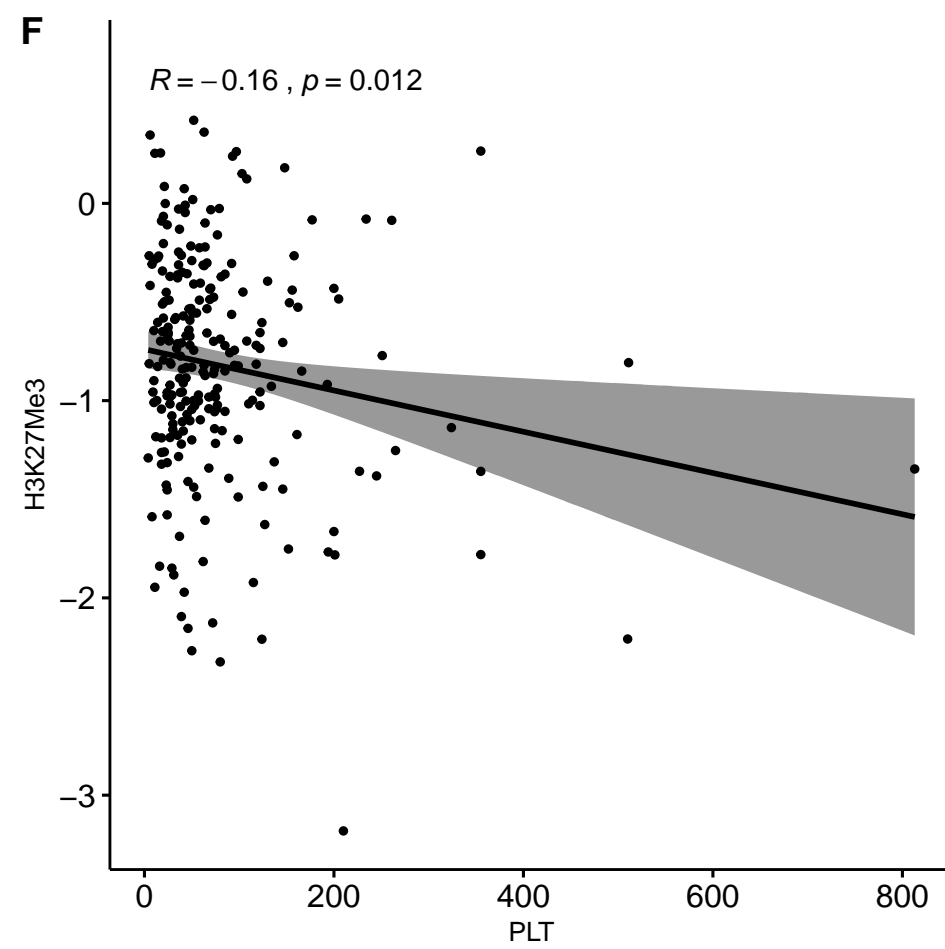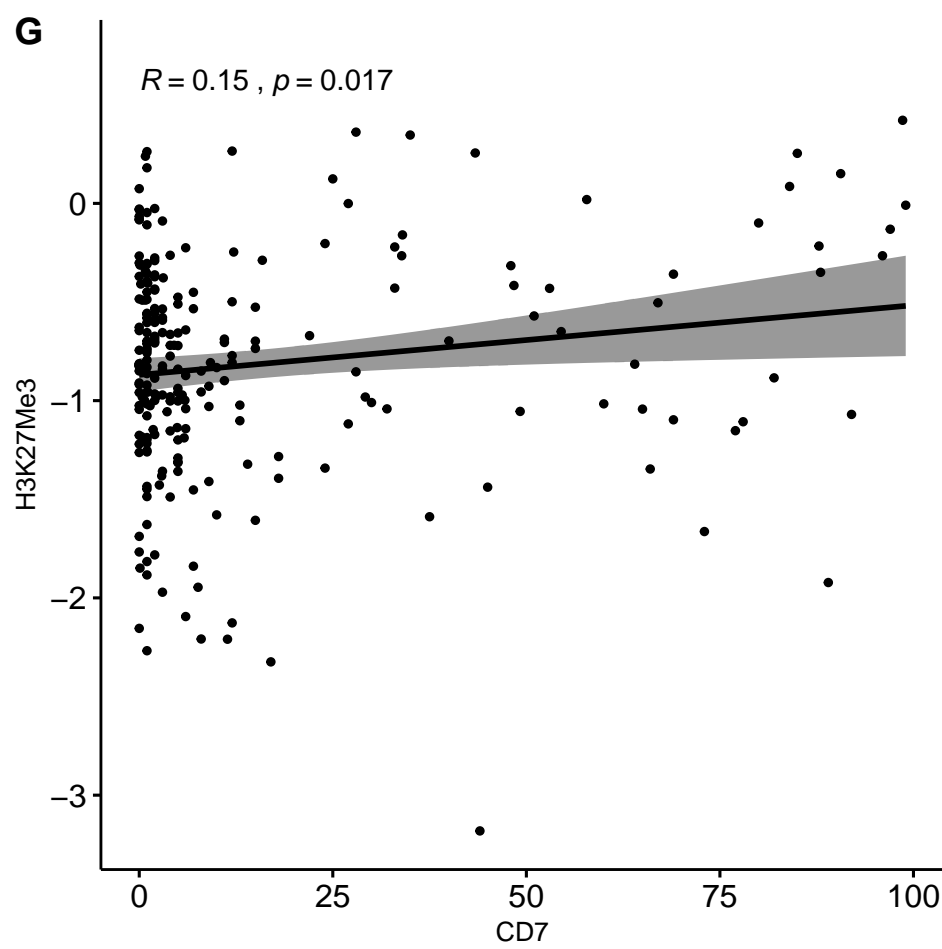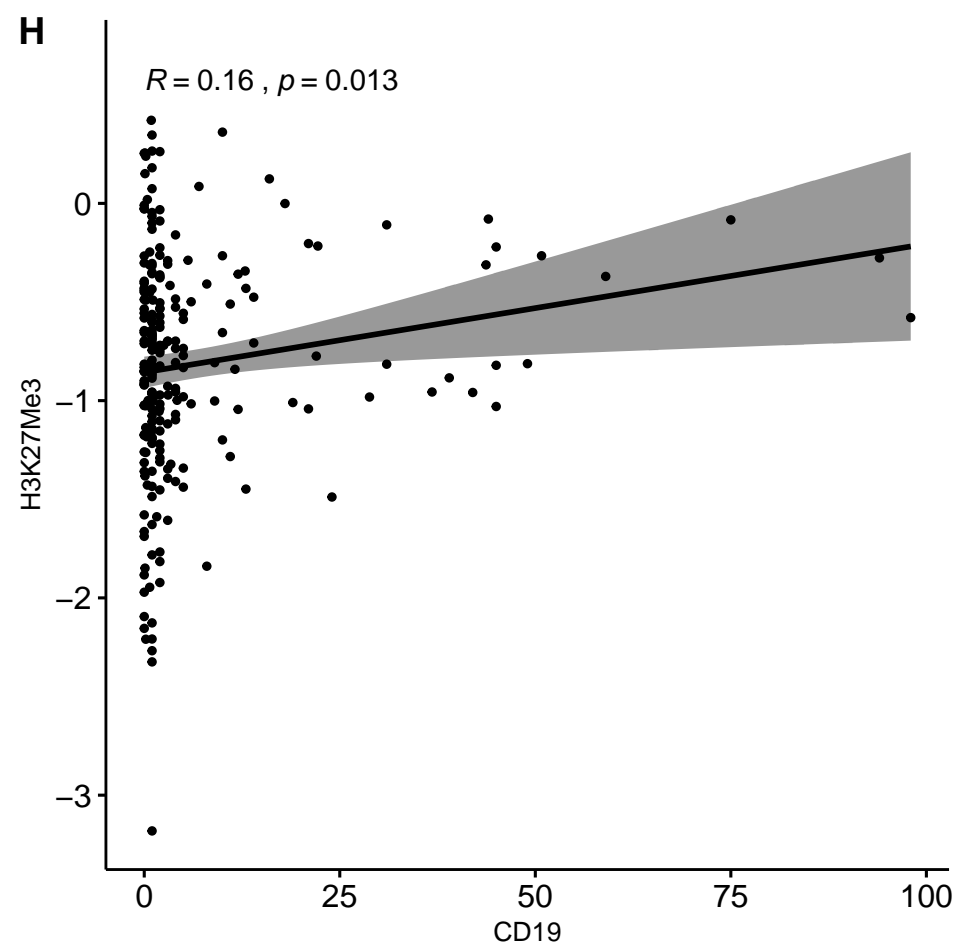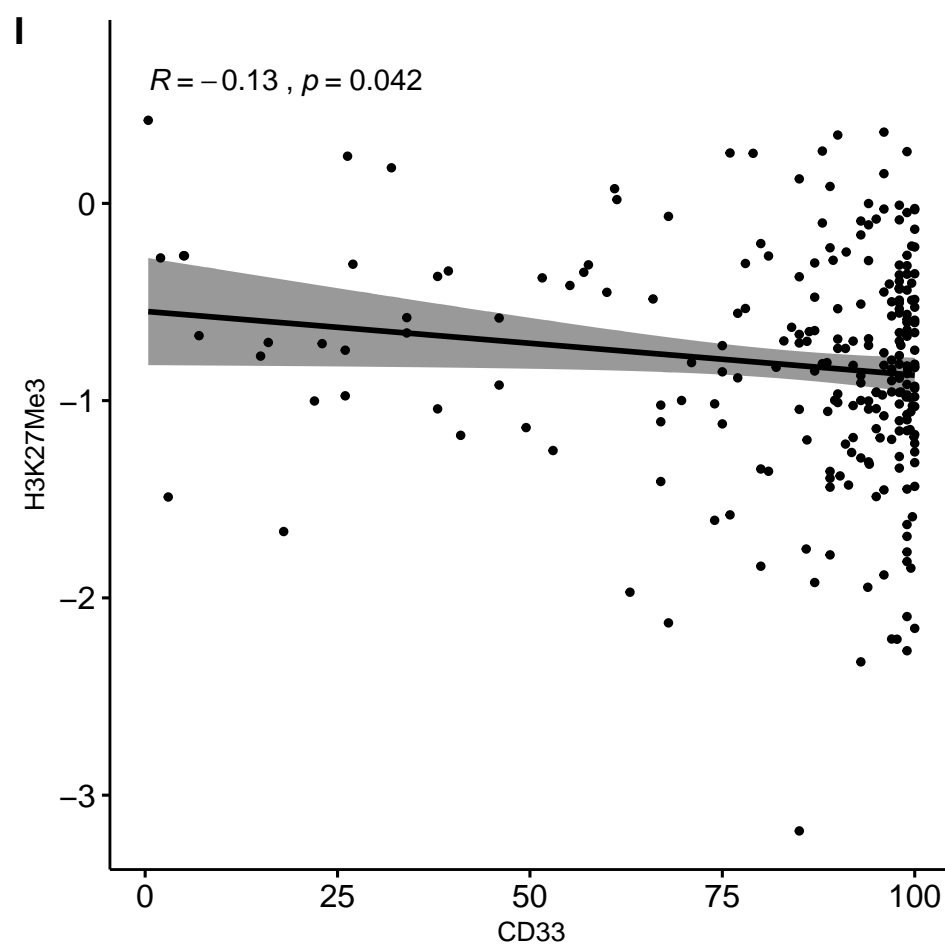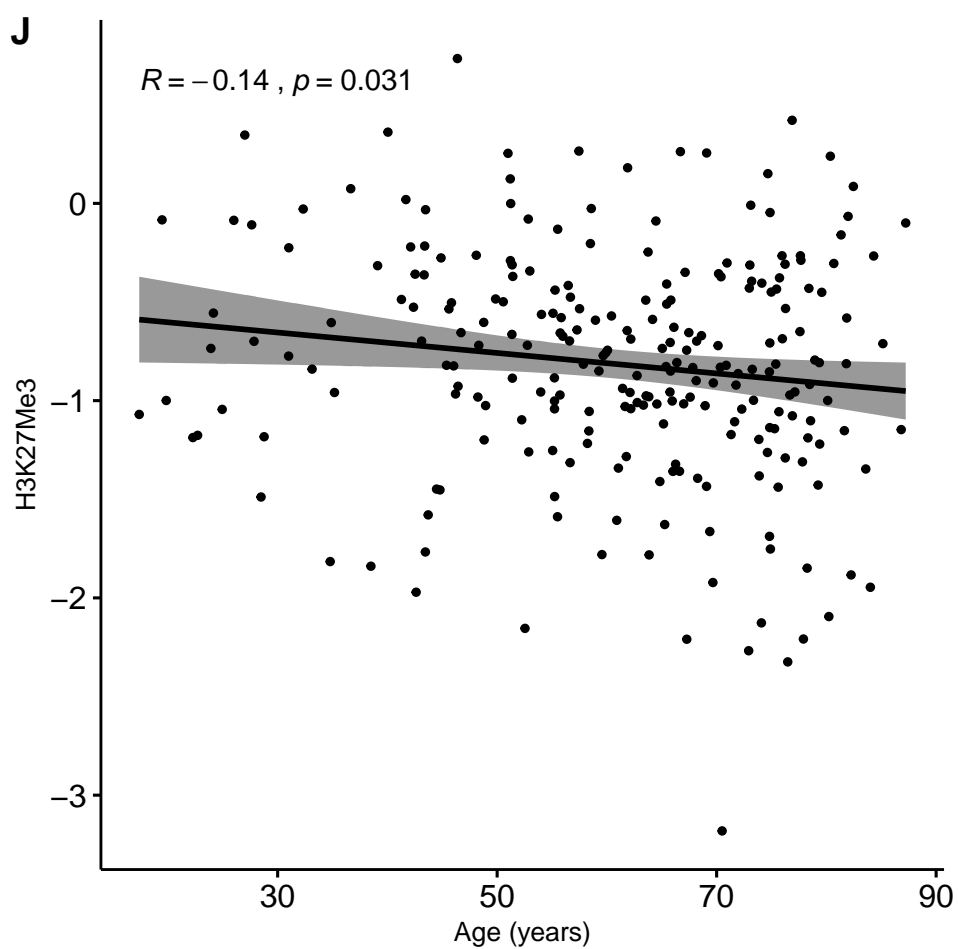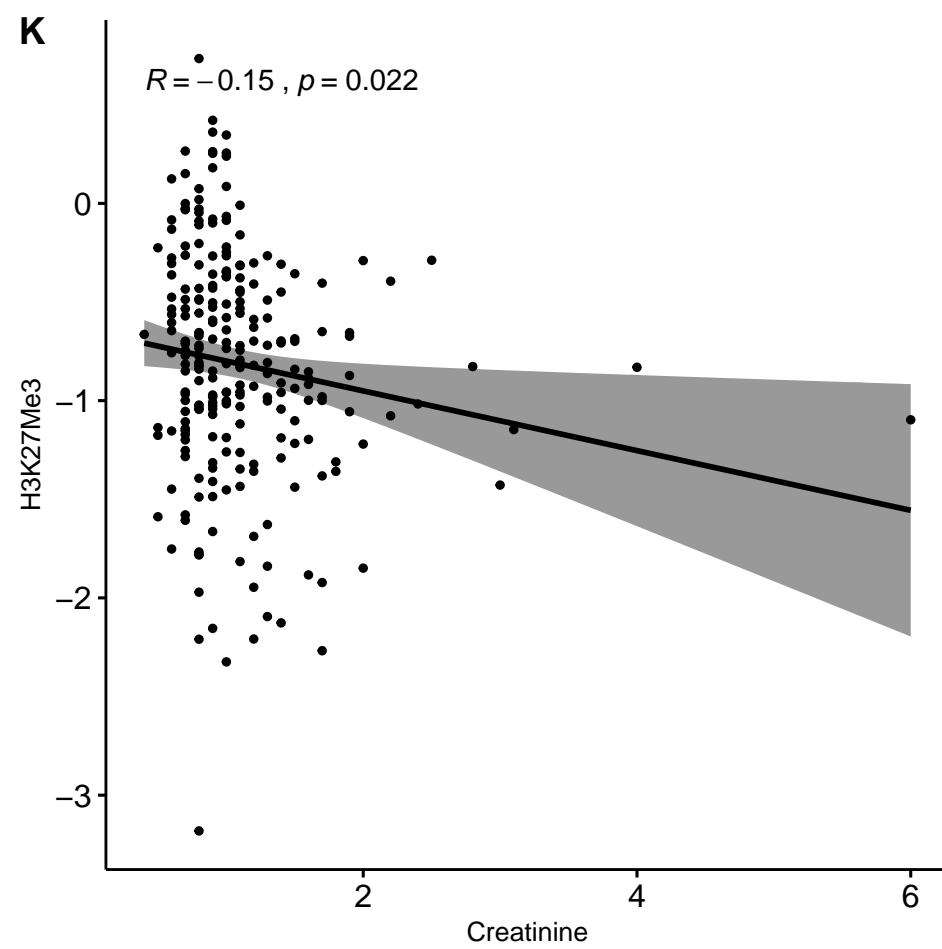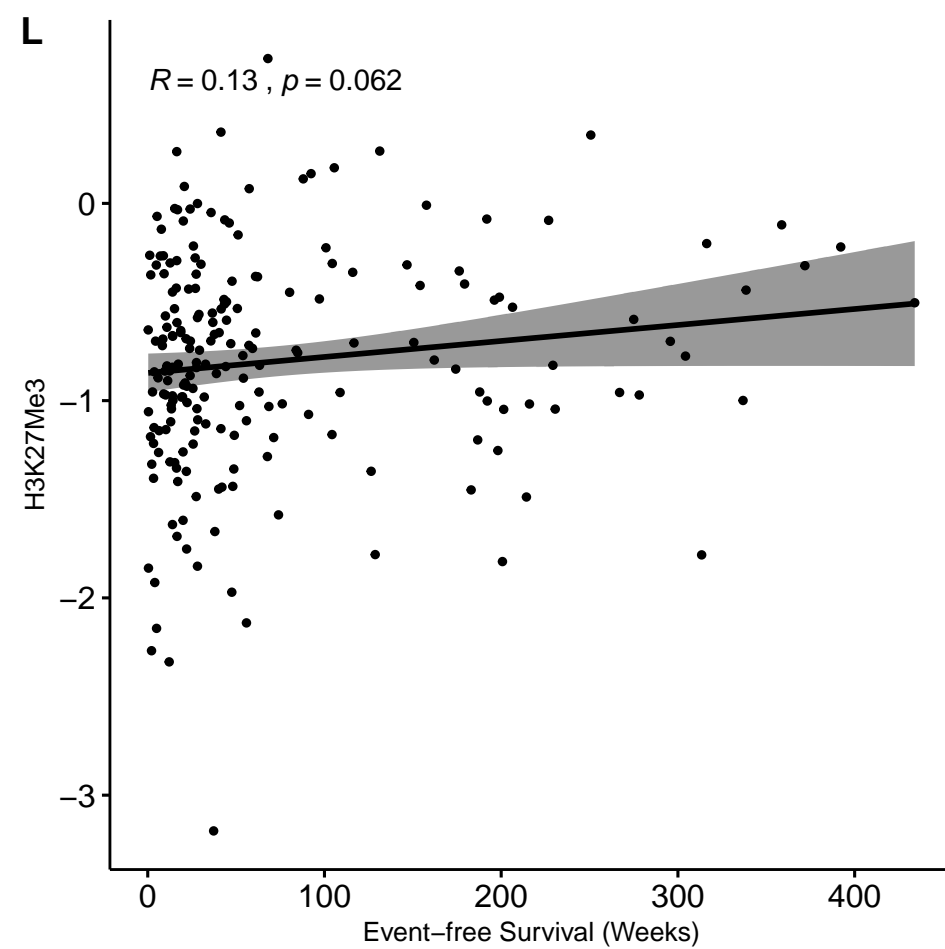

Supplement: Supplementary file 4 — Additional file 4: Figure S4. Relative quantity of H3K27Me3 in peripheral blood and bone marrow samples obtained from acute myeloid leukemia patients was a significantly lower in patients who experienced antecedent hematologic disorder (n = 81) compared to those without (n = 140, Wilcoxon, p = 0.035); b lower in patients with the FLT3-D835 mutation (n = 19) compared to those without (n = 204, Wilcoxon, p = 0.068) and c significantly higher in patients who received a transplantation (n = 233) compared to the ones that were not transplanted (n = 208, Wilcoxon, p < 0.01). d H3K27Me3 negatively correlated with the percentage of monocytes in the bone marrow (r = − 0.20, p < 0.0); e the percentage of monocytes in the peripheral blood (r = − 0.23, p < 0.01); f and the platelet count (r = − 0.16, p = 0.012); g H3K27Me3 positively correlated with the presence of the surface marker CD7 (r = 0.15, p = 0.017); h CD19 (r = 0.16, p = 0.013) i H3K27Me3 negatively correlated with the presence of the surface marker CD33 (r = − 0.13, p = 0.042); j age (r = − 0.14, p = 0.031); k creatinine (r = − 0.15, p = 0.022); l H3K27Me3 positively correlated with event-free survival duration (r = 0.13, p = 0.062). [file 13148_2021_1011_MOESM4_ESM.pdf]

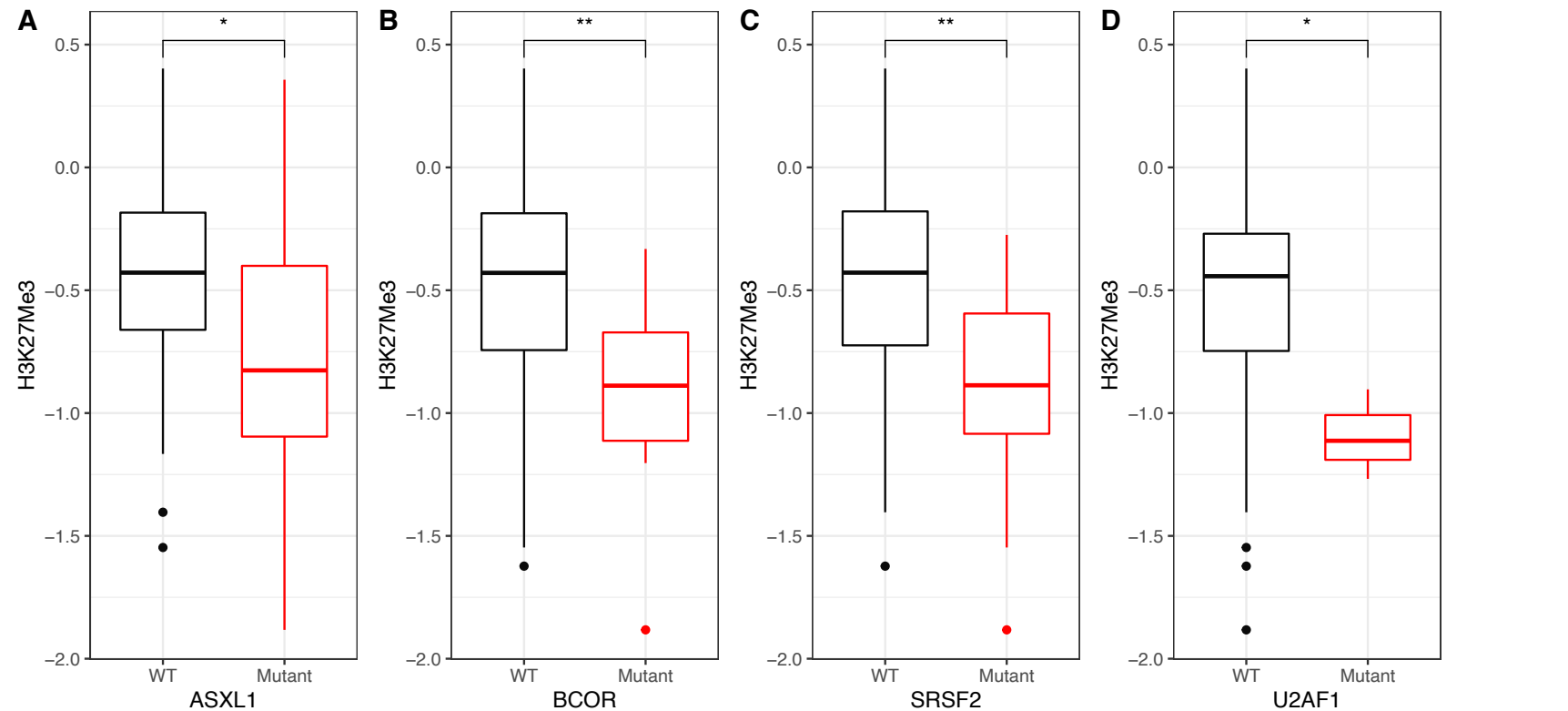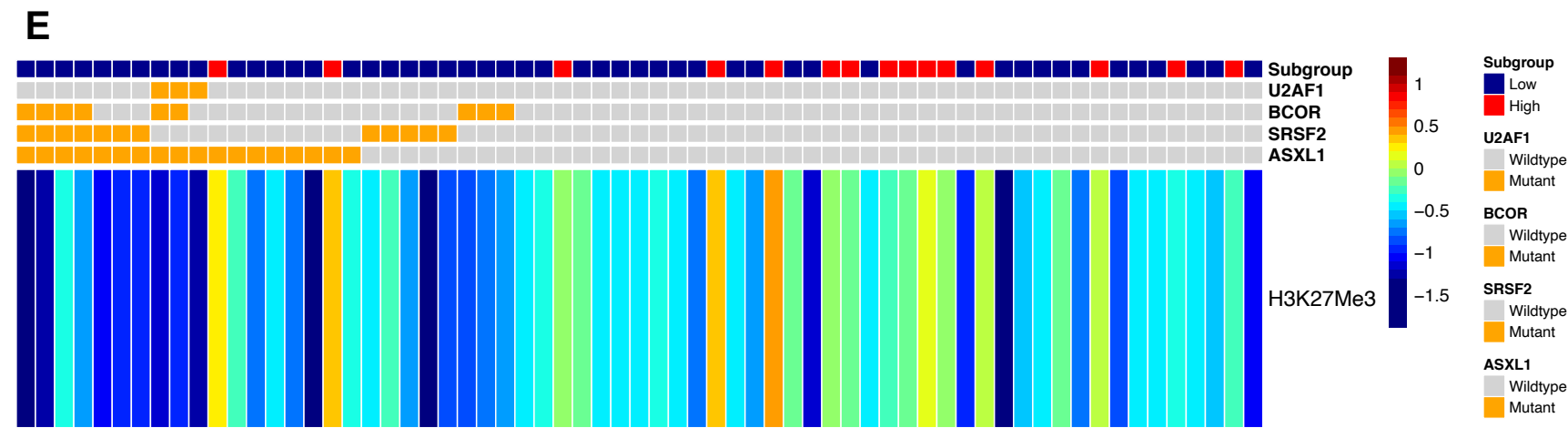

Supplement: Supplementary file 9 — Additional file 9: Figure S5. H3K27Me3 is significantly lower in a ASXL1 b BCOR c SRSF2 d U2AF1 mutated patients with acute myeloid leukemia (AML) compared to wildtype. *p < 0.05, ** p < 0.01. e Heatmap showing relative H3K27Me3 level per sample of 65 sequenced bone marrow aspirates from AML patients along with the presence (orange) or absence (gray) of ASXL1, BCOR, SRSF2 and/or U2AF1 mutations and subgroup H3K27Me3; H3K27Me3low in blue and H3K27Me3high in red. Patients with denoted mutations had lower levels H3K27Me3. [file 13148_2021_1011_MOESM9_ESM.pdf]

A

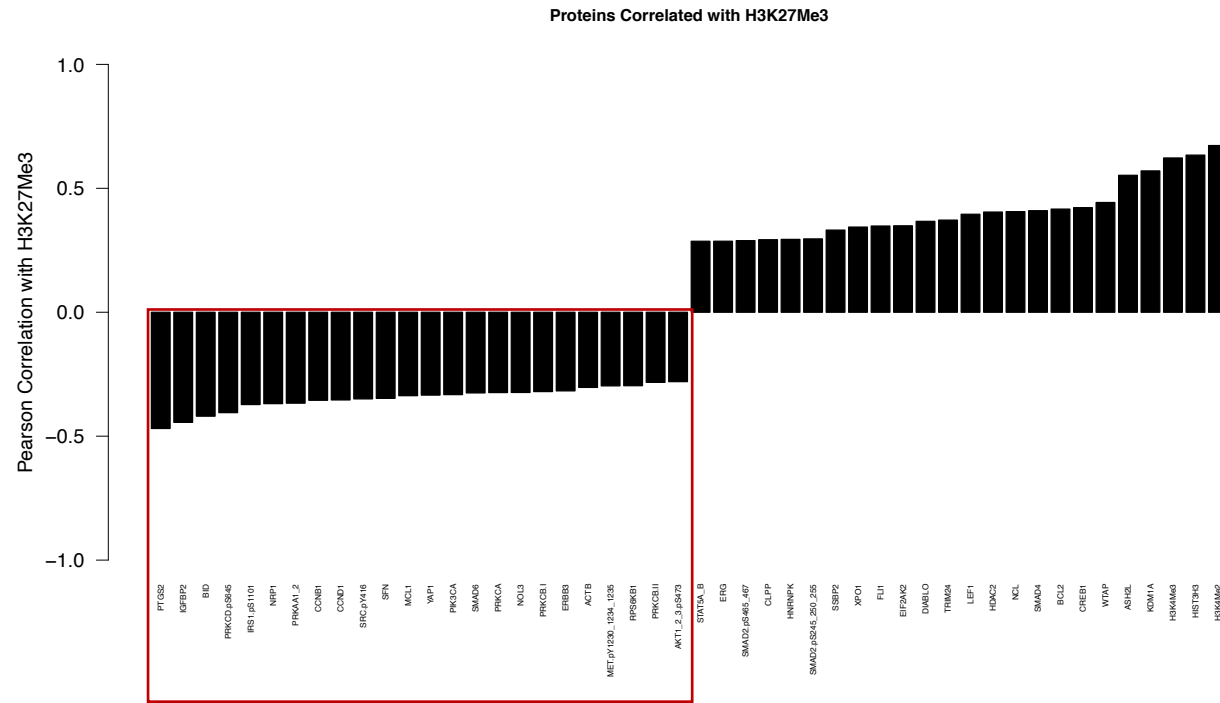

B

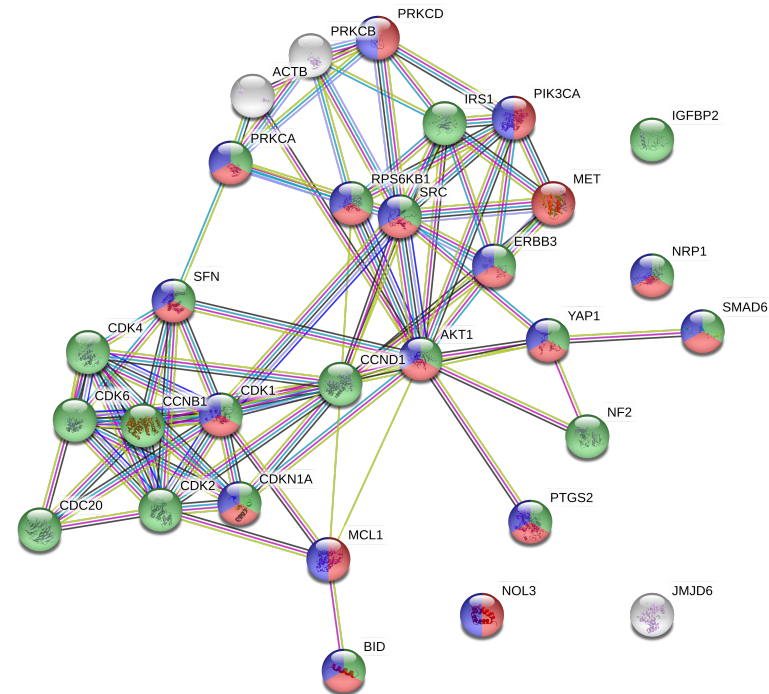

Supplement: Supplementary file 10 — Additional file 10: Figure S6. a Waterfall plot showing significant correlated protein expressions between H3K27Me3 and the other 229 antibodies on the reverse phase protein array (RPPA) identified positively and negatively associated proteins. Negatively correlated proteins (Pearson correlation R > 0.25, p < 0.0001) that are supposed to be upregulated if H3K27Me3 was lost are red-lined. b String analysis reveals H3K27Me3 negatively associated proteins are interconnected in shared biological processes; in red: Negative regulation of programmed cell death (GO:0043069, FDR = 1.27e−14), in green: Regulation of cell population proliferation (GO:0042127, FDR = 1.31e − 14) and in blue: Negative regulation of apoptotic process (GO:0043066, FDR = 8.42e−14). [file 13148_2021_1011_MOESM10_ESM.pdf]

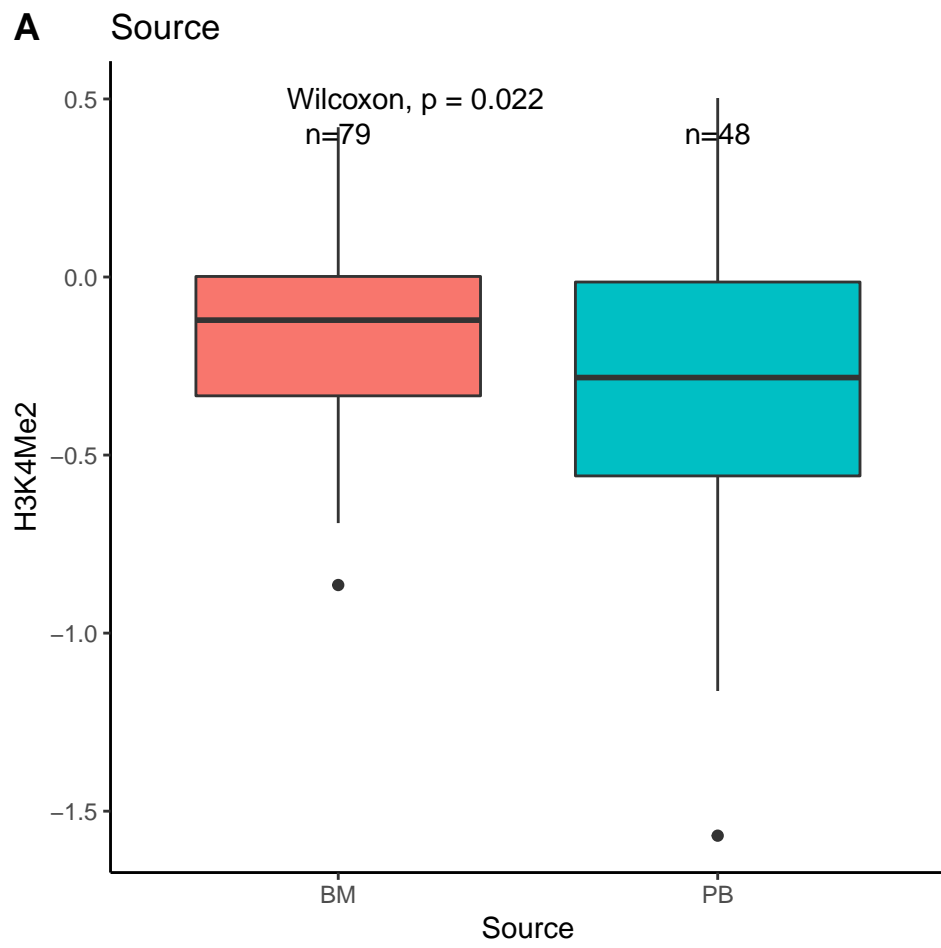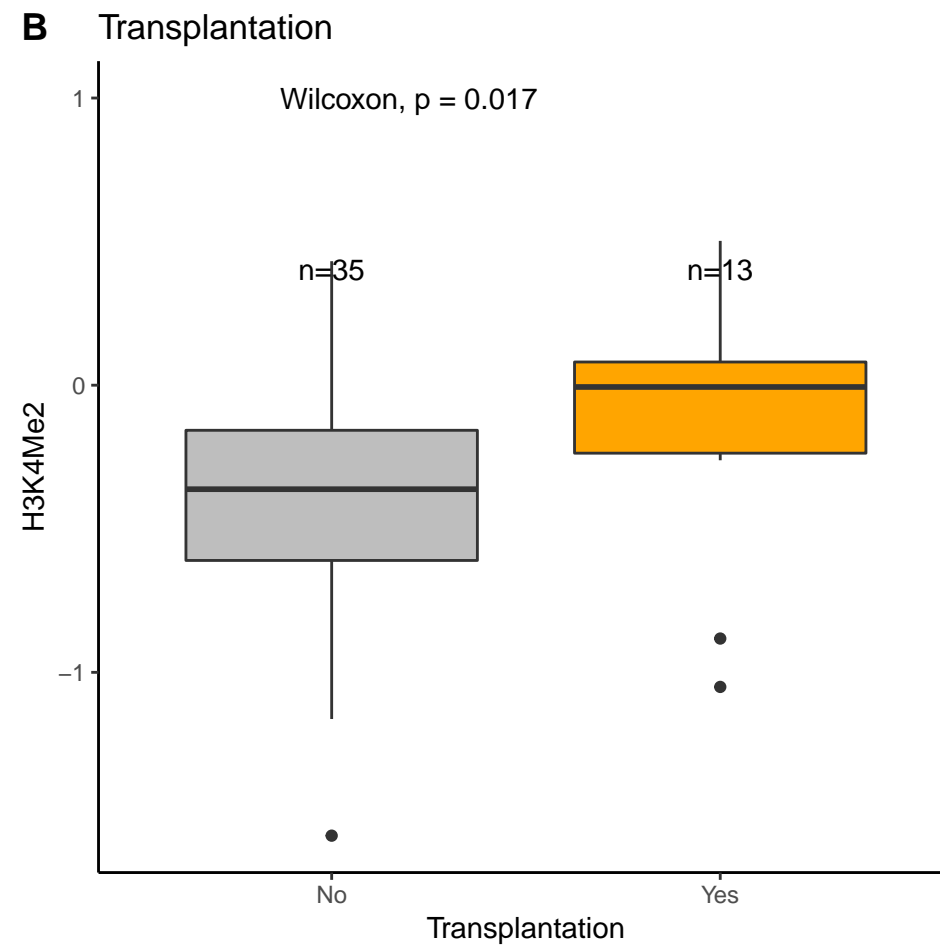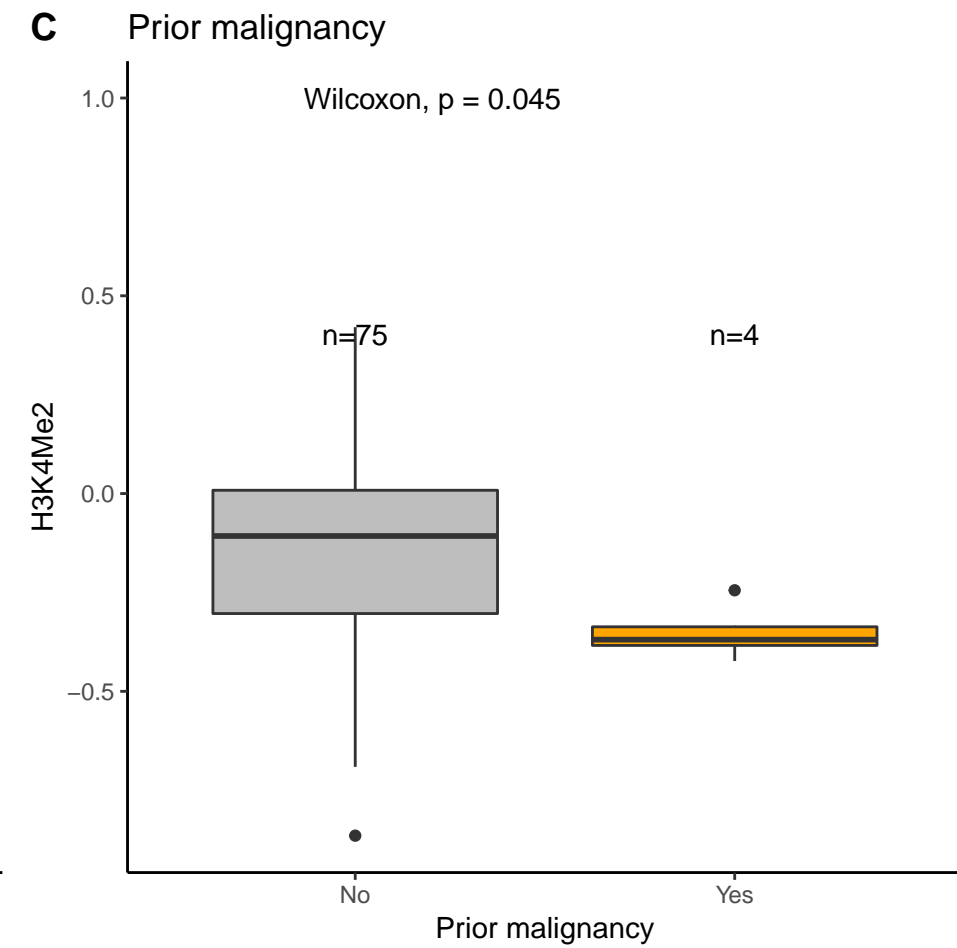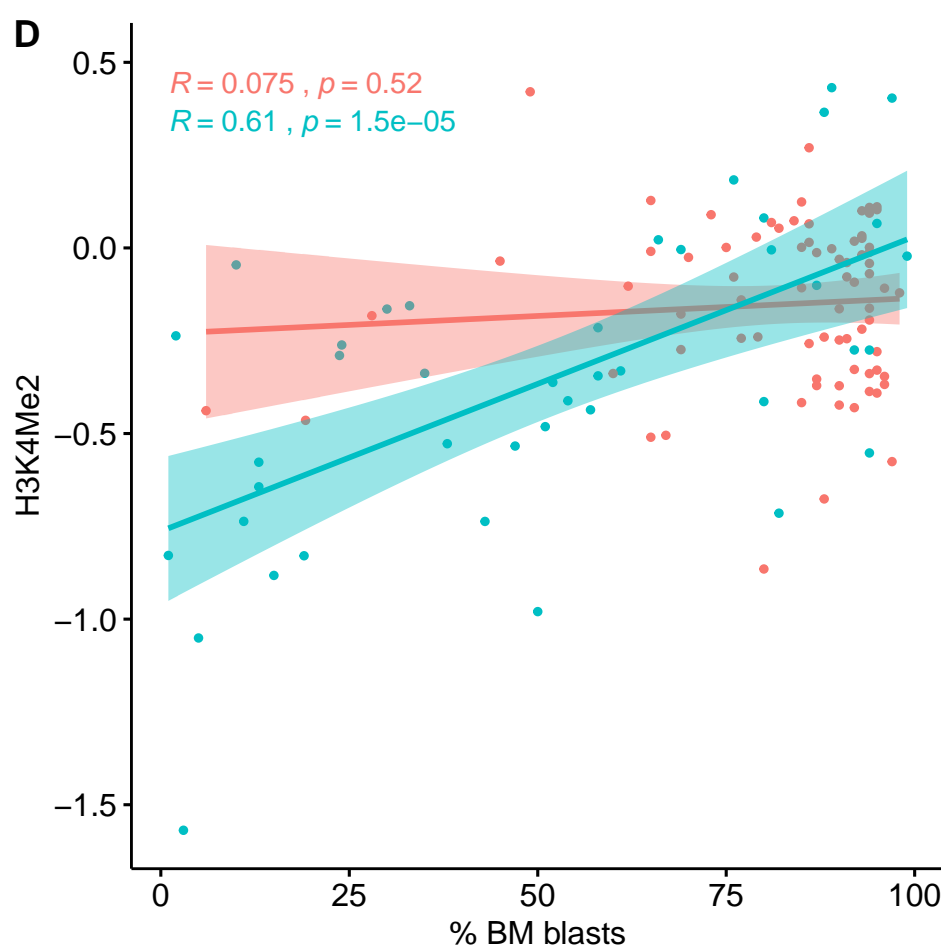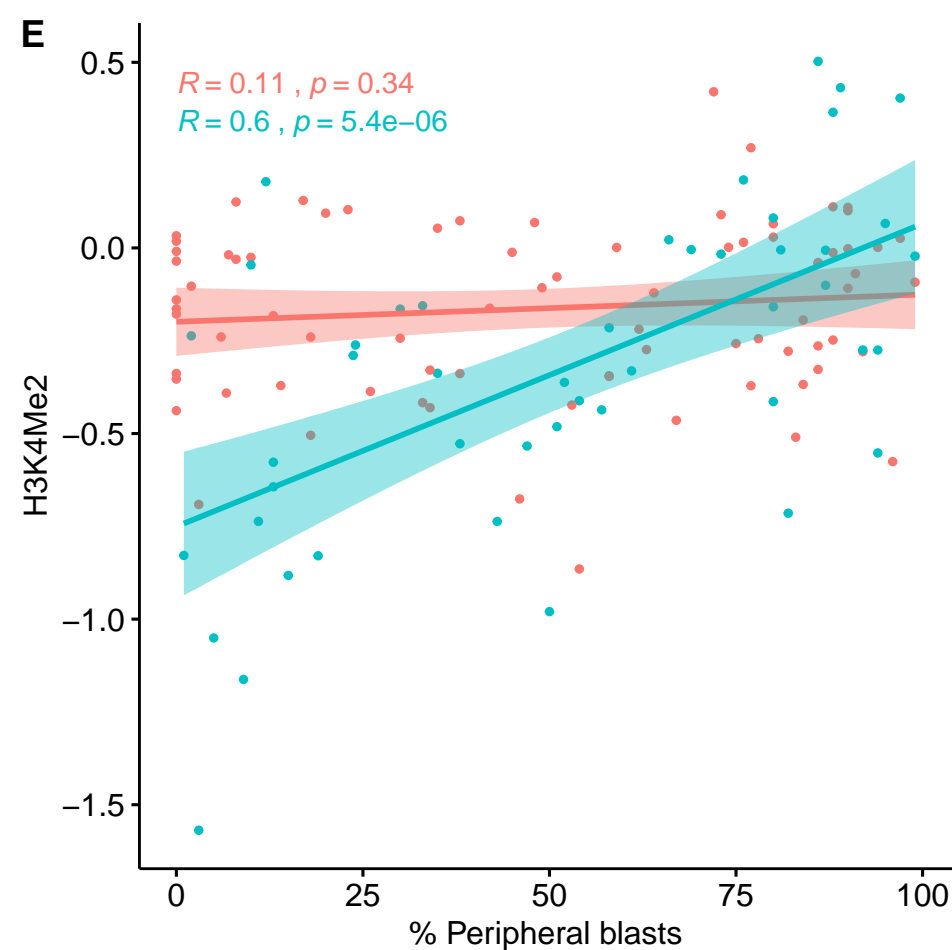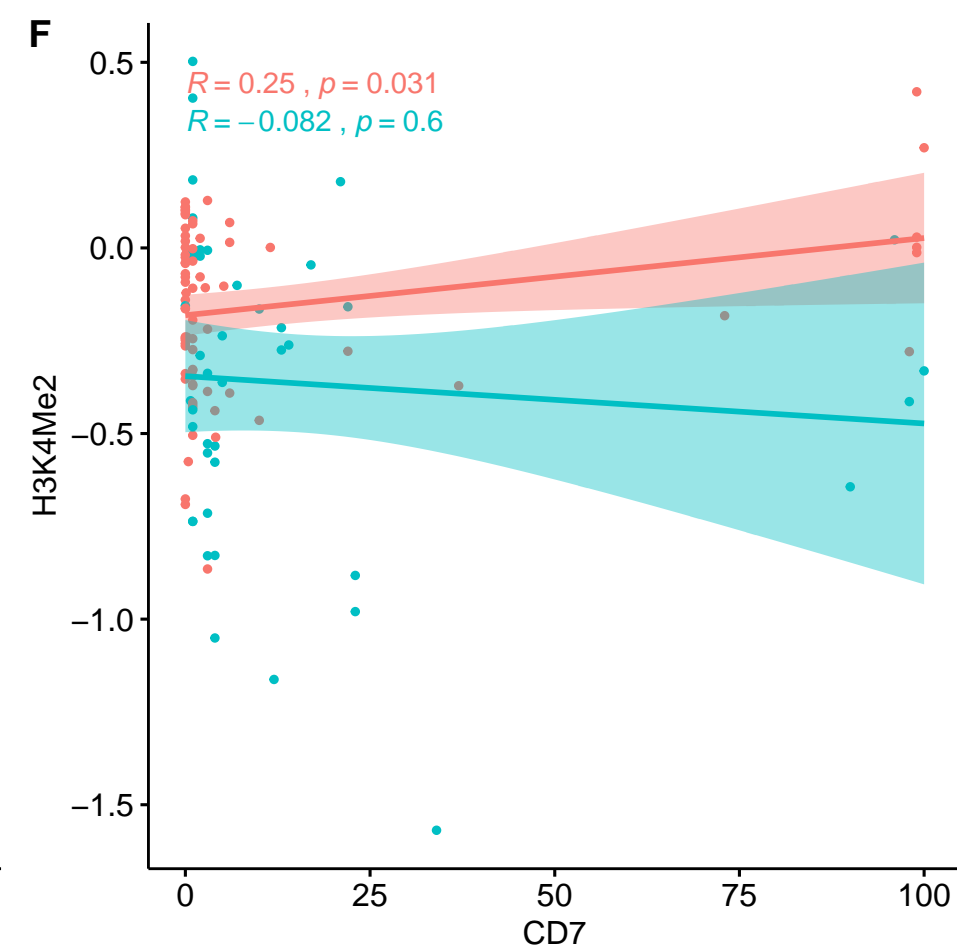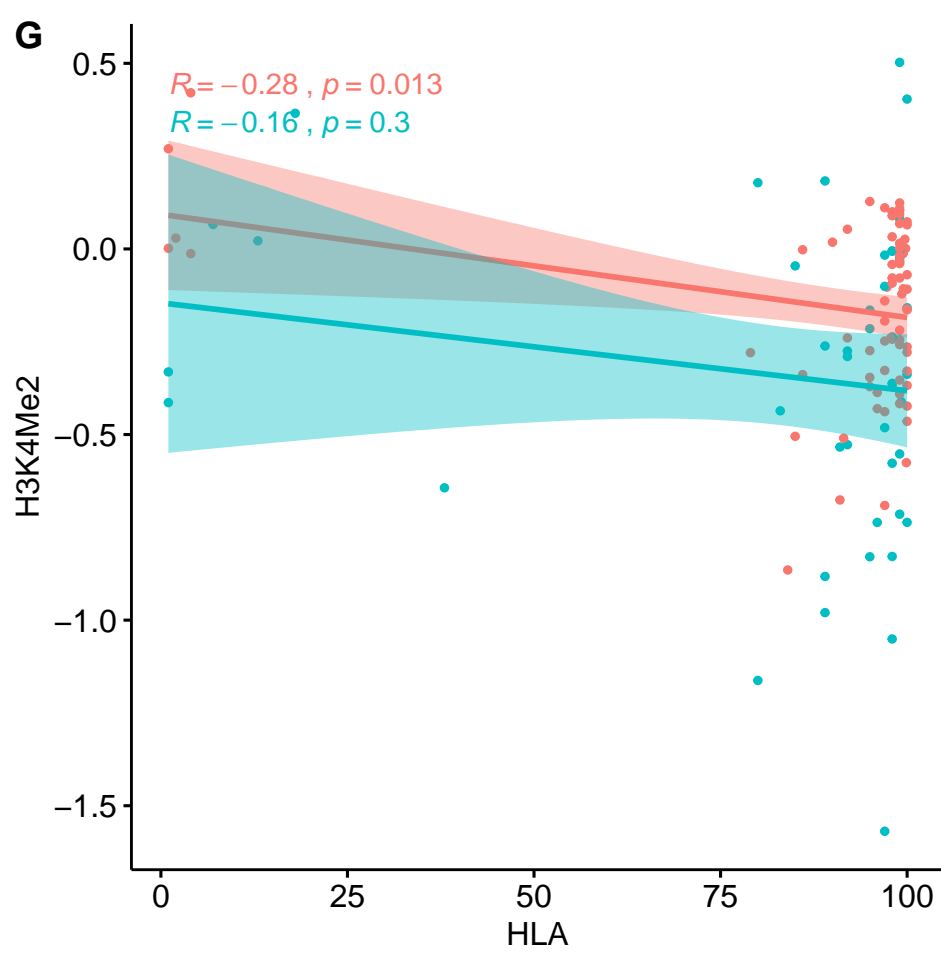

Supplement: Supplementary file 12 — Additional file 12: Figure S7. Higher expression levels of H3K4Me2 in a bone marrow (BM, n = 79) samples compared to peripheral blood (PB, n = 48) obtained from acute lymphoblastic leukemia patients (ALL, Wilcoxon, p = 0.022). b Higher H3K4Me2 in PB samples from ALL patients that underwent transplantation compared to their expression in PB samples from patients without (Wilcoxon, p = 0.017). c Lower H3K4Me2 was seen in 4 BM samples from patients that had a prior malignancy compared to H3K4Me2 level in BM from patients who had no prior malignancy (n = 75, Wilcoxon, p = 0.045). H3K4Me2 positively correlated with d the percentage of bone marrow blasts in BM samples (blue, r = 0.61, p < 0.01), but not in the PB samples (red). e The percentage peripheral blasts in BM samples (blue, r = 0.6, p < 0.01), but not in the PB samples (red). f H3K4Me2 positively correlated with the presence of the surface marker CD7 (r = 0.25, p = 0.031) in PB (red), but not in BM (blue); g H3K4Me2 negatively correlated with the presence of the surface marker HLA in the PB samples (r = − 0.28, p = 0.013), but not in the BM. [file 13148_2021_1011_MOESM12_ESM.pdf]

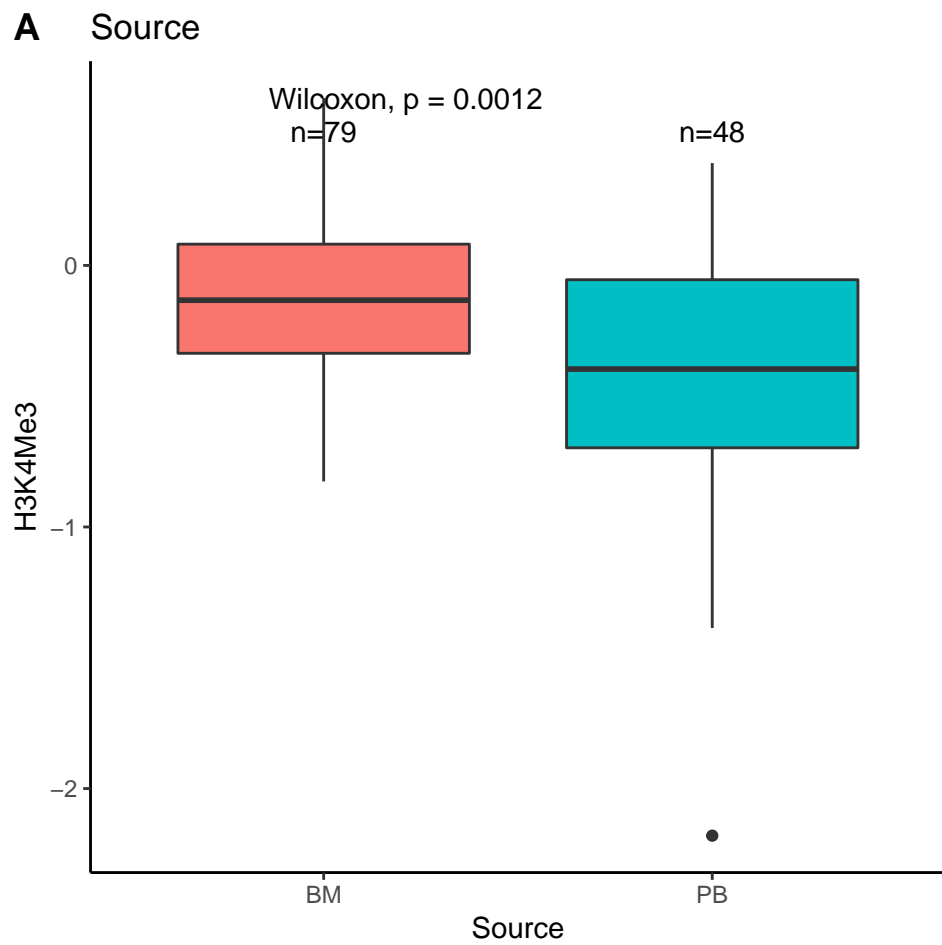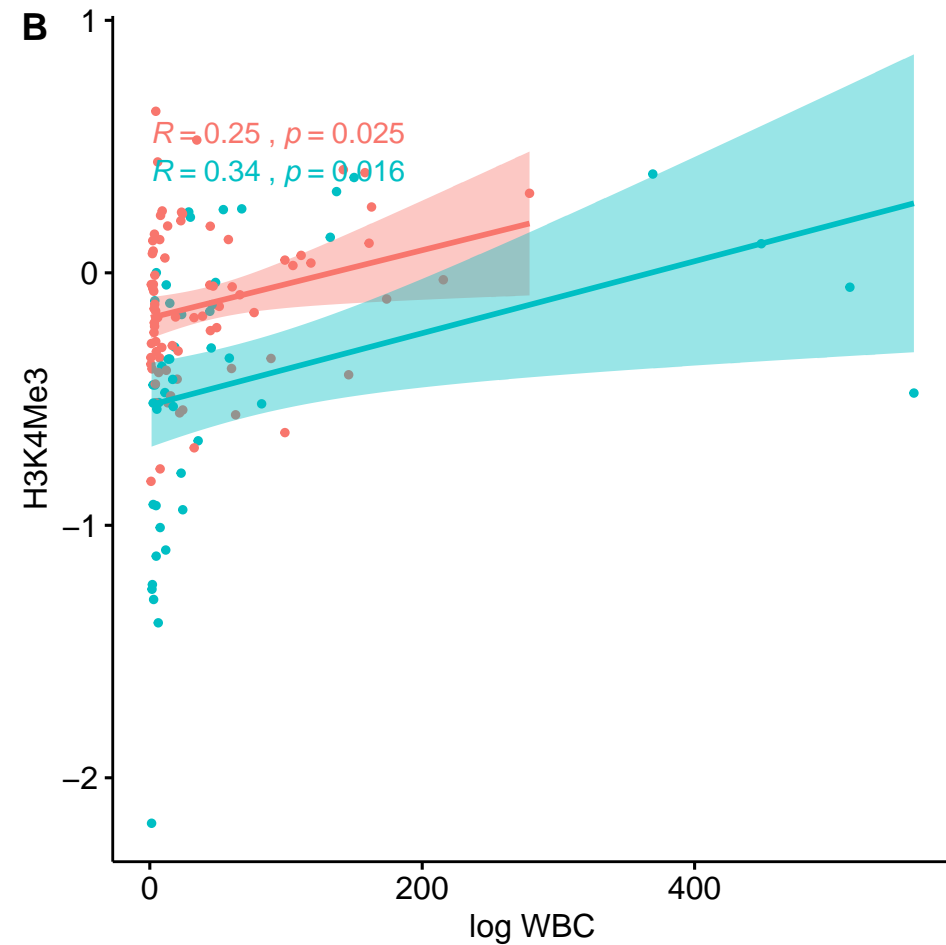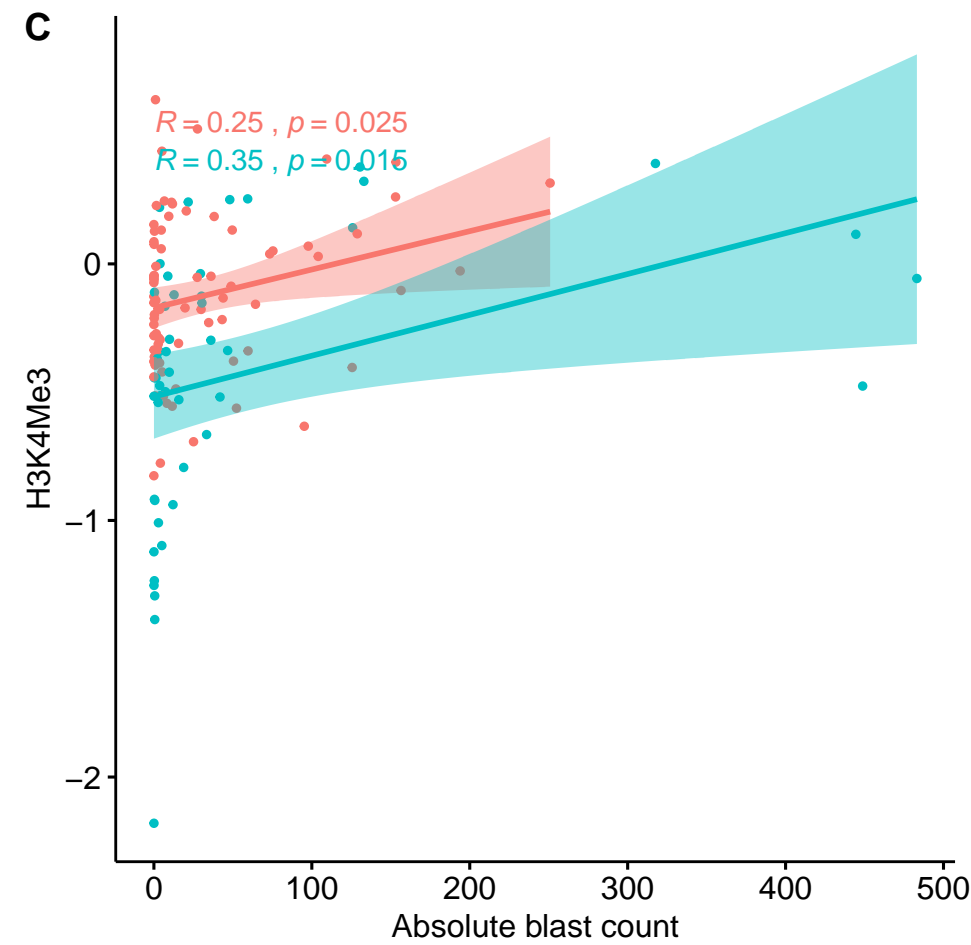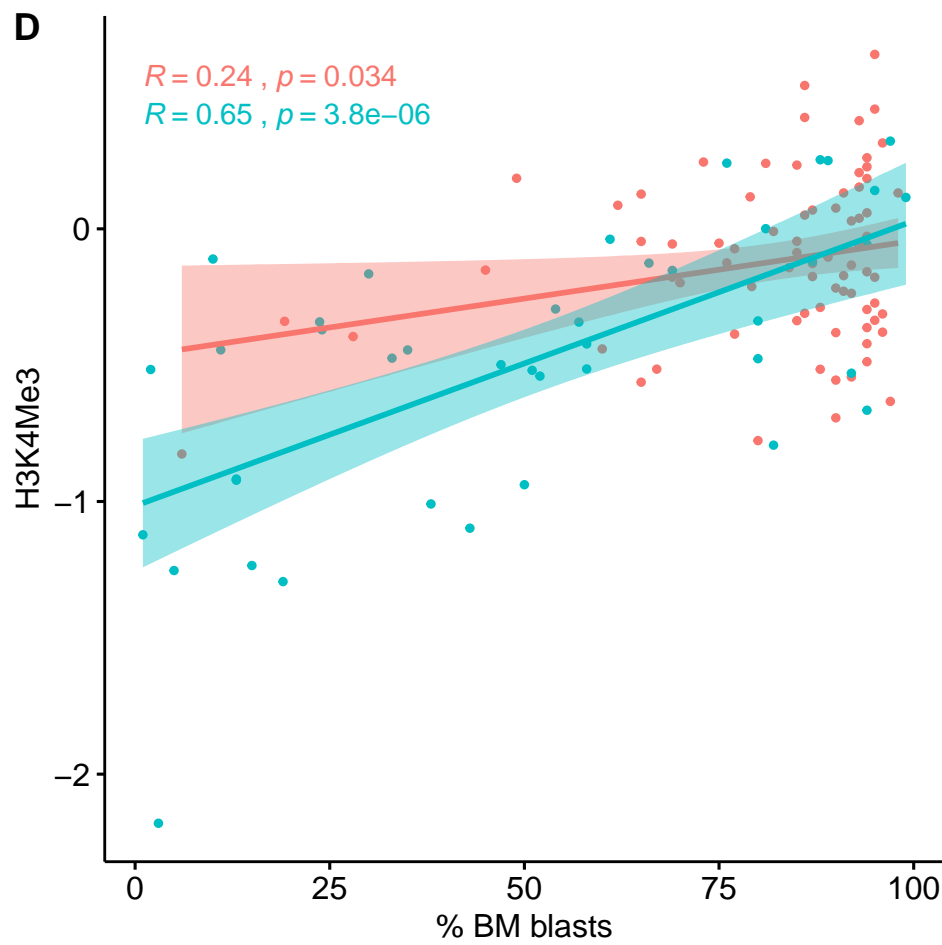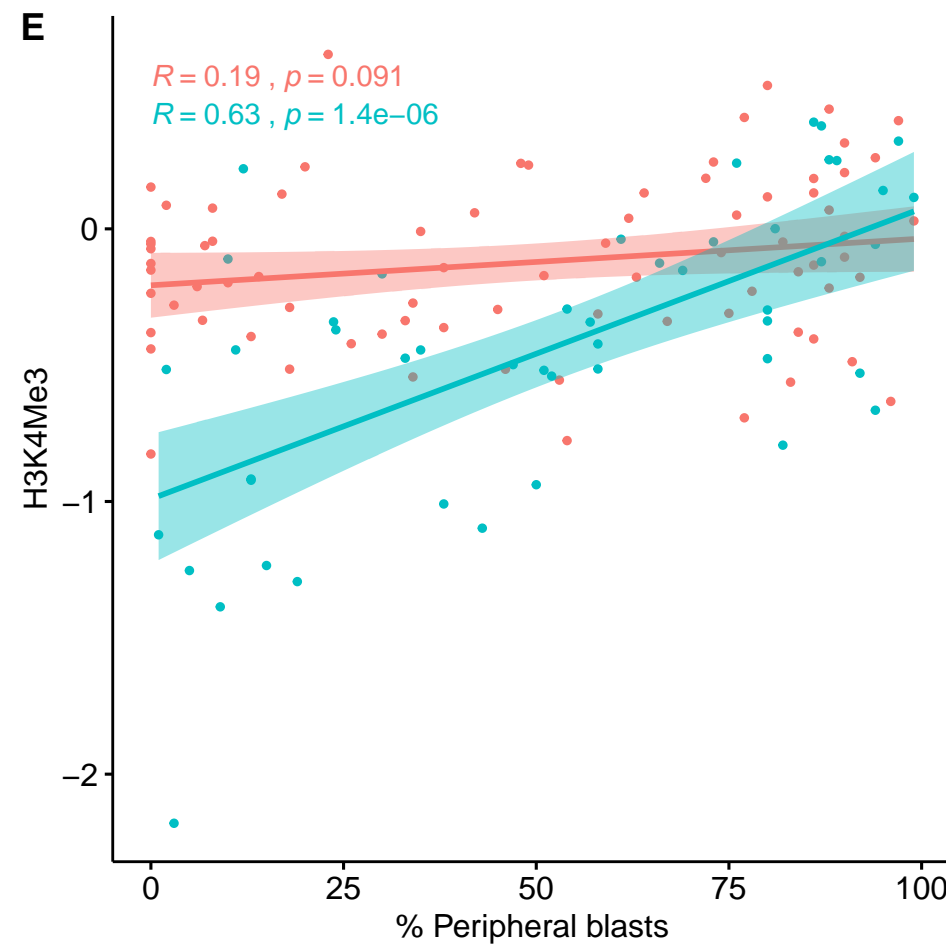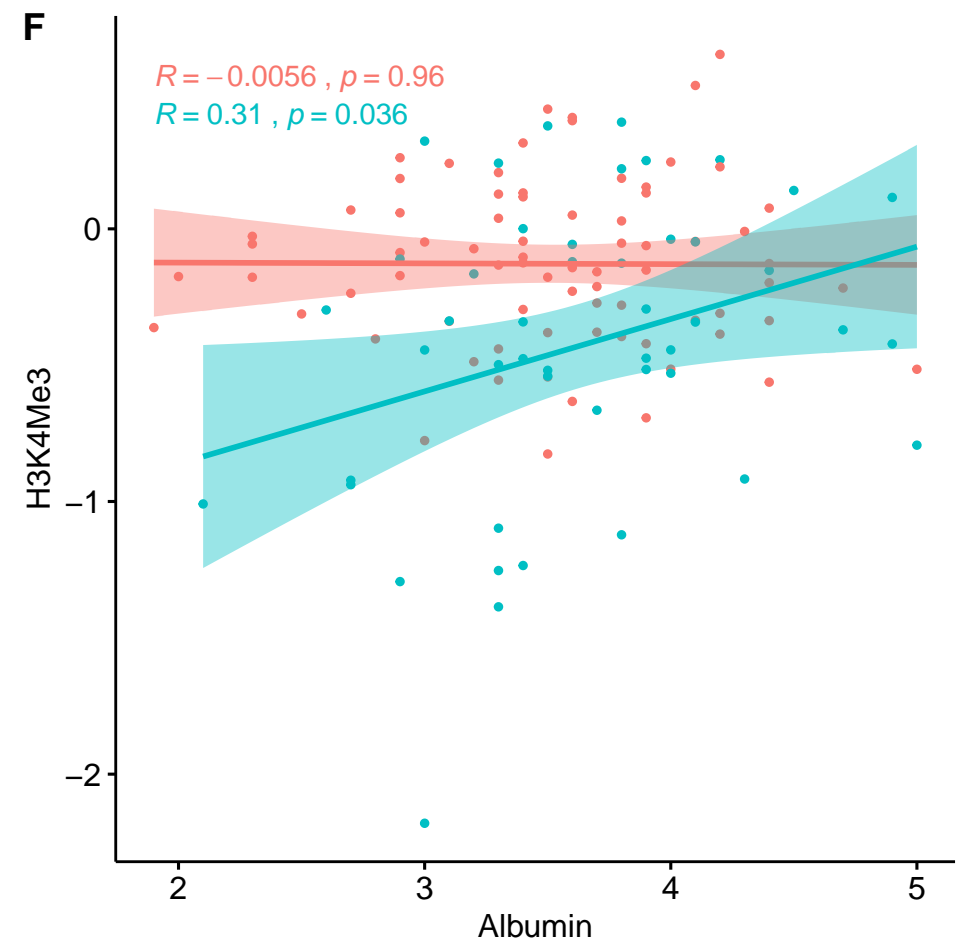

Supplement: Supplementary file 13 — Additional file 13: Figure S8. Higher expression levels of H3K4Me3 in a bone marrow (BM, n = 79) samples compared to peripheral blood (PB, n = 48) obtained from acute lymphoblastic leukemia patients (ALL, Wilcoxon, p = 0.012). H3K4Me3 positively correlated with b white blood count (WBC) in protein data from PB samples (red, r = 0.25, p = 0.025) and BM (blue, r = 0.34, p = 0.016); c absolute blast count in protein data from PB samples (red, r = 0.25, p = 0.025) and BM (blue, r = 0.35, p = 0.015); d percentage BM blasts in PB samples (red, r = 0.24, p = 0.034) and BM (blue, r = 0.65, p < 0.01); e percentage peripheral blasts in BM samples (blue, r = 0.63, p < 0.01), but not in PB samples (red, r = 0.19, p = 0.091). f Albumin levels in BM samples (blue, r = 0.31, p = 0.036). [file 13148_2021_1011_MOESM13_ESM.pdf]

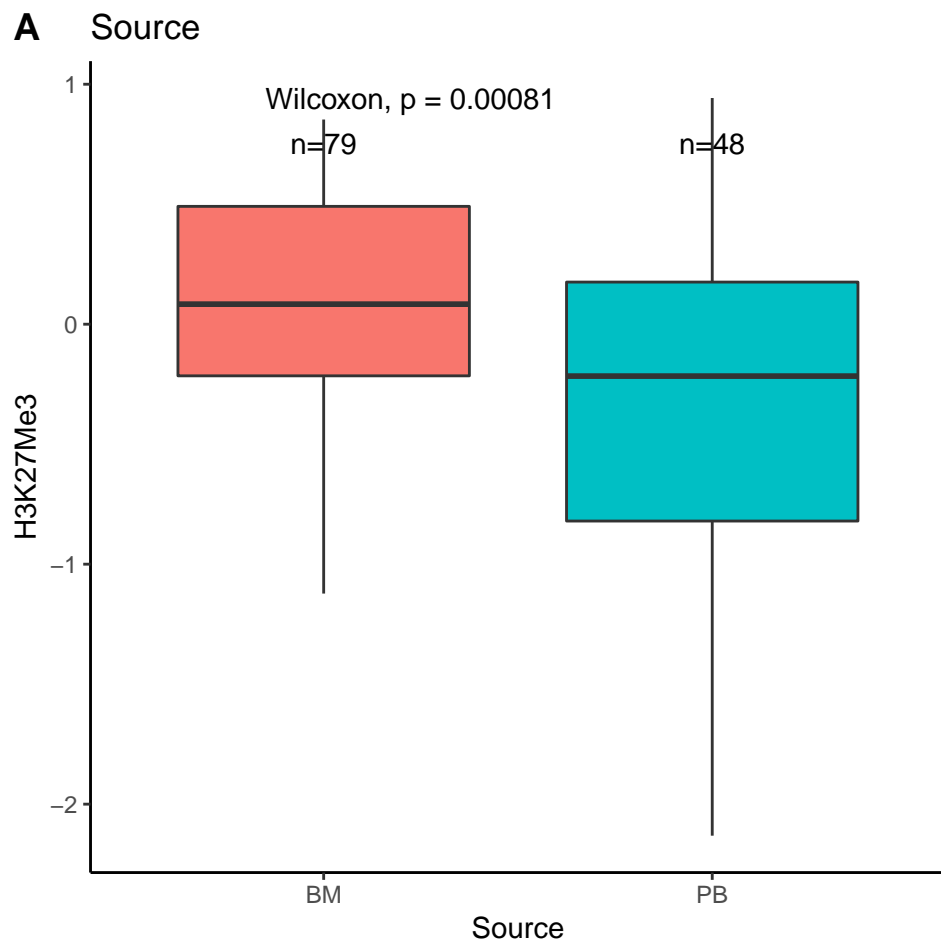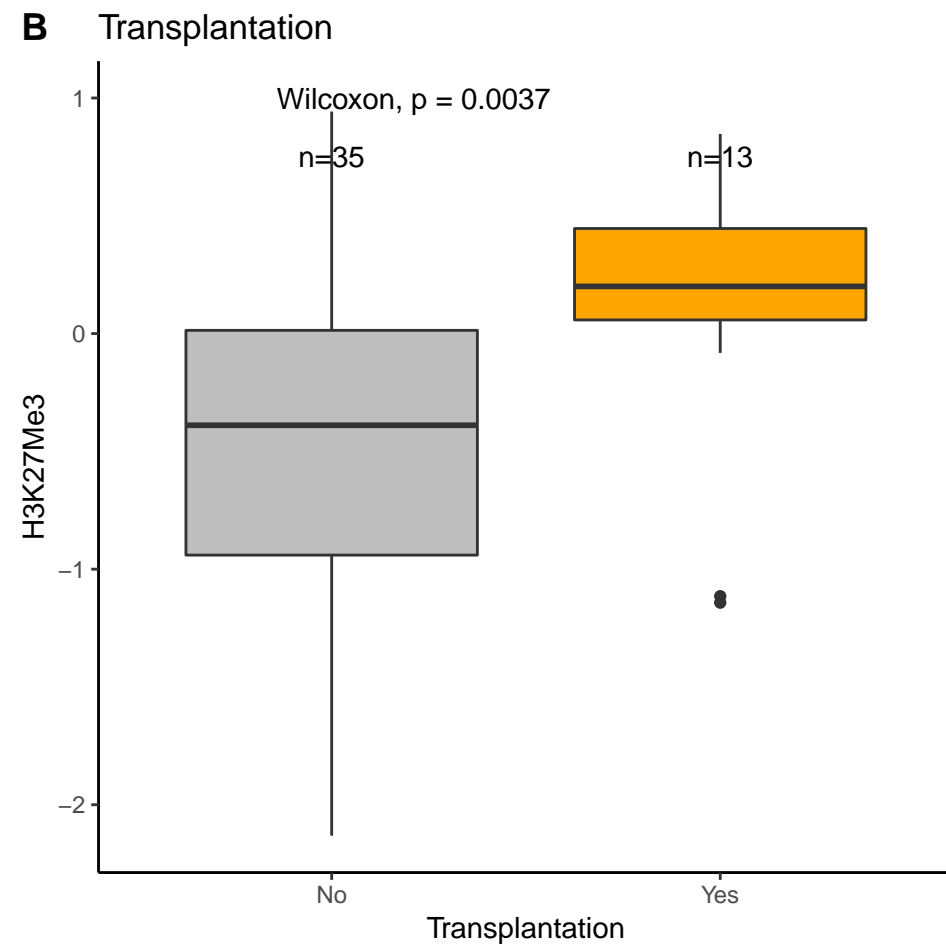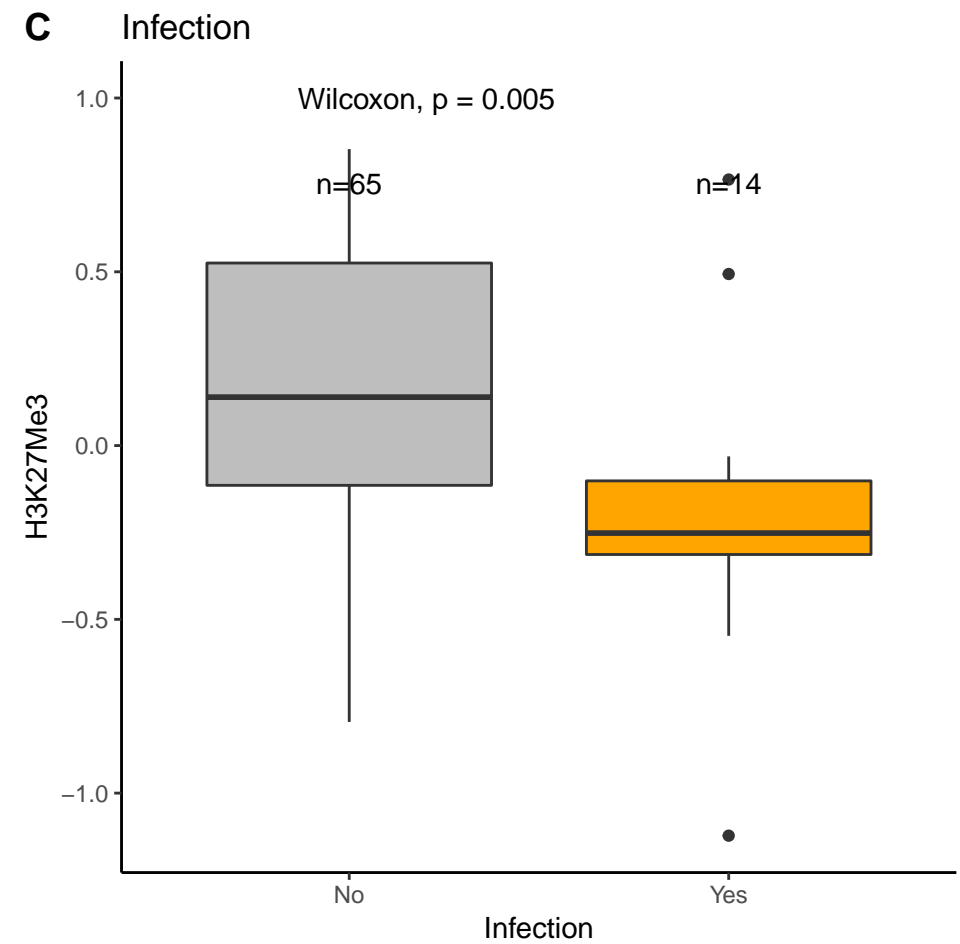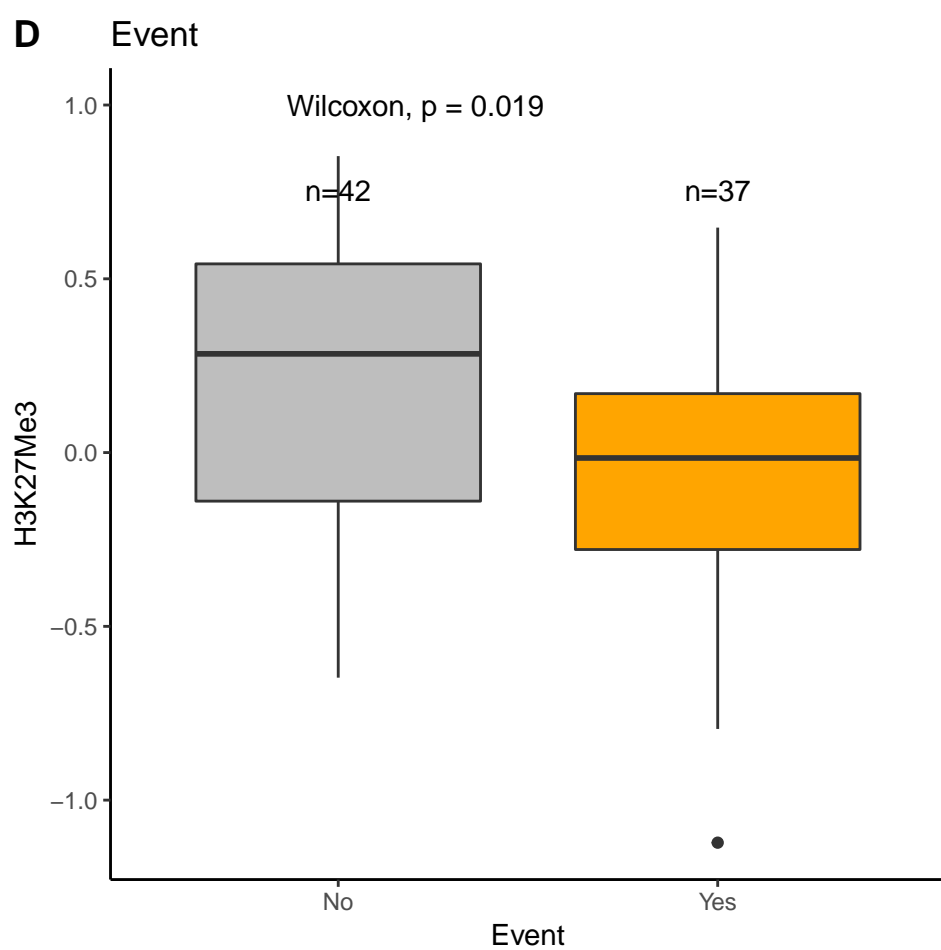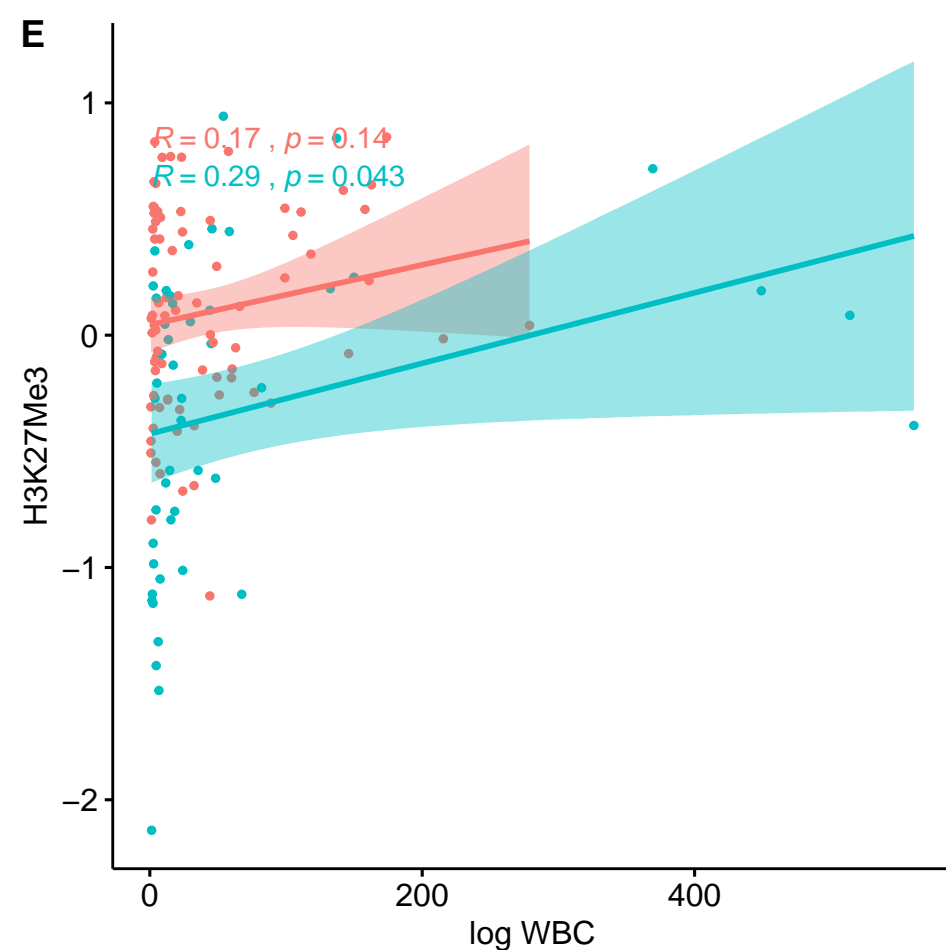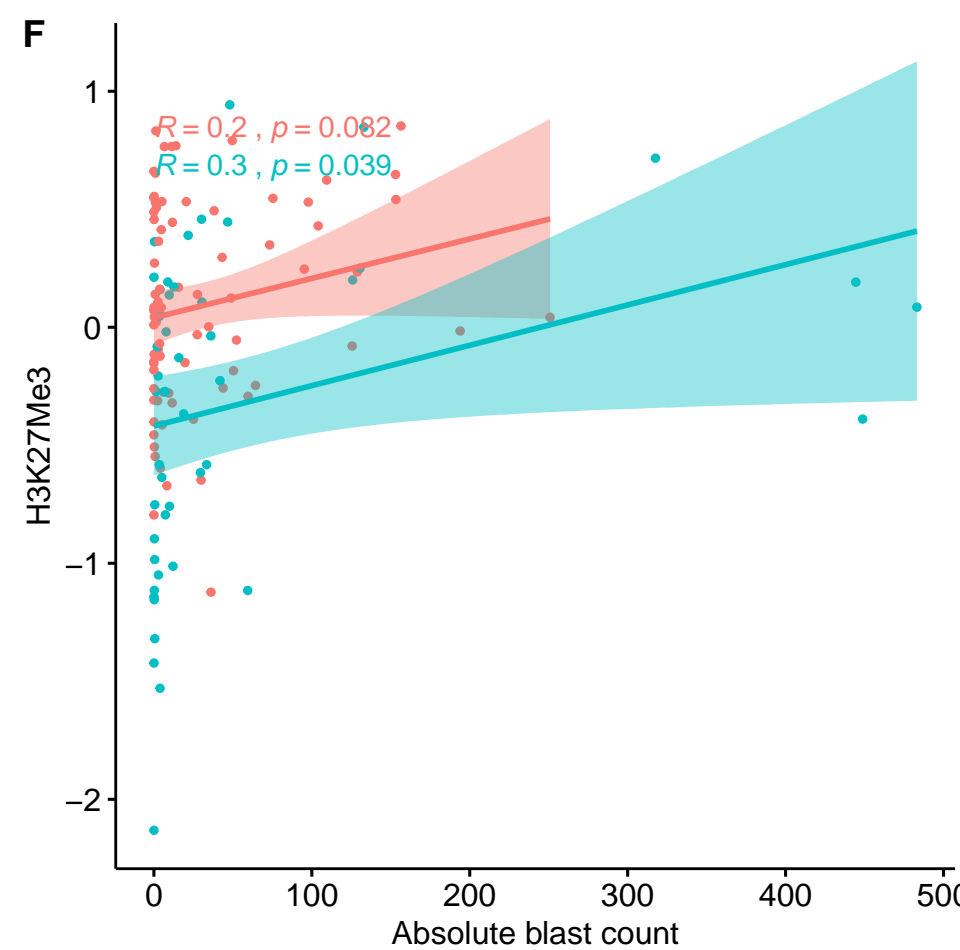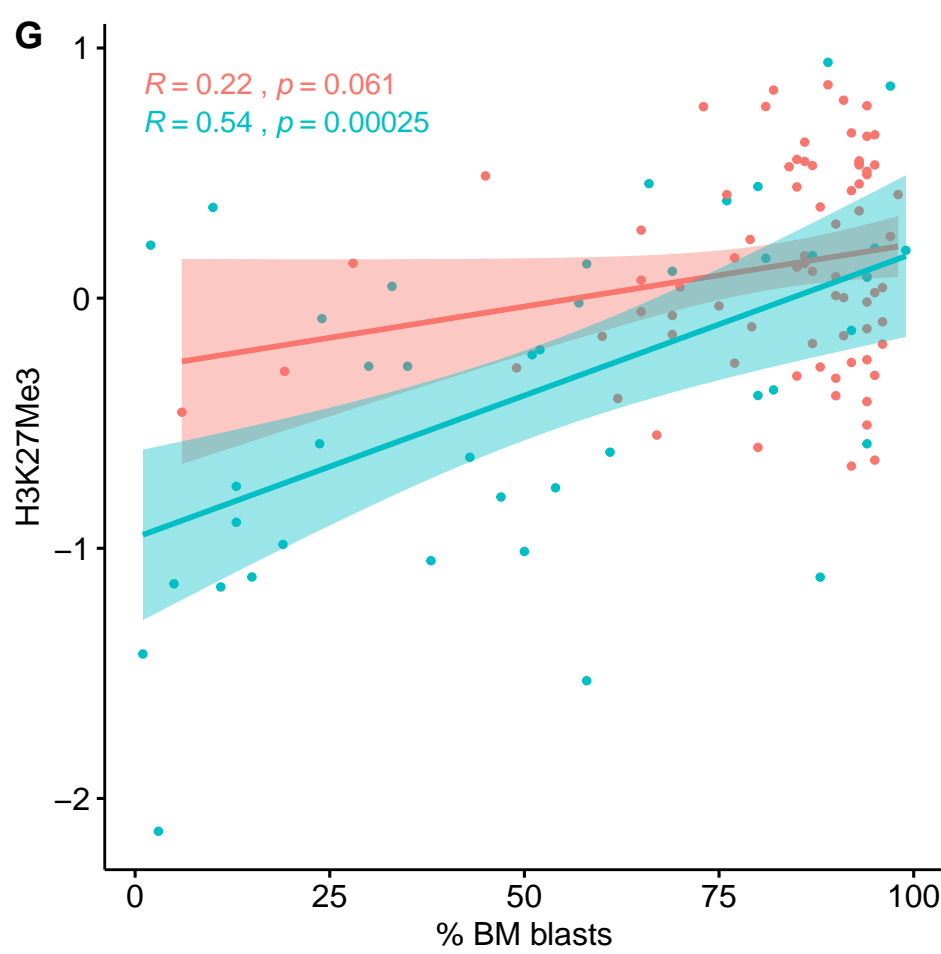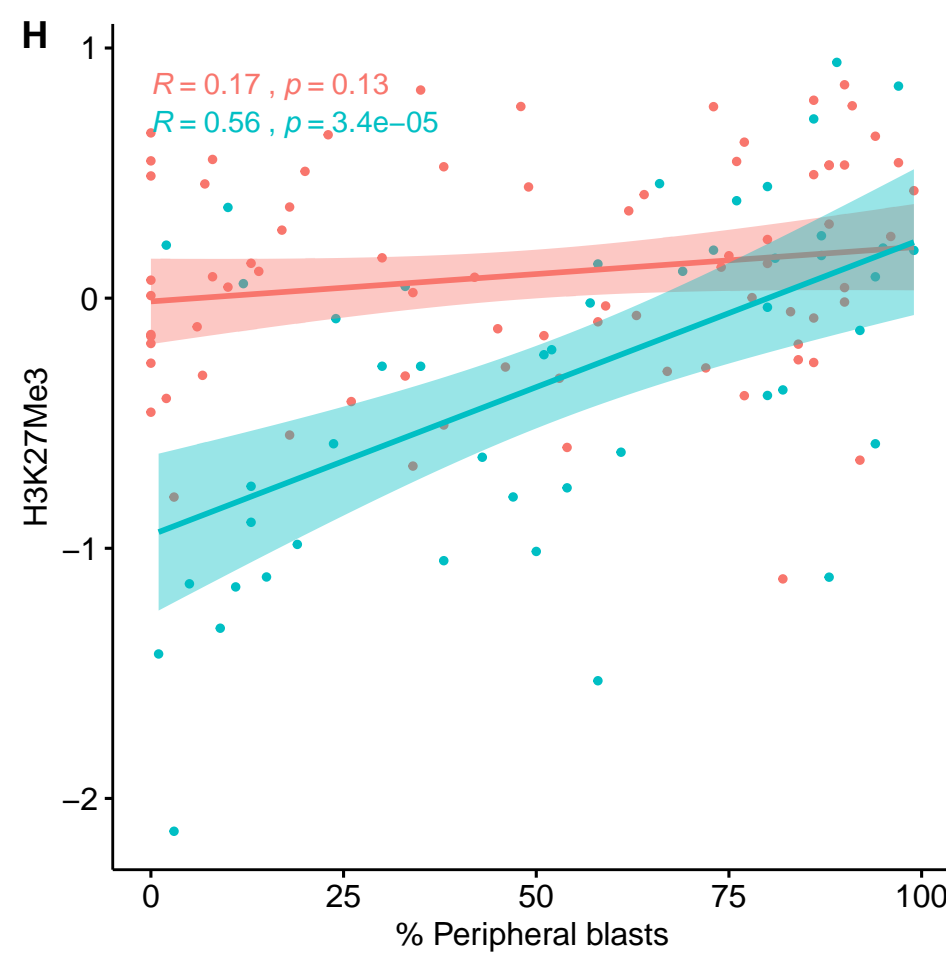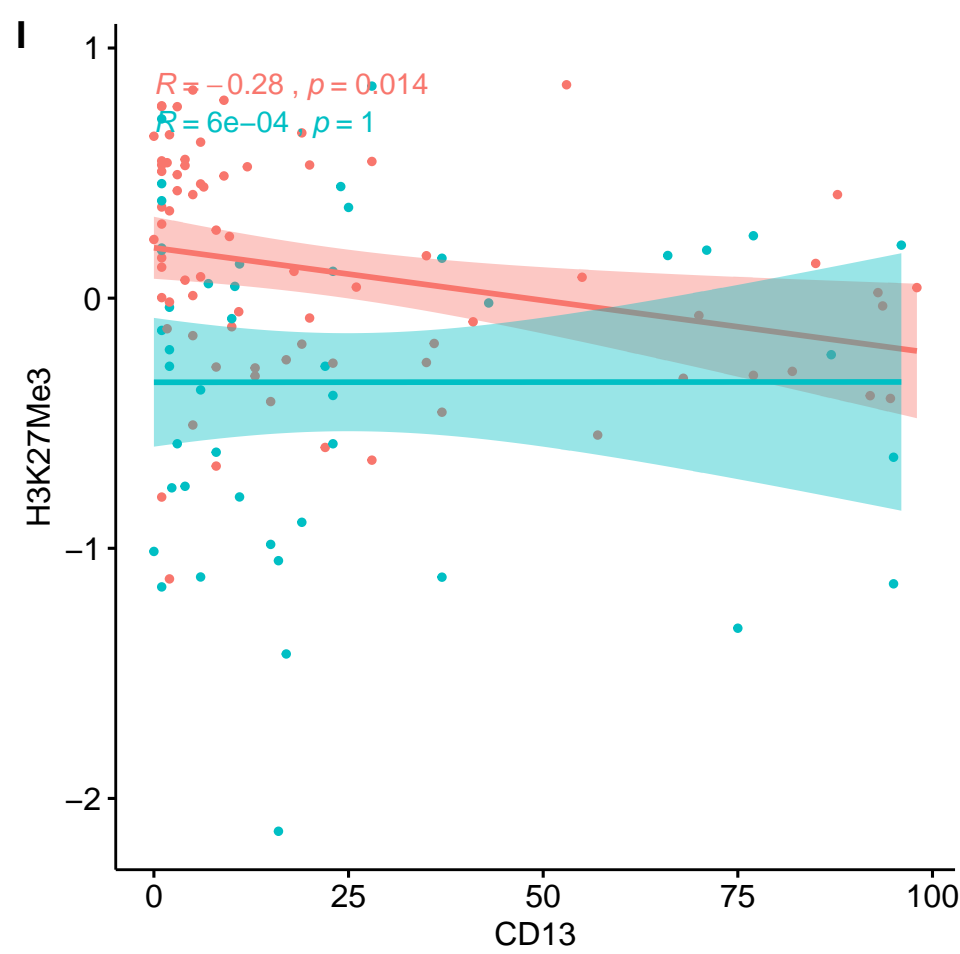

Supplement: Supplementary file 14 — Additional file 14: Figure S9. Higher expression levels of H327Me3 in a bone marrow (BM, n = 79) samples compared to peripheral blood (PB, n = 48) obtained from acute lymphoblastic leukemia patients (ALL, Wilcoxon, p < 0.01). b Higher H327Me3 in PB samples from ALL patients that underwent transplantation compared to their expression in PB samples from patients without (Wilcoxon, p < 0.01). c Lower H327Me3 was seen in 14 BM samples from ALL patients that experienced an infection compared to levels in BM samples from patients with no infection (Wilcoxon, p < 0.01) and therefore as well in patients who e experienced an event (n = 37 including 14 patients that had complicated infection, p = 0.019). H327Me3 positively correlated with e white blood count (WBC) in BM samples (blue, r = 0.29, p = 0.043), but not in the PB samples (red). f The absolute blast count in BM samples (blue, r = 0.3, p = 0.039), but not in the PB samples (red). g The percentage BM blasts in BM samples (blue, r = 0.54, p < 0.01) and PB (red, r = 0.22, p = 0.061); h the percentage peripheral blasts in BM samples (r = 0.56, p < 0.01), but not in PB samples. i H327Me3 negatively correlated with the presence of the surface marker CD13 in the PB samples (r = − 0.28, p = 0.014), but not in the BM samples. [file 13148_2021_1011_MOESM14_ESM.pdf]
