# Supplementary material for: Loss of H3K27 methylation identifies poor outcomes in adult-onset acute leukemia
Source: Clin Epigenetics. 2021 Jan 28;13:21. doi: 10.1186/s13148-021-01011-x (PMC7841917; doi:10.1186/s13148-021-01011-x)
Supplement: Supplementary file 15 — Additional file 15. Supplementary information sequencing analysis. [file 13148_2021_1011_MOESM15_ESM.docx]

**Additional file 15**

*Sequencing Analysis*

A total of 788 mutations were detected in 271 genes (Additional file 6: Table S2). The median number of mutations in each patient was 12 [6-22]. Among the 271 genes, a total of 31 AML driver mutations acting in 8 different molecular pathways were found in 100% of the patients. Molecular pathways were defined as previously (Papaemmanuil E. et al. 2016). Spliceosome (SRSF2, U2AF1, SF3B1), Chromatin (ASXL1, BCOR, KMT2A, MLL1-3, PHF6), DNA methylation (DNMT3A, TET2, IDH1-2), Transcription (MYC, CEBPA, WT1, GATA2, RUNX1(T1)), RTK-RAS signaling (NRAS, FLT3, KIT, KRAS, NF1, PTPN11, CBL), Cohesion (STAG2, RAD21), Tumor suppressor (TP53) and Nucleophosmin (NPM1). In our cohort, FLT3, NPM1, ASXL1, TET2, RUNX1 and DNMT3A mutations were most frequently mutated. The median number of driver mutations was 4 [1-10] and median involved molecular pathways was 3 [1-6] with 4 cases having mutations in one pathway (6%), 12 (18%) in two, 23 (35%) in three, 15 (23%) in four, 9 (14%) in five and 2 (3%) in six.
